# SurvBenchmark: comprehensive benchmarking study of survival analysis methods using both omics data and clinical data

**DOI:** 10.1093/gigascience/giac071

**Published:** 2022-07-30

**Authors:** Yunwei Zhang, Germaine Wong, Graham Mann, Samuel Muller, Jean Y H Yang

**Affiliations:** School of Mathematics and Statistics, The University of Sydney, Sydney 2006, Australia; Charles Perkins Centre, The University of Sydney, Sydney 2006, Australia; Sydney School of Public Health, The University of Sydney, NSW, Sydney 2006, Australia; Centre for Kidney Research, Kids Research Institute, The Children's Hospital at Westmead, NSW, 2145, Sydney, Australia; Centre for Transplant and Renal Research, Westmead Hospital, NSW, 2145, Sydney, Australia; John Curtin School of Medical Research, Australian National University, Canberra 2601, Australia; Melanoma Institute Australia, North Sydney, NSW 2065, Australia; School of Mathematics and Statistics, The University of Sydney, Sydney 2006, Australia; Department of Mathematics and Statistics, Macquarie University, Sydney 2109, Australia; School of Mathematics and Statistics, The University of Sydney, Sydney 2006, Australia; Charles Perkins Centre, The University of Sydney, Sydney 2006, Australia; Laboratory of Data Discovery for Health Limited (D^2^4H), Science Park, Hong Kong SAR, China

**Keywords:** survival analysis, machine learning, survival prediction

## Abstract

Survival analysis is a branch of statistics that deals with both the tracking of time and the survival status simultaneously as the dependent response. Current comparisons of survival model performance mostly center on clinical data with classic statistical survival models, with prediction accuracy often serving as the sole metric of model performance. Moreover, survival analysis approaches for censored omics data have not been thoroughly investigated. The common approach is to binarize the survival time and perform a classification analysis.

Here, we develop a benchmarking design, SurvBenchmark, that evaluates a diverse collection of survival models for both clinical and omics data sets. SurvBenchmark not only focuses on classical approaches such as the Cox model but also evaluates state-of-the-art machine learning survival models. All approaches were assessed using multiple performance metrics; these include model predictability, stability, flexibility, and computational issues. Our systematic comparison design with 320 comparisons (20 methods over 16 data sets) shows that the performances of survival models vary in practice over real-world data sets and over the choice of the evaluation metric. In particular, we highlight that using multiple performance metrics is critical in providing a balanced assessment of various models. The results in our study will provide practical guidelines for translational scientists and clinicians, as well as define possible areas of investigation in both survival technique and benchmarking strategies.

## Background

Survival models are statistical models designed for data that have censored observations, that is, time-to-event data, which are ubiquitous, including in health, tourism [1], economics [2], and engineering [3]. In this paper, we will follow the terminology of survival analysis in which the event of interest is captured through a “status” variable, “s” typically, considered a binary class outcome. The waiting time to this status event is defined as the “survival” time, either measured as continuous or discrete time periods. Survival models target both outcomes: status and time-to-event, whereas neither regression analysis on time nor classification analysis on status explain this bivariate outcome information [4]. Classes of models dealing with these events have wide applicability well beyond the clinical and omics applications that are considered here.

Numerous survival models have been developed over the last decades. There are many studies in the literature that give a good overview on right-censored data sets without time-dependent covariates, for example [5]. However, few of these studies take a practical viewpoint, and few make sufficient real-world data set comparisons, particularly in the biomedical field. This motivated us to develop a benchmarking design for the diverse clinical and omics survival data in health. This work intends to improve the knowledge and understanding of such models and guide clinical decision-making. We first performed an exhaustive search for various types of available survival analysis methods and the methods of performance evaluations for the different types of data sets.

Among the comparative studies that included real-world data sets in health, we found that most have a specific focus such as on a certain disease (e.g., colon cancer) or on a certain data platform (e.g., omics or clinical). For example, Schober and Vetter [6] and Ahmed et al. [7] conduct reviews on classical survival models such as the Kaplan–Meier (KM) method and the Cox Ppoportional Hhzards (CoxPH) model with a focus on clinical data with an induced anasthesia state and a specific colon cancer type, respectively. Lee and Lim [8] apply the penalized Cox model, survival support vector machine (SVM), random survival forest (RSF), and Cox boosting models on large genomic data. To date, no systematic review encompasses data sets obtained from multiple disease types. Therefore, this necessitates the development of a benchmarking design that will provide a better understanding of how different survival models perform in practice across various disease types.

With the emergence of different modeling approaches from various disciplines, many of these recent comparison studies have limited their focus on either within classical models (KM method, CoxPH model) or within modern machine learning (ML) methods. Recently, a comprehensive survey article [5] compares three categories of statistical survival and ML methods with a focus on theoretical mathematical details. However, this study does not provide practical implications of the various methods, and no comparison of performance using real-world data sets is made. There is a need for better guidance on what data analysis strategy to use.

A recent exception is the benchmark study by Herrmann et al. [9]. This valuable contribution includes both real-world clinical and omics data sets and analyzes these with classical regression and modern ML methods with particular focus on the impact of considering the multi-omics structure to the survival model predictability. However, this study includes cancer diseases only, and data sets are solely obtained from The Cancer Genome Atlas. There remains a pressing need to look into more diverse and thus more heterogeneous data sets coming from multiple databases to benchmark the survival model performances from more diverse aspects.

To this end, we develop a benchmarking design SurvBenchmark that considers multiple aspects with several evaluation metrics on a large collection of real-world health and biomedical data sets that guides right-censored data survival method selection and new method development.

## Survival Models and Their Evaluation

Survival models can deal with data that explain censored observations with a bivariate outcome variable, consisting of “time” (the minimum of “time-to-event” and “censoring time”) and “event” (binary: “class 1” = “event did occur,” “class 0” = “otherwise”). There are two key features of such censored survival objects. First, the class label “0” means an observation is censored as its exact event time is not observed. Second, an additional tracking time measurement is included as part of the response.

There are two main branches of survival models: classical statistical survival models, which include parametric, nonparametric, and semiparametric models, and modern ML survival models, which include ensemble-based methods and state-of-the-art deep learning–based approaches. Both sets of models are briefly reviewed in the following sections.

### Classical survival models

The CoxPH model [10] is the most widely used classical survival model. CoxPH works on the hazard function, which is given by

(1) $$\begin{eqnarray*}
h\left( {t,x} \right) = {h_0}\left( t \right){e^{\left( {\sum\nolimits_{j = 1}^p {{\beta _j}{x_j}} }\right)}}
\end{eqnarray*}
$$

where $x\,\, = {\rm{\,\,}}( {{x_1},{x_2}, \ldots ,{x_p}} )$ is the covariate vector and ${h_0}( t )$ is the baseline hazard function. CoxPH is a semiparametric model and the baseline hazard function is canceled out when taking the ratio of 2 hazard functions.

The penalized Cox model is another extension of the CoxPH model that helps to prevent overfitting. The L1 regularized CoxPH model adds a scaled sum of absolute values of the magnitude of model coefficients, that is, ${\lambda _1}\mathop \sum \limits_{j\,\, = {\rm{\,\,}}1}^p | {{\beta _j}} |{\rm{\,\,}}$, as the regularization term to the partial log-likelihood. Other regularizers can be used such as L2 regularization, that is, ${\lambda _2}\mathop \sum \limits_{j\,\, = {\rm{\,\,}}1}^p {({\beta _j})^2}$, or other scaled sums of nonnegative penalties of the $\beta {\rm{^{\prime}}}s$, such as in the following general penalized partial log-likelihood:

(2) $$\begin{eqnarray*}
\log \left( {L(\beta)} \right){\rm{ - \,\,}}\lambda \sum\nolimits_{j = 1}^p {\pi \left( {{\beta _j}} \right)} ,
\end{eqnarray*}
$$

where $L( \beta )$ is the partial likelihood as, for example, given in Tibshirani [11] (Equation 2) and then optimization takes place [12, 13,14]. Using the L1 penalty in Equation (2) gives the Lasso Cox estimation and using the L2 penalty gives the Ridge Cox solution, respectively. If instead of a single regularization term, we consider a weighted average of the L1 and L2 penalty, we obtain the Elastic Net Cox model. One remarkable characteristic of the Lasso Cox model and the Elastic Net Cox model is that they can simultaneously perform feature selection and prediction, because some of the beta parameters can be penalized all the way to 0 when maximizing (2). Notice that the various types of regularization terms can also appear in the loss function of modern ML methods, which we introduce in the next subsection.

### Modern machine learning models

There has been a growing interest in the use of modern ML methods in health as a result of their exceptional performance in many other areas, such as in finance [15], environment [16], and internet of things [17]. Notable examples in health include the application of RSF on complex metabolomics data [18] and SurvivalSVM to the survival of prostate cancer patients [19]. Both approaches are survival analysis extensions to two widely used ML algorithms for binary classification, namely, random forest and SVM.

SurvivalSVM was developed by Van Belle et al. [20] for time-to-event data. It is a variant of the regularized partial log-likelihood function (2) above but has a different penalty term. In contrast to using $\lambda \mathop \sum \limits_{j\,\, = {\rm{\,\,}}1}^p \pi ( {{\beta _j}} )$, SurvivalSVM uses penalized splines and then applies both, ranking constraints and regression constraints to the corresponding partial log-likelihood function. SVM with those constraints enables models for high-dimensional omics data to have more flexible structure, for example, additive (non)linear models. One distinct feature of SurvivalSVM is that it treats the prognostic problem as a ranking problem and therefore the estimation of the hazards is not directly incorporated in the model.

RSF was first proposed by Ishwaran et al. [21] as an extension of random forest to model censored survival data. Random forest [22] is a nonparametric bagging-based ensemble learning method that adds variation in the training data sets by bootstrapping the data. Multiple models are generated based on many resamples. The ensemble prediction result is then an average of these multiple models or the result of a majority vote. The key components in our application of RSF are that we use Harrell's C-index to evaluate the survival tree instead of the mean square error for regression problems or confusion matrix for classification problems and that we use the log-rank score in each node as the stopping rule.

Another ensemble-based approach is the boosting method, which contains multiple learners and sequentially gives more weight to weak learners to enhance predictability. For example, the Cox boosting model [23, 24, 25, 26] is developed based on Cox models with boosting being applied to the estimation of the regression parameter vector $\beta $ in Equation (1). There are two popular approaches to update $\beta $: the first is the model-based approach that leads to the mboost method, and the second is the likelihood-based approach that leads to the CoxBoost method (benchmarked in this study).

These models so far only focus on optimizing a single objective. Because survival data are time dependent, it is natural to have multiple tasks related to one or more time points of interest. This naturally leads to multitask learning, a method that deals with the need to predict for more than a single response variable, based on joint optimization of multiple likelihood functions corresponding to each task. The multitask logistic regression model (MTLR) by Yu et al. [27] is a survival model for multiple time points, where for each, the task is to predict survival using a logistic regression model and the parameters from each model are estimated simultaneously in the maximization of the joint likelihood function.

More recently, the ML and artificial intelligence communities refer to the methods described above as classical ML methods due to the emergence of deep learning (DL), a conceptual advancement based on neural networks (NN). In survival analysis, a number of DL survival models were developed such as Cox-nnet [28], DeepSurv [29], and DeepHit [30]. The key concept here is having different loss functions that particularly target either the hazard or the survival probability for those neurons in hidden layers when building the DL architecture. High-dimensional complex biological information can be better represented with the application of those hidden layers [31] and through relaxing the proportional hazard assumption.

### Feature selection methods applied to survival models

The input features are fundamental to every statistical or machine learning model, and the survival model is no exception [32]. Wrapper and filter [33] are two feature selection methods that are widely used for not only regression and classification models but also survival models.

The wrapper approach is a model-dependent method in which the performance of the model determines the selection of subsets of features. Stepwise feature selection approaches fall into this category since one feature is deemed to be included or deleted as the model's performance improves. Other more computational approach such as the genetic algorithm (GA) [34], which was originally developed to solve an optimization problem, has been extended to use as a feature selection approach [35]. The main idea is to start with an initial set of features to then replace it with one that includes features from other parts of the data to optimize the classification accuracy based on a linear discrimination analysis model.

The filtering approach, on the other hand, is a model-independent feature selection method that produces a subset of features without involving the models. This step often occurs outside and before building prediction models. Many of these strategies select features using hypothesis testing statistics from a univariate study. With the advent of omics data in the 1990s, the statistics community embraced the development of differential expression (DE) analysis, which is a filter-type feature selection method for identifying promising genes/features using “parallel univariate strategies” based on linear modeling [36].

### Classical performance evaluation metric for survival data

Classically, survival analysis is evaluated in 3 broad settings: the concordance index, the Brier score, and the time-dependent area under the curve (AUC). Similar to evaluating classification and regression models, metrics for calibration and discrimination are developed with incorporating censoring by applying rank-based methods or error-based methods together with a weighting scheme.

#### C-index and its extension in survival analysis

C-indices in survival analysis are concordance-based methods, where “concordance” measures how close a prediction is to the truth. The original C-index for survival analysis was introduced by Frank E. Harrell [37] as a time-independent performance measure. C-indices range from 0 to 1, where 1 means perfect performance and 0 means worst possible performance. If a model would not take into account any information from the data, that is, a random prediction is made, then the corresponding C-index would be around 0.5. For most clinical data sets, a C-index around or larger than 0.6 is considered an acceptable prediction. Harrell's C-index [38] defines concordance by looking at ranks of pairs of subjects in the data (there are *n* choose 2 pairs for data with *n* subjects). Harrell's C-index further depends on the censoring distribution of the data, is motivated by Kendall's tau statistic, and is closely related to Somers’ D. When ranking the subjects, censored subjects are excluded, and pairs included in the formula are only those comparable, noncensored pairs. There are different versions of the C-index, where the differences come from the different ways that censored subjects are ranked.

We will use the following 3 concordance indices: Begg's C-index, Uno's C-index, and GH C-index. First, Begg's C-index [39] uses KM estimation to incorporate both censored and uncensored subjects by assigning different weight to them. Second, Uno et al. [40] develop a new way to calculate the rank with the help of inverse probability of censoring weight (IPCW). Third, the GH C-index [41] changes the concordance function into a probability function based on the Cox model estimation and then approximates its distribution, which is robust to censoring.

#### Brier score

The Brier score [42, 43] uses IPCW to handle censored subjects when measuring discrepancies between the estimated values and the actual values. This score can be considered a similar measure to the mean squared error (MSE) in regression models to some extent. Like the MSE, the Brier score takes a value greater than 0 that depends on the data, and the smaller the Brier score, the better. However, to have better interpretability, the integrated Brier score (IBS) is introduced, which also takes values between 0 and 1– it averages the loss over time in situations where there is no interest in a particular time point but performance is with regards to all time points as a whole.

#### Time-dependent AUC

The time-dependent AUC is inspired from binary classification model evaluations. The receiver operating characteristic (ROC) curve is a classical model assessment plot that examines the relationship between the sensitivity and the false-positive rate. The area under the ROC curve is termed *AUC*. In survival analysis, event statuses are changing over the time, which requires a dynamic measurement to discriminate the predicted versus the actual. Chambless and Diao [44] were the first to propose a time-dependent AUC for survival analysis. They define the AUC(*t*) as the probability that a person with disease onset by time *t* has a higher score than the person with no event by time *t*. Changes of model predictability for different time points can therefore be visualized by time-dependent AUC curves, which allows people to compare long time versus short time predictability.

## Materials and Methods

### Data sets: 6 clinical and 10 omics data sets

Clinical data sets—6 clinical data sets with different sample sizes and disease types are selected (see references in Table 1).

- Veteran data is a survival data set from the randomized trial of 2 treatment regimens for lung cancer obtained from the R package “survival.” There are 6 measured features in this data.
- PBC data (5 clinical features, 312 patients) from the Mayo Clinic trial in primary biliary cirrhosis (Pbc) of the liver conducted between 1974 and 1984; obtained from the R “RandomForestSRC.”
- Lung data (7 features, 228 patients) contains patient survival information with advanced lung cancer from the North Central Cancer Treatment Group and is available from the R package “survival.”
- ANZ data (ANZDATA): Australia & New Zealand Dialysis and Transplant Registry data containing graft survival information and electronic clinical records for kidney transplantation recipients in Australia and New Zealand from 30^th^ June 2006 to 13^th^ November 2017. This data contains records for both living and deceased donors and also multiorgan transplants. We processed the raw data, restricting the transplant date to be after 18^th^ September 2008 and retained deceased donor kidney transplants only. Missing records are excluded, resulting in 3,323 patients and 38 features containing patient, donor, and donor–recipient human leukocyte antigen (HLA) compatibility.
- UNOS_Kidney data: Organ transplant data based on the Organ Procurement and Transplantation Network (OPTN)–United Network for Organ Sharing (UNOS) in the United States (based on OPTN data as of March 2020). We selected a random sample of 3,000 records associated with deceased donor kidney transplantation only with 99 features containing recipients, donors, and donor–recipient HLA compatibility. Missing values are imputed using the R package “MICE.”
- Melanoma_clinical data, extracted from melanoma data [45, 46]: A in-house data set collected as a part of a multi-omics study. This is the part that contains clinical information for patients. After deleting all missing values, we have 88 patients with stage III melanoma disease measured by 14 clinical features.

**Table 1:** Data sets summary

Data sets summary					
Data set (name used in this paper)	No. of observations	No. of variables	Type of data	Censoring rate (rounded to 4 decimal places)	Reference
Melanoma_itraq	41	642	Omics	0.4146	Wang KYX et al. Cross-Platform Omics Prediction procedure: a game changer for implementing precision medicine in patients with stage-III melanoma. bioRxiv 2020.12.09.415927; doi: https://doi.org/10.1101/2020.12.09.415927
Melanoma_nano	45	206	Omics	0.4222	Wang KYX et al. Cross-Platform Omics Prediction procedure: a game changer for implementing precision medicine in patients with stage-III melanoma. bioRxiv 2020.12.09.415927; doi: https://doi.org/10.1101/2020.12.09.415927
Ovarian_2	58	19,818	Omics	0.3793	Ganzfried BF et al. (2013) curatedOvarianData: clinically annotated data for the ovarian cancer transcriptome. Database, 2013.
GE_5	78	4,753	Omics	0.5641	van ’t Veer LJ et al. (2002) Gene expression profiling predicts clinical outcome of breast cancer. Nature, 415, 530–536.
GE_3	86	6,288	Omics	0.7209	Bullinger L et al. (2004) Use of gene-expression profiling to identify prognostic subclasses in adult acute myeloid leukemia. N Engl J Med, 350, 1605–1616.
Melanoma_clinical	88	16	Clinical	0.3939	Wang KYX et al. Cross-Platform Omics Prediction procedure: a game changer for implementing precision medicine in patients with stage-III melanoma. bioRxiv 2020.12.09.415927; doi: https://doi.org/10.1101/2020.12.09.415927
GE_1	115	551	Omics	0.6670	Sorlie T et al. (2003) Repeated observation of breast tumor subtypes in independent gene expression data sets. Proc Natl Acad Sci U S A, 100, 8418–8423.
GE-_4	116	4,753	Omics	0.5641	van de Vijver MJ et al. (2002) A gene-expression signature as a predictor of survival in breast cancer. N Engl J Med, 347, 1999–2009.
Veteran	137	8	Clinical	0.0657	Kalbfleisch JD and Prentice RL (2002) The statistical analysis of failure time data. Wiley Series in Probability and Statistics.
Ovarian_1	194	16,050	Omics	0.7062	Ganzfried BF et al. (2013) curatedOvarianData: clinically annotated data for the ovarian cancer transcriptome. Database, 2013.
Lung	228	9	Clinical	0.2763	Loprinzi CL et al. (1994) Prospective evaluation of prognostic variables from patient-completed questionnaires. North Central Cancer Treatment Group. J Clin Oncol, 12, 601–607.
GE_6	240	7,401	Omics	0.4250	Van Houwelingen HC (2004) The elements of statistical learning, data mining, inference, and prediction. Trevor Hastie, Robert Tibshirani, and Jerome Friedman, Springer, New York, 2001. Stat Med, 23, 528–529.
GE_2	295	4,921	Omics	0.7322	Beer DG et al. (2002) Gene-expression profiles predict survival of patients with lung adenocarcinoma. Nat Med, 8, 816–824.
PBC	312	7	Clinical	0.5994	Fleming TR and Harrington DP (2005) Counting processes and survival analysis. Wiley Series in Probability and Statistics.
UNOS_Kidney	3,000	101	Clinical	0.7350	OPTN data (https://optn.transplant.hrsa.gov/)
ANZ	3,323	40	Clinical	0.8739	ANZDATA (https://www.anzdata.org.au/)

Data table showing the names of data sets used in this paper in the first column. Data sets are ordered by the number of observations (second column, from smallest to largest). Censoring rate is rounded to 4 decimal places.

Omics data sets—We consider 8 published data and 2 in-house melanoma cancer data sets. A summary of the size and censoring rate of all data sets can be found in Table 1.

- Two ovarian cancer gene expression data sets, downloaded from the R package “curatedOvarianData.” Curation and analysis pipeline of this data follow [47]. Ovarian1 is the “GSE49997_eset” data, and Ovarian2 is the “GSE30161_eset” data.
- Another 6 gene expression data sets are available online from work by Yang and colleagues [48]. We have named them GE1 to GE6 for easier rendering of labels in our figures. For GE_3, log2 transformation is applied, followed by a k-nearest neighbor imputation method (KNN) imputation with 10 nearest points. For GE_6, median normalization is applied. For others, no further preprocessing was performed.
- Melamona_itraq and Melanoma_nano are 2 in-house melanoma omics data sets [45, 46]. The first is a protein expression data set from the iTRAQplatform and the second is a Nanostring data set from the above melanoma study, and preprocessing steps are described in the respective papers. The itraq protein expression data has 41 patients with 640 proteins. The nanostring data has 45 patients with 204 genes [49], and the GEO ID is “GSE156030."

### Benchmarking design/procedure

Evaluation metrics: We examine model performance metrics that can be broadly grouped into 4 categories and assess performance in terms of each method’s flexibility, predictability, stability, and computational efficiency, detailed in Supplementary Table S1 and briefly summarized as follows:

- We measure *model flexibility* by looking at whether a given method can handle different data modality, different level of sparsity, and represents multiple ways, including the type of data required (clinical, omics), type of input required (categorical, numerical), sparsity of the data allowed (yes, no), and prediction ability evaluation metrics allowed.
- We measure *model predictability* using 3 different metrics: C-index, time-dependent AUC, and Brier score. We apply 4 different modified versions of the C-index: Harrell's C-index, Begg's C-index, Uno's C-index, and GH C-index. For identification of different time points, we equally divided the survival time ranging from the first quartile to the third quartile into 15 time points for each data set, and therefore, we obtained 15 AUC values corresponding to each time point. As for the Brier score, we calculated the raw Brier score and the IBS. The raw Brier score is calculated by taking the sum of all Brier scores for all event times in the data set.
- We measure the *model computational efficiency* using both computational time and memory. Computational time is calculated using the “Sys.time” function in R. Memory is calculated using the “Rprof” function in R, and the total memory used is summarized for each experiment.
- We measure *model stability* using model reproducibility and the standard deviation (SD) of model predictability metrics. Model reproducibility is defined as the proportion of successful runs among all the runs attempted. For each model predictability metric, we calculated its SD. We then ranked the values for all the methods for each data set from the most stable (smallest SD) to the least stable (largest SD).

Benchmarking methods: All methods evaluated are described in Table 2 (details in Supplementary Data). In this benchmark study, hyperparameter sets used in these methods are chosen to be the default set. All compared methods (Supplementary Data) and evaluation metrics (Supplementary Table S1) are applied and evaluated on real-world data sets listed earlier. We apply 20 times (runs) repeated 5-fold cross validation using RStudio server (RRID:SCR_000432) with 15 cores in parallel. For each run, the whole data is split into a training data set (80%) and a testing data set (20%) with each method trained using the training data set and values of evaluation metrics calculated using the testing data set. For methods with a feature selection step, a nested feature selection step is applied on those training folds within each 5-fold cross-validation procedure. Detail about the packages and parameters can be found in the Supplementary Data, and functions used to evaluate the methods are shown in Supplementary Table S1.

**Table 2:** Summary of methods used in this study

Method name	Method name in this paper	R function name	R package name	Parameters (default)
Cox	Cox	coxph	survival	NA
Cox with backward elimination using AIC	Cox_bw_AIC	cph, fastbw	rms	rule = “aic,” sls = .05, k.aic = 2
Cox with backward elimination using *P* value	Cox_bw_p	cph, fastbw	rms	rule = “p,” sls = .05
Cox with backward elimination using BIC	Cox_bw_BIC	cph, fastbw	rms	rule = “aic,” sls = .05, k.aic = log(as.numeric(table(train$status)[2]))
Lasso Cox (for clinical data sets)	Lasso_Cox	penalized	penalized	Lambda1 = 1, lambda2 = 0
Ridge Cox (for clinical data sets)	Ridge_Cox	penalized	penalized	Lambda1 = 0, lambda2 = 1
Elastic Net Cox (for clinical data sets)	EN_Cox	penalized	penalized	Lambda1 = 1, lambda2 = 1
Lasso Cox (for omics data sets)	Lasso_Cox	glmnet	glmnet	alpha = 1, nfolds = 5, type.measure = “C”
Ridge Cox (for omics data sets)	Ridge_Cox	glmnet	glmnet	alpha = 0, nfolds = 5, type.measure = “C”
Elastic Net Cox (for omics data sets)	EN_Cox	glmnet	glmnet	alpha = 0.5, nfolds = 5, type.measure = “C”
Random survival forest	RSF	rfsrc	RandomSurvivalForest	Default: ntree = 1000, mtry = 10
Multitask logistic regression method	MTLR	mtlr	MTLR	C1 = 1
DNNSurv (deep learning survival model)	DNNSurv	multiple functions as in Github codes	DNNSurv	Default: no parameter arguments to be changed by users
Boosting Cox model	CoxBoost	coxboost	CoxBoost	stepnumber = 10, penalty number = 100
Cox model with genetic algorithm as feature selection method	Cox (GA)	GenAlg	GenAlgo	n.features = 10 (for omics), n.features = 4 (for clinical), generation_num = 20
Multitask logistic regression model with genetic algorithm as feature selection method	MTLR (GA)	GenAlg	GenAlgo	n.features = 10 (for omics), n.features = 4 (for clinical), generation_num = 20
Boosting Cox model with genetic algorithm as feature selection method	CoxBoost (GA)	GenAlg	GenAlgo	n.features = 10 (for omics), n.features = 4 (for clinical), generation_num = 20
Multitask logistic regression model with ranking-based method as feature selection method	MTLR (DE)	lmFit,eBayes	limma	n.features = 10 (for omics), n.features = 4 (for clinical)
Boosting Cox model with ranking-based method as feature selection method	CoxBoost (DE)	lmFit,eBayes	limma	n.features = 10 (for omics), n.features = 4 (for clinical)
Survival support vector machine	SurvivalSVM	survivalsvm	survivalsvm	Default: sgf.sv = 5, sigf = 7, maxiter = 20, margin = 0.05, bound = 10, eig.tol = 1e-06, conv.tol = 1e-07, posd.tol = 1e-08
DeepSurv (deep learning survival model)	DeepSurv	deepsurv	survivalmodels	Default: frac = 0.3, activation = “relu,” num_nodes = c(4 L, 8 L, 4 L, 2 L), dropout = 0.1, early_stopping = TRUE, epochs = 100 L, batch_size = 32L
DeepHit (deep learning survival model)	DeepHit	deephit	survivalmodels	Default: frac = 0.3, activation = “relu,” num_nodes = c(4 L, 8 L, 4 L, 2 L), dropout = 0.1, early_stopping = TRUE, epochs = 100 L, batch_size = 32L

Data table showing the methods used in this benchmark study. R packages and functions with parameters are listed.

## Results

### Comprehensive benchmarking design

**Figure 1:**
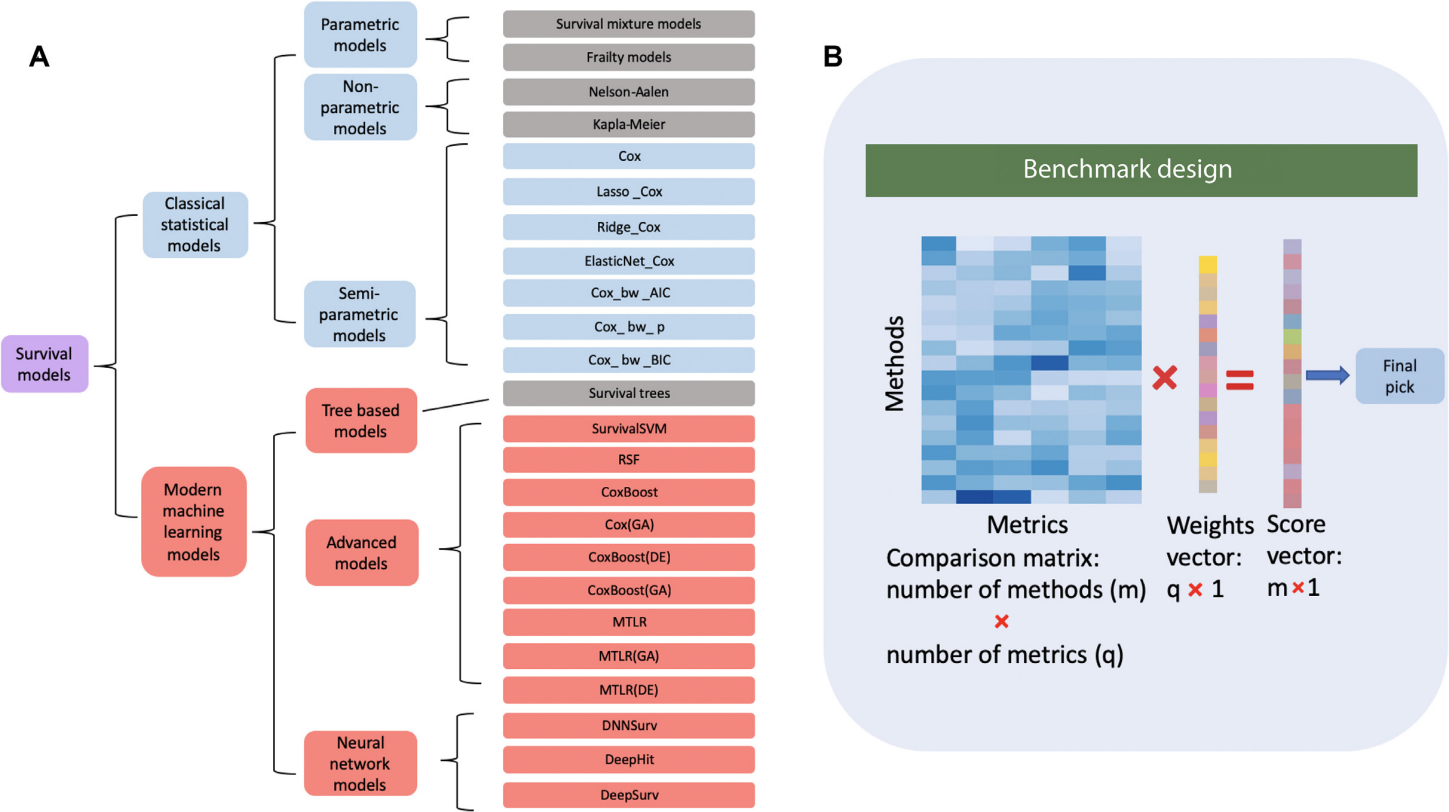
SurvBenchmark: schematic view of our benchmark design. (A) An overview of survival methods used in this study. We broadly classify current models into 2 categories: classical statistical models (top group) and modern machine learning models (bottom group), which is inspired by the study from Wang et al. [2]. Each of these categories can be further subdivided as presented in the hierarchical chart. All models in blue and red colored boxes are implemented in this current benchmark study. (B) A graphical representation of the SurvBenchmark design. The methods and evaluation metrics are summarized in a matrix with a flexible user-defined weights vector.

To comprehensively evaluate the strength and weakness of the survival analysis approaches, we select 20 representative methods from our extensive literature review and study their performance when applied to 16 diverse data sets. The performance of each method is measured against 11 metrics representing multiple aspects, including feasibility, predictability, stability, and computational efficiency. There are 3 key aspects of our comparison design SurvBenchmark, as depicted in Figure 1: (i) practical focus through applying the design to a broad range of data sets and by including a taxonomic methods system that evaluates multiple aspects, (ii) extensive comparison of methods from classical to state-of-the-art ML approaches, and (iii) comprehensive evaluation of the model performance with the utilization of a customizable weighting framework (Fig. 1B). To apply this in practice, the following steps are needed:

1. Provide a “weight vector” of length *q*, where each weight represents the strength for each of the *q* practical aspects. For example, if an urgent analysis is conducted, one may prefer a very high weight for computational time. On the other hand, prediction accuracy may be most important in a situation where computational constraints are of no concern.
2. According to the specific data modality, select the feasible methods (*m* in total) and obtain their rank (recorded in an *m*-by-*q* matrix) for the aspects considered in the “weight vector.”
3. Multiply the rank matrix and the “weight vector” to obtain the final selected method from this list of *m* scores (Supplementary Table S3).

### Practical consideration in assessing model performance

Many comparison studies define method performance solely in terms of method predictability, with only a few studies taking into account computational time. Often the feasibility of the method is not properly considered or discussed. Practically, it is paramount that a method can be applied to the data at hand, based on both the flexibility (data modality, sparsity) and computational requirement.

**Figure 2:**
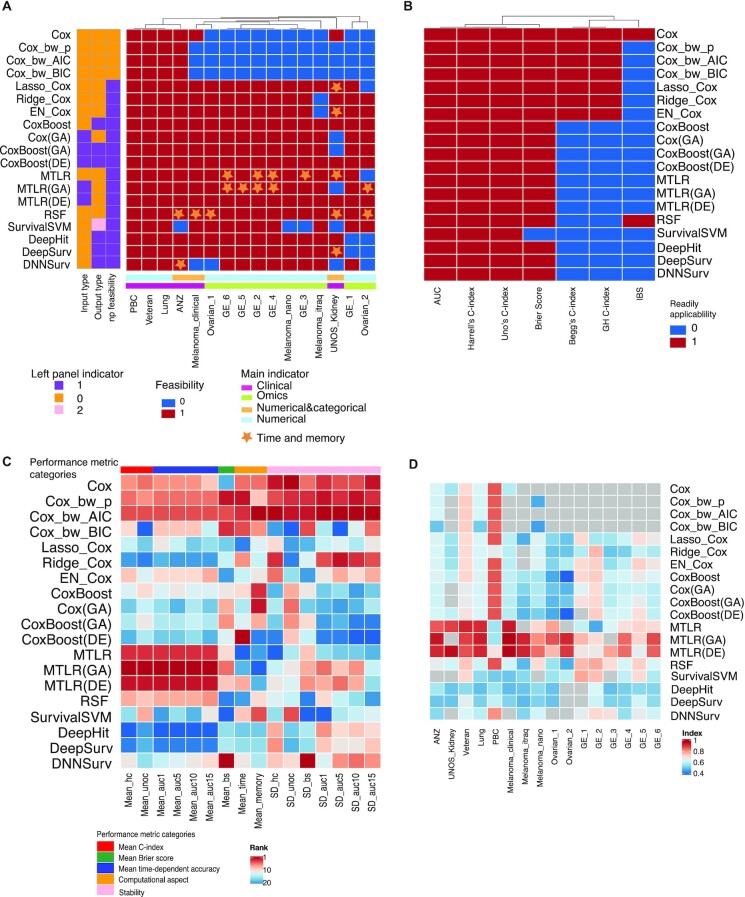
Summary heatmaps. (A) Summary for method flexibility and computational efficiency. Row: methods; Column: data sets; Dendrogram: similarity among data sets. Legend: (1) Left panel indicators 0, 1, and 2, where 0 represents “no,” 1 represents “yes,” and 2 represents “the other case” for the corresponding questions listed here. Is input type numeric only? Yes: numerical only. No: both numerical and categorical are OK. Is output type survival risk? Yes: survival risk. No: survival probability. Can the model handle *n* < *p* situation? Yes: it can. No: it cannot. The other case: output is the rank of survival risk. (2) Feasibility where red (1) means feasible and blue (0) means not feasible. (3) Main indicators including data set characteristics by different colors and stars represent the model is both memory and time-consuming. Similarity is defined using the Euclidean distance with feasibility indicator 0 and 1. (B) Prediction ability evaluation metric flexibility. Row: methods; Column: prediction ability evaluation metrics; Dendrogram: similarity among evaluation metrics; Legend: readily applicability where red (1) means readily applicable and blue (0) means not readily applicable. Similarity is defined using the Euclidean distance with feasibility indicator 0 and 1. (C) Rank heatmap for method overall performance. Row: methods; Column: performance metrics. Legend: (1) Rank: red to blue from 1 to 20, where 1 means the top rank. (2) Performance metric categories: 5 different categories representing all metrics used to evaluate method performances. (D) Harrell's C-index heatmap. Row: data sets; Column: methods; Legend: Harrell's C-index.

Given the diverse collection of data characteristics that is now available in the biomedical field, not all survival approaches are feasible to be applied to all data types, e.g., some classical Cox models (Fig. 2A, from column 1 to 10; a blue box indicates ‘method not feasible’) cannot handle large *p* (features) small *n* (samples) datasets (such as GE-1) which is a distinct feature of any molecular (omics) study. Advanced feature selection methods together with ML survival models such as CoxBoost(DE) can only take numerical data as the input (purple box for input type, where model characteristics are coded using 0, 1, and 2 with questions defined as below. Is input type numeric only? Yes: numerical only. No: both numerical and categorical are OK. Is output type survival risk? Yes: survival risk. No: survival probability. Can the model handle *n*< *p* situation? Yes: it can. No: it cannot. The other case: output is the rank of survival risk.).

Next, we look at the computational aspect, and we notice that DL-based methods are computationally inefficient as highlighted by the star icon (Fig. 2A). From the many rowwise stars, we observe that RSF (5 stars) and MTLR (5 stars) are not as computationally efficient as Cox-based approaches such as Lasso_Cox (1 star) and CoxBoost (0 stars).

Lastly, a summary tabulating the readily applicability associated with each of the evaluation metrics for prediction is provided in Figure 2B. The results highlight that Begg's C-index and GH C-index are applicable only for Cox methods (red indicates readily applicability), that the integrated Brier score can be calculated for the Cox model and RSF (red), and that the Brier score cannot be calculated for SurvivalSVM (blue).

### Performance evaluation from multiple perspectives: no “one size fits all”

To achieve a comprehensive overview of different survival approaches, we assess method performance from multiple perspectives across a large collection of data sets. Here, we color the methods according to their performances for all 3 broad categories: model predictability, model stability, and computational efficiency (Fig. 2C shows ranks of those methods where red means the best and blue the worst; similarly, Fig. 2D shows Harrell's C-index values with red referring to high values and blue to small values). We find that no method performs optimally across all 3 categories, and there are various trade-offs among the categories.

For model predictability, we use 7 different measures based on C-index, Brier score, and time-dependent AUC. Here, MTLR-based approaches perform evidently better than others, which is most apparent by looking at the performance results using C-index and time-dependent AUC. In order to further examine whether MTLR-based approaches have similar performance across all data sets, we show our examination on one specific criterion (the most popular Harrell's C-index; Mean_hc). In Figure 2D, we demonstrate that MTLR has optimal performance for all but 1 of the 6 clinical data sets with PBC having optimal performance for one of the clinical data sets. Variants of MTLR (MTLR(GA) and MTLR(DE)) outperformed MTLR when applied to any of the 10 omics data sets, suggesting the performance of the approaches depends on the type of data set.

For computational efficiency as measured by computational time and memory usage, the best-performing methods are classical Cox-based models and CoxBoost. In particular, Cox, Cox_bw_AIC, and Cox_bw_BIC are the top 3 performing methods for computational time (Fig. 2C). For model stability, we have 7 criteria, and they are based on calculating the SD of predictability metrics described above. Similar to the the computational efficiency performance, when using SD criteria, Cox, Cox_bw_AIC, and Cox_bw_BIC are also the top 3 performing methods in all but one criterion; the exception is the standard deviation of the Brier score (SD_bs), where DNNSurv ranks first, suggesting its ability to discriminate survival probabilities for different observations.

In conclusion, the above observations demonstrate that no method performs optimally for all those categories. In practice, we recommend first completing a feasibility check first to draw conclusions on time constraints and to heighten awareness of the data types actually present and then explicitly deciding on the focus of the research, for example, that model predictability is the top priority. Our analysis supports the use of MTLR and its variants for both omics and clinical data sets when survival prediction is the key priority, despite the fact that these approaches are inefficient [50]. However, Cox-based models are preferable when comparing the effect of variables, such as the treatment effect for clinical data sets, because of their efficiency and interpretability.

### Cox-based modern ML methods have similar prediction performance compared to classical Cox-based methods

**Figure 3:**
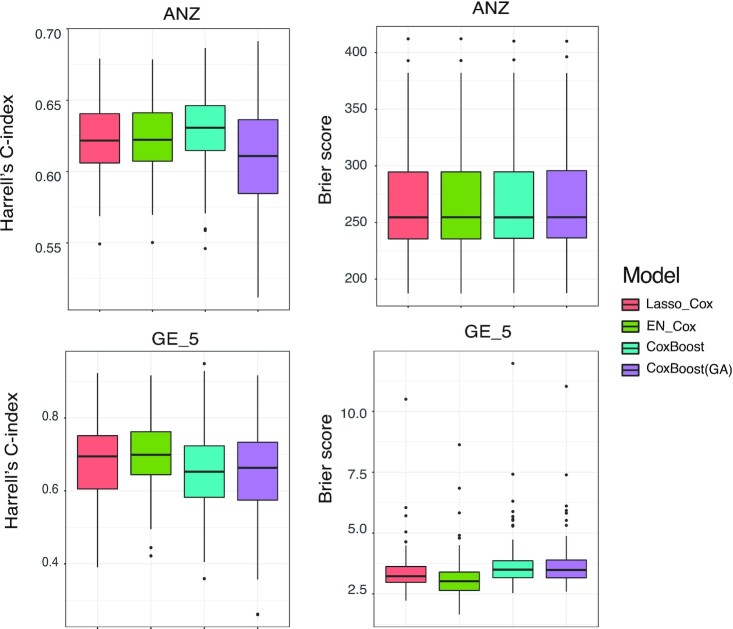
Prediction ability for Cox-based methods. Top left: Harrell's C-index on ANZ data. Top right: Brier score on ANZ data. Bottom left: Harrell's C-index on GE_5. Bottom right: Brier score on GE_5.

To understand the gain in model predictability from Cox-based modern ML methods (CoxBoost, Coxboost (GA)), we compare these models with classical Cox-based methods (Lasso_Cox, EN_Cox), which are used as a gold-standard method in many studies. Our results indicate that they have similar performance (Fig. 3) across a large collection of data sets. For example, in the ANZ data, which is a representative clinical data set, we observe similar model predictability measured by both Harrell's C-index and Brier score. For GE_5, a representative data set of omics with large *p* small *n* data characteristics, the same conclusion is drawn. This suggests the performance of modern ML methods in complex health and clinical data is not as clear-cut as in some other domains.

### Data-dependent model performance for different time

**Figure 4:**
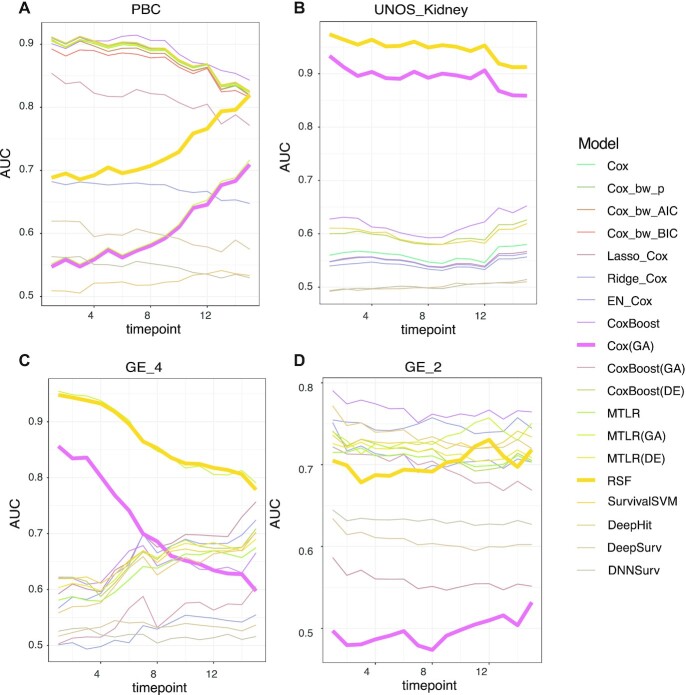
Time-dependent AUC curves. (A) PBC data. (B) UNOS_US data. (C) GE_4 data. (D) GE_2 data. Two selected models: Cox(GA) and RSF.

To study the model performance over time, we visualize this using the time-dependent AUC curves for all methods. Here we observe among 2 representative clinical data sets (PBC, UNOS_Kidney) and 2 omics data sets (GE_2, GE_4) in Figure 4, not all curves are parallel to each other, indicating that the behavior of model predictability for different time points is data dependent (see Supplementary Fig. S1 for further results).

We pick 2 representative models (Cox(GA) and RSF) to demonstrate this data-dependent model behavior. For UNOS_Kidney data and GE_2 data, the curves are approximately horizontal, which indicates the consistency of short-time, medium-time, and long-time model predictability. In contrast, for PBC data and GE_4 data, model predictability changes along those time points.

## Conclusions

This benchmark study comprehensively evaluated the relevance and usefulness of survival models in practice, where emphasis is on performance over diverse data sets. In our review, we assessed a broad variety of survival methods from classical CoxPH models to modern ML models. In this study, we did not survey the extensive tuning procedures for those survival approaches. The main reason is that, in practice, often the default hyperparameter sets are used. Therefore, we also decided to use the default sets in our study here. However, we note that applying targeted tuning methods for a particular data set may lead to different performances for the considered approaches. The findings of our systematic assessment will provide specific guidance for translational scientists and clinicians, as well as define areas of potential study in both survival methodology and benchmarking strategies.

In recent years, there is a clear shift in how survival data are analyzed, from modeling directly the hazard function to building models directly on survival functions. Conceptually, modeling hazard functions is a good way to identify key risk factors related to various patients’ risk levels. On the other hand, if our key criterion is to predict accurately survival, modeling survival probability directly improves predictability. Methods including MTLR, DNNSurv, and SurvivalSVM, which directly model the survival function, showed better performance in terms of model predictability, and this is consistent with what Yu et al. [27] have commented on when discussing the performance of their proposed MTLR method.

It is striking that MTLR shows remarkably high model predictability in our benchmark study. We now highlight technical advantages, disadvantages, and its applications. Numerous reasons could contribute to the better model prediction performance of the MTLR-based approaches. These include the 3 main reasons as discussed by Yu et al. [27]: direct modeling of the survival function, simultaneous building of multiple logistic regression models, and dynamic modeling. Interestingly, the majority of extended MTLR models since 2011 are based on neural networks as researchers extend the concept to account for nonlinearity in data sets [51]. To date, only a limited number of studies have applied MTLR in health using clinical data in HIV patients [52] or on large omics data sets to predict patient survival in breast and kidney cancers [53]. Given its outstanding model predictability observed for most of the data sets in our study, we believe there is opportunity to use MTLR more widely for survival risk modeling in health contexts.

Model predictability is one of the key metrics to assess survival studies, with Harrell's C-index being currently the most popular. As this kind of ranking-based concordance measurement is suitable to evaluate predicted outcomes with censored data, various concordance indices are developed using different methods to handle censoring such as Uno's C-index using IPCW. Besides concordance indices, other predictability metrics such as the time-dependent AUC, which applies a similar idea as the AUC in binary classification but divides the whole time interval into multiple time points, are also adopted in some survival studies [54]. Given that model predictability could be measured by multiple types of indices, we suggest that hybrid evaluation metrics should be applied in practice to provide relatively comprehensive assessments for the fitted model.

While many survival approaches are applicable to both clinical and omics data, there are a number of recently developed approaches that are specifically tailored for high-dimensional omics data, such as CoxBoost. The rationale behind developing data-specific methods is to better capture the distinct data characteristics in either the clinical or omics studies. Clinical data usually include mixed-modality variables and large sample sizes but have large *n* (observations) and small *p* (features). In contrast, omics data naturally come with a large collection of molecular features and with small *n* but their data type is homogeneous. When it comes to various real-world data sets, performances are also affected by many other aspects besides data type (clinical, omics) such as data modality, and therefore, it is challenging to directly examine whether those tailored methods indeed improve the performance. Further examination of the aspects that affect model predictability can be found in Supplementary Table S2.

Deep learning–based methods failed for some data sets on some cross-validation runs. Taking the method DNNSurv as an example, among all 100 runs, DNNSurv had a 100% completion rate for 5 out of the 12 applicable data sets (Supplementary Fig. S2) only. For the remaining 7 data sets, completion rate was around 80% and as low as 63% for the Melanoma_itraq data. This instability is likely due to tuning parameter sensitivity when the sample size is small [55]. All failed iterations are not recorded when generating the results.

## Additional Files

**Supplementary Fig. S1:** Data dependent performance for short and long time prediction.

**Supplementary Fig. S2:** Method reproducibility.

**Supplementary Fig. S3:** Uno’s C-index boxplots.

**Supplementary Fig. S4:** Brier Score boxplots.

**Supplementary Table S1:** Different evaluation criteria for assessing the performance models.

**Supplementary Table S2:** Examination of potential aspects that affect the model predictability using Harrell’s C-index.

**Supplementary Table S3**: Ranking matrix.

giac071_GIGA-D-22-00036_Original_Submission

giac071_GIGA-D-22-00036_Revision_1

giac071_Response_to_Reviewer_Comments_Original_Submission

giac071_Reviewer_1_Report_Original_Submission Xiangqian Guo -- 3/13/2022 Reviewed

giac071_Reviewer_2_Report_Original_Submission Moritz Herrmann -- 4/13/2022 Reviewed

giac071_Supplemental_Files

## List of Abbreviations

KM: Kaplan-Meier; CoxPH: Cox proportional hazards model; SVM: support vector machine; RSF: random survival forest; ML: machine learning; TCGA: The Cancer Genome Atlas; MTLR: multi-task logistic regression; DL: deep learning; NN: neural networks; GA: genetic algorithm; DE: differential expression analysis; IPCW: inverse probability of censoring weight; MSE: mean squared error; IBS: integrated Brier score; ROC: receiver operating characteristic; AUC: area under the curve; SD: standard deviation.

## Data Availability

For the ANZDATA, data request can be made through the ANDATA registry, and access to the data source will require HREC approvals. UNOS_kidney data can be requested from [56]. Codes for running those methods and evaluation measurements for an example data set are available at [57]. All supporting data and materials are available in the *GigaScience* GigaDB database [58].

Availability of supporting source code and requirements.

Project name: SurvBenchmark Project

Home page: https://github.com/SydneyBioX/SurvBenchmark_package

Operating system(s): Platform independent

Programming language: R

Other requirements: R studio

License: Apache 2.0 RRID:SCR_022503

## Authors’ Contributions

JYHY and SM conceived, designed, and funded the study with guidance from GW and GM. GM and GW provided access to in-house data and jointly developed the problem formulation of the study with JYHY and SM. YZ developed the benchmarking design and, implemented all the models in R and the evaluation design with guidance from JYHY and SM. YZ, JYHY, and SM wrote the manuscript and all authors read and approved the final version of the manuscript.

## Funding

The following sources of funding for each author, and for the manuscript preparation, are gratefully acknowledged: Australian Research Council Discovery Project grant (DP210100521) to SM, National Health and Medical Research Council's CRE (APP1135285) to JYHY and GJM, AIR@innoHK programme of the Innovation and Technology Commission of Hong Kong to JYHY, and Research Training Program Tuition Fee Offset and Stipend Scholarship and the Dean's International Postgraduate Research Scholarship (DIPRS) to YZ. The funding source had no role in the study design; in the collection, analysis, and interpretation of data; in the writing of the manuscript; and in the decision to submit the manuscript for publication.

## Competing Interests

The authors declare that they have no competing interests.
