# Supplementary material for: SurvBenchmark: comprehensive benchmarking study of survival analysis methods using both omics data and clinical data
Source: Gigascience. 2022 Jul 30;11:giac071. doi: 10.1093/gigascience/giac071 (PMC9338425; doi:10.1093/gigascience/giac071)
Supplement: giac071_GIGA-D-22-00036_Original_Submission [file giac071_giga-d-22-00036_original_submission.pdf]

GigaScience

SurvBenchmark: comprehensive benchmarking study of survival analysis methods using both omics data and clinical data

--Manuscript Draft--

**Manuscript Number:**	GIGA-D-22-00036
**Full Title:**	SurvBenchmark: comprehensive benchmarking study of survival analysis methods using both omics data and clinical data
**Article Type:**	Research
**Funding Information:**	Dean's International Postgraduate Research Scholarship (DIPRS) — Ms. Yunwei Zhang<br>Australian Research Council Discovery Project grant (DP170100654) — Dr. Samuel Muller, Dr. Jean Y.H. Yang<br>Australian Research Council Discovery Project grant (DP210100521) — Dr. Samuel Muller<br>AIR@innoHK programme of the Innovation and Technology Commission of Hong Kong — Dr. Jean Y.H. Yang
**Abstract:**	Survival analysis is a branch of statistics that deals with both, the tracking of time and of the survival status simultaneously as the dependent response. Current comparisons of survival model performance mostly center on clinical data with classic statistical survival models, with prediction accuracy often serving as the sole metric of model performance. Moreover, survival analysis approaches for censored omics data have not been thoroughly investigated. The common approach is to binarise the survival time and perform a classification analysis.<br>Here, we develop a benchmarking design, SurvBenchmark, that evaluates a diverse collection of survival models for both clinical and omics datasets. SurvBenchmark not only focuses on classical approaches such as the Cox model, but it also evaluates state-of-art machine learning survival models. All approaches were assessed using multiple performance metrics, these include model predictability, stability, flexibility and computational issues. Our systematic comparison design with 320 comparisons (20 methods over 16 datasets) shows that the performances of survival models vary in practice over real-world datasets and over the choice of the evaluation metric. In particular, we highlight that using multiple performance metrics is critical in providing a balanced assessment of various models. The results in our study will provide practical guidelines for translational scientists and clinicians, as well as define possible areas of investigation in both survival technique and benchmarking strategies.
**Corresponding Author:**	Jean Yee Hwa Yang, PhD<br>The University of Sydney<br>Sydney, NSW AUSTRALIA
**Corresponding Author Secondary Information:**	
**Corresponding Author's Institution:**	The University of Sydney
**Corresponding Author's Secondary Institution:**	
**First Author:**	Yunwei Zhang
**First Author Secondary Information:**	
**Order of Authors:**	Yunwei Zhang<br>Germaine Wong<br>Graham Mann<br>Samuel Muller<br>Jean Y.H. Yang

Powered by Editorial Manager® and ProduXion Manager® from Aries Systems Corporation

Order of Authors Secondary Information:	
Additional Information:	
Question	Response
Are you submitting this manuscript to a special series or article collection?	No
**Experimental design and statistics**<br><br>Full details of the experimental design and statistical methods used should be given in the Methods section, as detailed in our Minimum Standards Reporting Checklist. Information essential to interpreting the data presented should be made available in the figure legends.<br><br>Have you included all the information requested in your manuscript?	Yes
**Resources**<br><br>A description of all resources used, including antibodies, cell lines, animals and software tools, with enough information to allow them to be uniquely identified, should be included in the Methods section. Authors are strongly encouraged to cite Research Resource Identifiers (RRIDs) for antibodies, model organisms and tools, where possible.<br><br>Have you included the information requested as detailed in our Minimum Standards Reporting Checklist?	Yes
**Availability of data and materials**<br><br>All datasets and code on which the conclusions of the paper rely must be either included in your submission or deposited in publicly available repositories (where available and ethically appropriate), referencing such data using a unique identifier in the references and in the "Availability of Data and Materials" section of your manuscript.	Yes

Have you have met the above requirement as detailed in our Minimum Standards Reporting Checklist?	

responses to reviewers
Click here to access/download;Manuscript;survbenchmark-response to reviewer.docx

THE UNIVERSITY OF SYDNEY

Hongling Zhou
Associate Editor
GigaScience

18 Feb 2021

Dear Dr. Zhou

Thank you for inviting us to submit a revised manuscript "SurvBenchmark: comprehensive benchmarking study of survival analysis methods using both omics data and clinical data" by Zhang et al as a new submission in GigaScience. We thank you for your initial consideration of our manuscript numbered GIGA-D-21-00310 and thank the reviewers for their constructive feedback. Based on these, we have addressed all concerns raised and made considerable improvements to the manuscript. The summary of the significant modifications are:

1. **Additional analytical experiments for the comparison study.** We have added a number of new experiments including:

- Failure iteration examinations for all datasets with all methods. We provide a full summary in our supplementary file.
- Further examined how aspects related to data characteristics could potentially affect model predictability using a linear regression model.
- Provided a ranking matrix as a potential guidance for method selection with practical considerations.

2. **Fair comparison in the study design:** We have changed all experiments to using the default set of hyperparameters for all methods to ensure fair comparison among them. We have revised all our methods and evaluations. We observe that this change to the default hyperparameter values does not change the results, when compared to the original results.

We believe these changes have improved the analysis in a convincing manner and have substantially improved the manuscript. Please find following this letter, our point-by-point response to the reviewer's comments. We thank you for your time and look forward to your decision on our revised manuscript.

Yours sincerely,

Jean Yee Hwa Yang (on behalf of all authors)

Response to editor and reviewer comments

We thank all the reviewers for their valuable comments. We have addressed all comments raised by each of the reviewers as per the point-by-point response below.

Reviewer reports:

Reviewer #1: SurvBenchmark: comprehensive benchmarking study of survival analysis methods using both omics data and clinical data.

Authors compared many survival analysis methods and created a benchmarking framework called as SurvBenchmark. This is one of the extensive study using survival analysis and will be useful for translational community. I have few suggestions to improve the quality of the manuscript.

We thank the reviewer for this positive assessment and have addressed all suggestions as follows:

1. Figure 1: LASSO, EN and Ridge are regularization methods. So, I would suggest including a new classification category as "regularization" or "penalization methods" and take out those from non-parametric models. Obviously this also need to be included accordingly in the methodology section and discussions

Response:
We agree that Figure 1, Lasso_Cox, Ridge_Cox, etc are regularization methods. We have grouped them into semi-parametric models, which also include the Cox-based models in survival analysis to avoid confusion with regularization methods that belonged to modern machine learning models (e.g. MTLR, which also has a regularization term in its loss function).

We have now added the following sentence in Section 2.1 in line 122 of the revised manuscript to clarify this.

"Notice that the various types of regularization terms can also appear in the loss function of modern ML methods which we introduce in Section 2.2."

2. Data sets: please provide a table with six clinical and ten omics data sets with number of samples, features and reference link.

Response:
To clarify, these details are provided in the Supplementary Table 1 in our original submission. We have now moved this into the revised manuscript as Table 1.

3. Discussion section: How the choice of the method need to be chosen? What criteria need to be used? I understand one does not fit all but some sort of clear guidance will be very useful.

Response:
We have addressed this as follows:

1. **Choice of the method and criteria**: Our aim was to provide guidance for the trade-off among multiple selection criteria and between the clinical and omics datasets, but not the identification of a single optimal methods for both clinical and omics datasets
2. **Providing some sort of clear guidance**: We agree that although there is no single method that fits all datasets, depending on the research aims and practical limitations, we have the following recommendations. In practice, if the aim is to obtain accurate predictions and when computational

time and memory limits are not strictly restricted, we recommend MTLR and its variants for both clinical and omics datasets. We conclude this from the feasibility results shown in Figure 2a, where there is always one MTLR based method that can be applied. From Figure 2c, when looking at the evaluation with multiple metrics, MTLR achieves good predictability for long and short time outcomes. However, if the objective of the study was to evaluate the effect on survival for a particular variable or when comparing two or more groups with different demographics in a clinical dataset, we recommend that classical Cox-based methods should be applied, because these are more interpretable. Further, based on Result 4.4, we see that when compared with Cox-based modern ML methods, the classical Cox-based methods have comparable prediction performance.

We have changed section 4.3 lines 278-280 in the original manuscript to the following.

"In conclusion, the above observations demonstrate that no method performs optimally for all those categories. In practice, we recommend first completing a feasibility check first to draw conclusions on time constraints and to heighten awareness of the data types actually present, and then explicitly deciding on the focus of the research, for example that model predictability is the top priority. Our analysis supports the use of MTLR and its variants for both omics and clinical datasets when survival prediction is the key priority, despite the fact that these approaches are inefficient [50]. However, Cox-based models are preferable when comparing the effect of variables, such as the treatment effect for clinical datasets, because of their efficiency and interpretability."

Also sample size related aspects need to be more discussed. In the omics research number of samples are really limited and deep learning based survival analysis is not feasible as authored mentioned in the line number 328-331. So, question come, when we should used deep learning based methods and when we should not.

Response:
Thank you for this comment. For the study of omics datasets, our goal is to explore how well the various methods perform on these datasets using a variety of evaluation metrics (original manuscript line 213), rather than focusing on the impact of a single aspect on model performance. We broadly classify these series of metrics into three key aspects (original manuscript line 213).

While sample size is an important data characteristic that affects the model predictability, it is not the only important data characteristic. To provide a better discussion we perform a new analysis to examine how various data characteristics impact model predictability, we perform a multiple linear regression between

- the performance metrics (y-variable) represented by Harrell's C-index metric, and
- a set of x variables. These are "number of observations", "number of features", "censoring rate", "ratio: n/p", "number of numerical variables", "number of categorical variables", and "ratio: nc/nn".

The results from the deep learning based method Deephit indicate that the following four variables have a significant association, they are "number of observations", "number of numerical variables", "number of categorical variables" and "ratio: nc/nn". The regression model has an R square value of 0.73. Therefore, we can see that deep learning based methods should be applied when we have a relatively large number of samples or variables.
We have added in section 5 starting in line 325 of the original manuscript the following changes:

"When it comes to various real-world datasets, performance is also affected by many other aspects besides data type (e.g. clinical or omics) such as data modality and therefore, it is challenging to directly

examine whether those tailored methods indeed improve the performance. Further examination of the aspects that affect model predictability can be found in Supp Table 2. "

Reviewer #2: Summary:

The authors conducted a benchmark study of survival prediction methods. The design of the study is reasonable in principle. The authors base their study on a comprehensive set of methods and performance evaluation criteria. In addition to standard statistical methods such as the CoxPH model and its variants, several machine learning methods including deep learning methods were used. In particular, the intention to conduct a benchmark study based on a large, diverse set of datasets is welcome. There is indeed a need for general, large-scale survival prediction benchmark studies. However, I have serious concerns about the quality of the study, and there are several points that need clarification and/or improvement.

We thank the reviewer for the constructive comments and recognition of the significance of this work. We have clarified all points raised below. We have also conducted additional experiments. We believe that thanks to these comments, the revised version of the work is substantially improved.

Major issues:

1. The method comparison does not seem fair
As far as I can tell from the description of the methods, the method comparison is not fair and/or not informative. In particular, given the information provided in Supp-Table-3 and the code provided in the Github repository, hyperparameter tuning has not been conducted for some methods. For example, Supp-Table-3 indicates that the parameters 'stepnumber' and 'penaltynumber' of the CoxBoost method are set to 10 and 100, respectively. Similarly, only two versions of RSF with fixed ntree (100 and 1000) and mtry (10, 20) values are used. Also, the deep learning methods appear not to be extensively tuned.
On the other hand, telling from the code, methods such as the Cox model variants (implemented via glmnet) and MTLR have been tuned at least a little.
Please clearly explain in detail, how the hyperparameters have been specified respectively how hyperparameter tuning has been conducted for the different methods? If, in fact, not all methods have been tuned, this is a serious issue and the experiments need to be rerun under a sound and fair tuning regime.

Response:
We thank the reviewer for this comment. We are aware of and appreciate the importance of hyperparameter tuning for deep learning and machine learning based algorithms, where hyperparameter tuning might deliver improved performance for a specific dataset. We are also aware that there are a lot of different tuning methods that have been proposed and each have pros and cons when applied with different types of methods or datasets. In a recent review (Yu, T., & Zhu, H. (2020). Hyper-parameter optimization: A review of algorithms and applications. arXiv preprint arXiv:2003.05689), the authors discussed how different tuning methods differ on both methods and datasets. For two deep learning based survival models: DNNSurv and DeepHit, the recommended tuning method in DNNSurv is the grid search method while the random search method is used in the DeepHit paper to benchmark its performance.
We have provided additional results in the supplementary that address the impact of optimal tuning. However, as this is not the focus of our study, we prefer to use the default choice of hyperparameters for each of the considered methods as we believe this to be in balance a 'fair' comparison among the considered approaches, and is in line with other work that does not focus on

the optimal tuning for every method. We give some additional justification below to why we prefer to use the default parameters after highlighting what we have changed in the revised manuscript.

The impact of using the default hyperparameters can be observed by assessing their performance on different datasets. For clarity, we have provided the specific values used in our updated Table 2 and, to be consistent, have updated all our figures and tables using default values of all methods.

We have updated line 227 in the original manuscript to the following:

“In this benchmark study, hyperparameter sets used in these methods are chosen to be the default set.“

We now provide additional justification to why we prefer to use default parameters in the main manuscript.

In practice, one optimal tuning method for one approach on a certain dataset may not be optimal for another approach on another dataset and determining how to choose the appropriate tuning method is often challenging. To highlight this further we have done some additional work which we show in this response letter but which we do not add to the revised manuscript as follows:

We apply two popular tuning methods: grid search and random search on seven models for two example datasets (GE-5 and Veteran). We summarize the results in the figure below. We can see from that figure that for some models, one tuning method may outperform the other on both datasets, whereas for some models, the optimal tuning method is data-dependent. Specifically, for MTLR(GA), tuning_method 1 outperforms tuning_method 2 on both datasets. For MTLR, tuning method_1 outperforms tuning_method 2 for Veteran data but the opposite for the GE-5 data. For the model Ridge_Cox, those two tuning methods have similar performance on the Veteran data but tuning_method 2 outperforms tuning_method 1 on GE-5. We also notice that there may be no difference observed for some models such as RSF and SurvivalSVM, i.e., their performances are similar. Therefore, different tuning methods may bias the model performances.

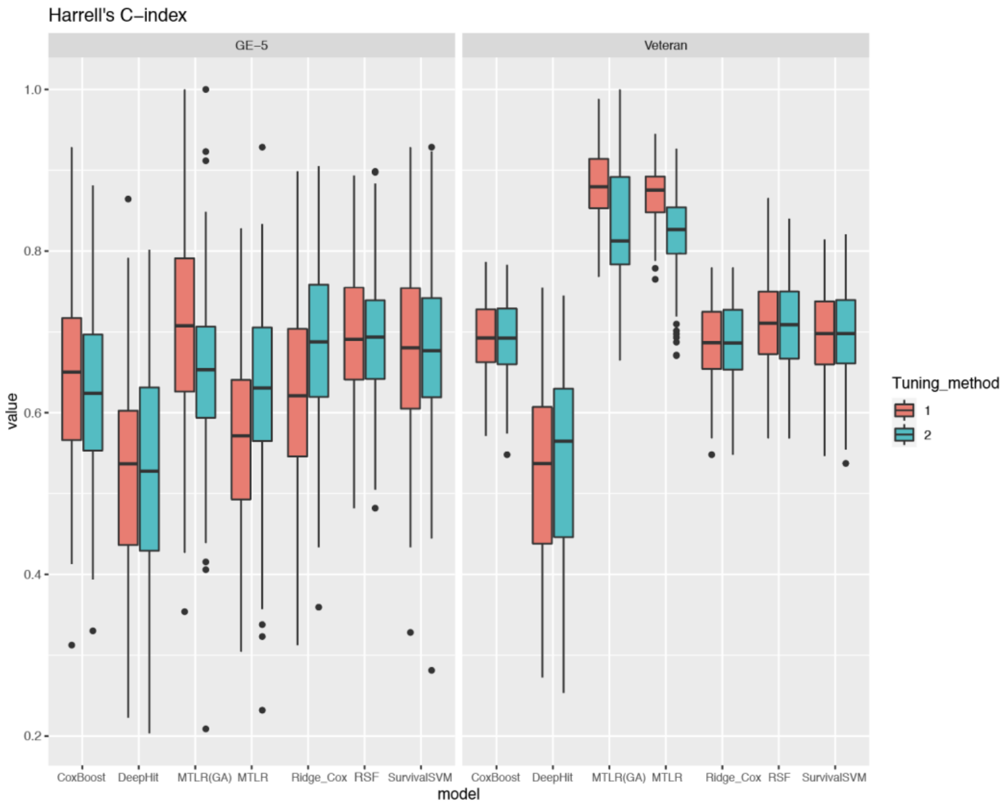

In summary, including all tuning methods with all possible tuning parameters for all datasets and approaches would result in too vast a number of comparisons in our analysis. This is beyond the scope of this study. In addition, we want to look at how models are used in real-world circumstances when "extensive tuning" is less likely to occur. Therefore, we chose to apply default parameters with the focus on the impact of diverse datasets.

2. Description of the study design

Related to the first point, the description of the study design needs to be improved in general as it does not allow to assess the conducted experiments in detail. A few examples, which require clarification:

- as already mentioned, the method configurations and implementations are not described sufficiently. It is unclear how exactly the hyperparameter settings have been obtained, how tuning has been applied and why only for some methods

Response:

Thank you for this comment, please see our response to your previous comment and the sets of parameters used are now detailed in Supplementary Data (column "Parameters (default)" gives details of the default parameter sets and column "Parameters tuning (grid search)", "Parameters tuning (random search)" give the details under tuning).

- concerning the methods Cox(GA), MTLR(GA), COXBOOST(GA), MTLR(DE), COXBOOST(DE): have the feature selection approaches been applied on the complete datasets or only on the training sets

Response:

The feature selection approaches are applied within the 5-fold cross validation and only on the training part of the data to ensure no data leakage. To clarify this, we have now added the following sentence in Section 3.2 (line 230 in the original manuscript) to clarify this detail.

"For each run, the whole data is split into a training dataset (80%) and a testing dataset (20%). Each method is then trained using the training dataset and values of evaluation metrics are calculated using the testing dataset. For methods with a feature selection step, a nested feature selection step is applied on those training folds within each 5-fold cross validation procedure. "

- Supp-Table-3 lists two implementations of the Lasso, Ridge and Elastic Net Cox methods (via penalized and glmnet); yet, Figure 2 in the main manuscript only lists one version. Which implementations have been used and are reported in Figure 2?

Response:
The "penalized" package cannot handle situations where the number of variables (p) is larger than sample size (n). Therefore, for datasets with p < n, the "penalized" package is used and for datasets with n < p, the "glmnet" package is used.

This information is included in the Supplementary Data in the last column "N<p feasibility": with "yes" meaning feasible, and "no" meaning not applicable.

- l. 221: it is stated that "the raw Brier score" has been calculated. At which time point(s) and why at this/these time point(s)?

Response:
Thank you for this comment. The raw Brier score was calculated by first calculating for all time points (i.e. all the event times in the datasets) the Brier score and then obtaining the raw Brier score through taking the sum of all these Brier scores.

We have now made this clearer in the manuscript by adding "*As for the Brier score, we calculated the raw Brier score and the IBS. The raw Brier score is calculated by taking the sum of all Brier scores for all event times in the dataset.*" in original manuscript line 221, section 3.2.

- Supp-Table-2: it is stated that "some methods are not fully successful for all datasets", but only DNNSurv is further examined. Is it just DNNSurv or are there other methods that have failed in some iterations?

Response:
There are other methods (DeepHit, DeepSurv, bw_CoxAIC, etc) that have failed in some iterations and we have used DnnSurv as an illustrating example. Among those other methods, this issue can only be observed on one dataset. We have now added new Supp figures to illustrate it. (Supp Figure 2)

In our manuscript, we have revised Section 5 line 328:

"Deep learning based methods failed for some datasets on some of the cross-validation runs. Taking the method DNNSurv as an example, among all 100 runs, DNNSurv had a 100% completion rate for 5 out of the 12 applicable datasets (Supp Figure 2_Supplementary Material) only."

Moreover, what has been done about the failing iterations? Have the missing values be imputed? Are the failing iterations ignored?

Response:
Thank you for this comment. We confirm that failing iterations are not used to generate the results and that there are no missing values.

We have now made this explicit in the new manuscript in line 461 by adding:

"All failed iterations are not recorded when generating the results."

I recommend that section 3 be comprehensively revised and expanded, in particular including the methods implementations, how hyperparameters are obtained/tuning has been conducted, aggregation of performance results, handling of failing iterations. Moreover, I suggest to provide summary tables of the methods and datasets in the main manuscript and not in the supplement.

Response:

We have comprehensively revised and expanded Section 3 and specific expansions are stated in the above points.

We have moved the summary table of the datasets (Table 1) and the summary table of methods (Table 2) into the main manuscript. We also provide a detailed version of Table 2 as the Supplementary Data which gives details of each method and also the tuning methods (Supplementary Data: the column "Parameters (default)" gives details of the default parameter sets and the column "Parameters tuning (grid search)", "Parameters tuning (random search)" give the details under tuning).

3. Reliability of the presented results

In other studies [BRSB20, SCS+20, HPH+20] differences in (mean) model prediction performance have been reported to be small (while variation over datasets can be large). This can also be seen in Figure 3 of the main manuscript.Please include more analyses on the variability of prediction performances and also include a comparison to a baseline method such as the Kaplan-Meier estimate.

Response:

Our analysis on the variability of prediction performances is shown in Figure 2c, through the category color pink, which we named "Stability": this includes the calculation of the standard deviations of the C-indices and Brier scores and time-dependent AUC. This was stated in the original manuscript in method section 3.2 Evaluation metrics (iv) line 224 (which is now line 294 in the revised manuscript).

We agree that the Kaplan-Meier estimate has been used in many studies as a baseline method. Here in our study, we use the Cox model as our baseline method because we have more than one and not a single variable. Specifically, we have a multivariate analysis comparison using those models on different datasets with more than one variable and with both, numerical variables and categorical variables. Given that the Cox model can easily handle more than one variable and different variable types, we believe it is an appropriate baseline method for this benchmark study.

Most importantly, if some methods have been tuned while others have not, the reported results are not reliable. For example, the untuned methods are likely to be ill-specified for the given datasets and thus may yield sub-optimal prediction performances. Moreover, if internal hyperparameter tuning is conducted for some methods, for example via cv.glmnet for the Cox model variants, and not for others, the computation times are also not comparable.

Response:

Please see our response to point 1. We have ensured a fair comparison by (1) implementing all the default parameter sets and (2) for a selected dataset, comparing between methods after tuning. Under the comparison using only default parameters, our results are similar compared to the original results.

4. Clarity of language, structure and scope

I believe that the quality of the written English is not up to the standard of a scientific publication and consider language editing necessary (yet, it has to be taken into account that I am not a native speaker). Unlike related studies [BWSR21, SCS+20, e.g.], the paper lacks clarity and/or coherence.
Although clarity and coherence can be improved with language editing, there are also imprecise descriptions in section 2 that may additionally require editing from a technical perspective. For example:

Response:

We have now improved the clarity of language, structure and scope in the revised manuscript. We have an author who is a native English speaker and who has particularly taken care in revising the wording and we believe the revised version is of satisfactory standard for a publication in GigaScience.

- l. 76 - 78: The way censoring is described is not coherent, e.g.: "the class label '0' (referring to a 'no-event') does not mean an event class labelled as '0'". Furthermore, it is not true that "the event-outcome is 'unknown'". The event is known, but the exact event time is not observed for censored observations.

Response:

Thank you for this comment. The class label ‘0’ refers to censored observations as their exact event time is not observed. We have now reworded the sentence to:
“the class label ‘0’ means an observation is censored as its exact event time is not observed.”

- The authors aim to provide a comprehensive benchmarking study of survival analysis methods. However, they do not, for example, provide significance tests for performance differences nor critical differences plots (it should be noted that the number of datasets included may not provide enough power to do so). This is in stark contrast to the work of Sonabend [Son21].

Response:

Thank you for this comment. We are aware of using significance tests to demonstrate differences in model performances. However, there are discussions on whether statistical significance tests should be used and which one should be used. Dietterich [D98] shows concern of using the paired Student’s t-test to compare model predictability because of the violation of the test assumption. Also, in terms of comparing multiple datasets with multiple models, Demsar [D06] points out the inappropriateness for using ANOVA and they proposed the Friedman test with the corresponding post-hoc tests.

In the survival analysis literature, there have been attempts to compare the difference of two C indices [KCPG15]. However, a simulation study [HZS17] points out that “the difference of two correlated Harrell’s C statistics may not converge to zero under the null hypothesis, thus may lead to severely inflated type I error for the proposed test.” and therefore, they conclude that currently, there is no available practical strategy to verify the conditions required for the proposed test in [KCPG15].

From that literature, it is a non-trivial statistical problem to determine an appropriate statistical test to evaluate two C-indexes. Thus, focussing mostly on p-values could detract readers from the main conclusions in our study.

That is why here, we focus on performance differences rather than statistically significant differences, particularly informing on the magnitude of the difference. Thus, we decided to use graphics that visualize to what extent methods differ.

We illustrate the three-way comparison using heatmaps and boxplots.

Figure 2c shows the model evaluation summarized for all datasets in a heatmap by ranking those models. We considered (1) model predictability evaluated by C-index, Brier score and time-dependent AUC; (2) model predictability variation evaluated by standard deviations of those predictability metrics; (3) computational time; (4) memory consuming, and a top rank means good model predictability, less time and memory required, small standard deviation.

Figure 2d is also a heatmap showing the model predictability measure by C-index for each specific dataset, where the color bar indicates the real C-index for a model-dataset combination, highlighting the difference in "mean".

In addition to heatmaps, we have also provided the boxplots to directly illustrate the spread and the median value on representative datasets across different methods about model performances measured by the C-index and Brier score.

Whilst the critical difference plot also presents comparisons for different models' predictability, it does not show the actual ranks of each model directly in the plot. Instead, it plots the test statistics which are calculated based on ranks. In our opinion, this is not as intuitive as using heatmaps that show the ranks directly. In addition, our boxplots offer the variability of the evaluation metrics as a complement to the average ranks.

Ref:

[D98] Dietterich TG. Approximate Statistical Tests for Comparing Supervised Classification Learning Algorithms. Neural Comput. 1998;10:1895–923.
[D06] Demsar, Janez. Statistical Comparisons of Classifiers over Multiple Data Sets. Journal of Machine Learning Research. 2006; 7. 1-30.
[KCPG15] Kang L, Chen W, Petrick NA, Gallas BD. Comparing two correlated C indices with right-censored survival outcome: a one- shot nonparametric approach. Statistics in Medicine. 2015. p. 685–703. Available from: http://dx.doi.org/10.1002/sim.6370
[HZS17] Han X, Zhang Y, Shao Y. On comparing 2 correlated C indices with censored survival data. Statistics in Medicine. 2017. p. 4041–9. Available from: http://dx.doi.org/10.1002/sim.7414

I suggest revising section 2 using more precise terminology and clearly describing the scope of the study, e.g., what type of censoring is being studied, whether time-dependent variable and effects are of interest, etc. I think this is very important, especially since the authors aim to provide "practical guidelines for translational scientists and clinicians" (l. 32) who may not be familiar with the specifics of survival analysis.

Response:

Thank you for this comment. We now use more precise terminology and have made the following changes:

-line 56:

"There are many studies in the literature that give a good overview on right-censored data without time-dependent covariates, for example [5]."

-line 88:

"To this end, we develop a benchmarking design SurvBenchmark that considers multiple aspects with several evaluation metrics on a large collection of real-world health and biomedical datasets to guide right-censored data survival method selection and new method development."

Minor issues

- l. 43: Include references for specific examples

Response:
We have now added three additional references from different disciplines (Engineering, Economics and Tourism) as follows:

- Engineering: Esmalian A, Dong S, Mostafavi A. Susceptibility curves for humans: Empirical survival models for determining household-level disturbances from hazards-induced infrastructure service disruptions. Sustainable Cities and Society. 2021. p. 102694.
- Economics: Ancarani A, Di Mauro C, Fratocchi L, Orzes G, Sartor M. Prior to reshoring: A duration analysis of foreign manufacturing ventures. International Journal of Production Economics. 2015. p. 141–55.
- Tourism: Thrane C. Analyzing tourists' length of stay at destinations with survival models: A constructive critique based on a case study. Tourism Management. 2012. p. 126–32.

- l. 60: The cited reference probably is not correct
Response:
This is a typo and we have now changed from [6] to [5].

- l. 266: "MTLR-based approaches perform significantly better". Was a statistical test performed to determine significant differences in performance? If yes, indicate which test was performed. If not, do not use the term "significant" as this may be misunderstood as statistical significance.
Response:
In reflection, we agree that "significantly better" could lead some readers to interpret this as "statistically significantly better". *We have now changed this sentence to "MTLR-based approaches perform evidently better".*

- Briefly explain what the difference is between data sets GE1 to GE6.
Response:
The six data sets (GE1 to GE6) are completely different gene expression datasets. We have named them GE1 to GE6 for ease of reading of the figures, as this ensures labels are short.
Full details of those datasets is given in the paper referenced in Table 1.

We have now added *"Another six gene expression datasets are available online from work by Yang and colleagues [45], We have named them GE1 to GE6 for ease of reading of the figures. "* in line 264 to make this more explicit in the paper.

- It has been shown that omics data alone may not be very useful [VDBSB19]. Please explain why only omics variables are used for the respective datasets.
Response:
Thank you for this comment. The study [VDBSB19] focuses on integrating omics data and clinical data to examine the added value of omics data in terms of model predictability, which is not what we study in this work. Further, as only two datasets are considered in the study [VDBSB19], we believe that it would be premature to conclude that studies on omics datasets alone have little value. There are also studies such as [TWW21] that focus on the integration of multiple modalities of "omics" data for survival model predictability, which uses omics data only.

We highlight that examining the added value of omics data in terms of model predictability in [VDBSB19] is different from our aim. We do not aim to compare for a specific disease, whether omics data provides stronger signal than clinical data. Actually, the study in [DSB14] points out that "Firstly, the combined use of clinical and omics predictors makes sense only when the latter contain added predictive value and do not simply provide similar information as the clinical predictors.". As we would like to see how model performances vary in high-dimensional "omics" data (larger number of features than the number of observations), we prefer to use omics data only to not confuse such performance differences with the differences in the clinical part of mixed data. In practice of course, when corresponding clinical information is available, adding these as features when applying the methods would be natural.

Ref:
[DSB14] De Bin, R., Sauerbrei, W., & Boulesteix, A. L. Investigating the prediction ability of survival models based on both clinical and omics data: two case studies. Statistics in medicine. 2014. 33(30). 5310-5329.
[TWW21] Tong L, Wu H, Wang MD. Integrating multi-omics data by learning modality invariant representations for improved prediction of overall survival of cancer. Methods. 2021;189:74–85.

- Figure 1: Consider changing the caption to 'An overview of survival methods used in this study' as there are survival methods that are not covered. Moreover, consider referencing Wang et al [WLR19] as Figure 1a resembles Figure 3 presented therein.

Response:

Thank you for this comment.

We have now changed the caption of Figure 1a to

"An overview of survival methods used in this study".

We have now referenced the article Wang et al [WLR19] in the Figure caption as

"We broadly classify current models into two categories; classical statistical models (top group) and modern machine learning models (bottom group) which is inspired by the study from Wang et al [2]."

- Figure 2: Please add more meaningful legends (e.g., title of legend; change numbers to Yes, No, etc.).

Response:

We have now added more details to the legend of Figure 2.

- Figure 2 a & b: What do the dendrograms relate to?

Response:

The dendrogram represents the similarity between the columns (datasets and the metrics respectively). To be more specific, in Figure 2a, the similar datasets are grouped together according to their feasibility code which is either 0 or 1. In Figure 2b, the evaluation metrics are grouped together according to their feasibility on those methods.

We have now updated our Figure 2 legend with this explanation included.

The new version is

“(a) Summary for method flexibility and computational efficiency. Row: methods; Column: datasets; Dendrogram: similarity among datasets; Legend: (1) Left panel indicators 0, 1 and 2, where 0 represents “no” , 1 represents “yes” and 2 represents “the other case” for the corresponding questions listed here. Is input type numeric only? Yes: numerical only. No: both numerical and categorical are ok. Is output type survival risk? Yes: survival risk. No: survival probability. Can the model handle n<p situation? Yes: it can. No: it cannot. The other case: output is the rank of survival risk. (2) feasibility where red (1) means feasible and blue (0) means not feasible. (3) main indicators including datasets characteristics by a color bar with different colors and stars that represent the model are both memory and time consuming. (b) Prediction ability evaluation metric flexibility. Row: methods; Column: prediction ability evaluation metrics; Dendrogram: similarity among evaluation metrics; Legend: feasibility where red (1) means feasible and blue (0) means not feasible. (c) Rank heatmap for method overall performance. Row: methods; Column: performance metrics; Legend: (1) Rank: red to blue from 1 to 20 where 1 means the top rank. (2) Performance metric categories: 5 different categories representing all metrics used to evaluate method performances. (d) Harrell’s C-index heatmap. Row: datasets; Column: methods; Legend: Harrell’s C-index.“

- Figure 2 d: The c-index is not a proper scoring rule [BKG19] (and only measures discrimination), better use the integrated Brier score (at best, at different evaluation time points) as it is a proper scoring rule and measures discrimination as well as calibration.

Response:

Thank you for this comment. We agree that the C-index alone is not sufficient enough for measuring model predictability. That is why in our study, we considered variants of the C-index as well as the Brier score, the integrated Brier score, and time-dependent AUC for 15 time points. This gives diversity in measuring, assessing and comparing model predictability.

- Figure 3: At which time point is the Brier score evaluated and why at that time point? Consider using the integrated Brier score instead.

Response:

Thank you for this comment. The Brier score was calculated for all time points (i.e. all the event times in the datasets) and then we calculated the sum of all these Brier scores. We have now made this clearer in the manuscript by adding the following in line 221, section 3.2 in the original manuscript.

“The raw Brier score is calculated by taking the sum of all Brier scores for all event times in the dataset.”

In our study, Figure 2b shows that the integrated Brier score (IBS) is only feasible for a limited number of methods. And this is the reason why it is only used for a limited number of methods.

- This is rather subjective, but I find the use of the term "framework", especially that the study contributes by "the development of a benchmarking framework" (l. 60), irritating. For example, a general machine learning framework for survival analysis was developed by Bender et al. [BRSB20], while general computational benchmarking frameworks in R are provided, e.g., by mlr3 [LBR+19] or tidymodels [KW20]. The present study conducts a benchmark experiment with specific design choices, but in my opinion it does not develop a new benchmarking framework. Thus, I would suggest not using the term "framework" but better "benchmark design" or "study design".

Response:

Thank you for this comment. In reflection, we agree and have now changed the name to “benchmark design”.

- In addition, the authors speak of a "customizable weighting framework" (l. 241), but never revisit this weighting scheme in relation to the results and/or provide practical guidance for it. Please explain w.r.t. the results how this scheme can and should be applied in practice.

Response:

Thank you for this comment. We have now provided more guidance on how to use this "customizable weighting framework". The following changes are made in our manuscript. Starting in line 241 of the original manuscript we have added:

"customizable weighting framework (Figure 1b). To apply this in practice, users need to:

(1) Provide a "weight vector" of length q, where each weight represents the strength for each of the q practical aspects. For example, if an urgent analysis is conducted one may prefer a very high weight for computational time. On the other hand, prediction accuracy may be most important in a situation where computational constraints are of no concern.

(2) According to the specific data modality, select the feasible methods (m in total) and obtain their rank (recorded in a m by q matrix) for the aspects considered in the "weight vector".

(3) Multiply the rank matrix and the "weight vector" to obtain the final selected method from this list of m scores (Supp Table 3_Supplementary Material). "

The references need to be revised. A few examples:

- l. 355 & 358: This seems to be the same reference.

Schober,P. and Vetter,T.R. Survival Analysis and Interpretation of Time-to-Event Data. Anesth. Analg. 2018a; 127, 792–798.
Schober,P. and Vetter,T.R. Survival Analysis and Interpretation of Time-to-Event Data: The Tortoise and the Hare. Anesth. Analg. 2018b; 127, 792–798.

- l. 384: Title missing

Breiman, L. Random Forests. *Machine Learning*. 2001; doi: 10.1023/A:1010933404324

- l. 394: Year missing

Yu C-N, Greiner R, Lin H-C, Baracos V. Learning patient-specific cancer survival distributions as a sequence of dependent regressors. Red Hook, NY, USA: Curran Associates Inc; 2011.

- l. 409: Year missing

Holland JH. Adaptation in Natural and Artificial Systems: An Introductory Analysis with Applications to Biology. Control, and Artificial Intelligence MIT Press;1992.

- l. 438: BioRxiv identifier missing

Wang KYX, Pupo GM, Tembe V, Patrick E, Strbenac D, Schramm S-J, et al.. Cross-Platform Omics Prediction procedure: a game changer for implementing precision medicine in patients with stage-III melanoma. bioRxiv. bioRxiv;2020.12.09.415927.

- l. 441: ArXiv identifier missing

Fotso S. Deep neural networks for survival analysis based on a Multi-task framework. arXiv [stat.ML]. 1801.05512.

- l. 445: Journal & Year missing

Wang L. Multi-task survival analysis. 2017 IEEE International Conference on Data Mining (ICDM), IEEE, 2017.p. 485–494.

Typos:
- l. 66: . This
- l. 89: missing comma after the formula
- l. 93: missing whitespace
- l. 107: therefore, (no comma)
- l. 121: where for each, (no comma)
- l. 170: examineS
- l. 174: therefore, (no comma)
- l. 195: as part of A multi-omics study; whitespace on wrong position; the sentence does not appear correct
- l. 323: comes WITH a

Response:
Thank you for these comments. We have accordingly taken these on-board and have made appropriate changes to the manuscript.

Data and code availability

Data and code availability are acceptable. Yet, the ANZDATA and UNOS_kidney data are not freely available and require approval and/or request.
Moreover, for better reproducibility and accessibility, the experiments could be implemented with a general purpose benchmarking framework like mlr3 or tidymodels.
Response:
Thank you for this comment. For both ANZDATA and UNOS_kidney data, they can be obtained through the data application process as made available at https://www.anzdata.org.au/ and https://optn.transplant.hrsa.gov/, respectively. Both data are kidney transplant datasets and their unique data characteristics add important diversity to our benchmark study.

To give some more context, we highlight that some of the considered methods are not yet readily available for both the mlr3 and tidymodels frameworks. As for mlr3, although it includes most of the models we examined for each category, this framework does not have the flexibility to include a feature selection method nested in the 5-fold cross-validation, which we believe to be good practices. This kind of inflexibility is also mentioned in this study [SCS+20].

References

[BKG19] Paul Blanche, Michael W Kattan, and Thomas A Gerds. The c-index is not proper for the evaluation of-year predicted risks. Biostatistics, 20(2):347-357, 2019.
[BRSB20] Andreas Bender, David Rügamer, Fabian Scheipl, and Bernd Bischl. A general machine learning framework for survival analysis.arXiv preprint arXiv:2006.15442, 2020.
[BWSR21] Andrea Bommert, Thomas Welchowski, Matthias Schmid, and Jörg Rahnenführer. Benchmark of filter methods for feature selection in high-dimensional gene expression survival data. Briefings in Bioinformatics, 2021. bbab354.
[HPH+20] Moritz Herrmann, Philipp Probst, Roman Hornung, Vindi Jurinovic, and Anne-Laure Boulesteix. Large-scale benchmark study of survival prediction methods using multi-omics data. Briefings in Bioinformatics, 22(3), 2020. bbaa167.
[KW20] M Kuhn and H Wickham. Tidymodels: Easily install and load the 'tidymodels' packages. R package version 0.1.0, 2020.
[LBR+19] Michel Lang, Martin Binder, Jakob Richter, et al. mlr3: A modern object-oriented machine learning framework in R. Journal of Open Source Software, 4(44):1903, 2019.
[SCS+20] Annette Spooner, Emily Chen, Arcot Sowmya, Perminder Sachdev, Nicole A Kochan, Julian Trollor, and Henry Brodaty. A comparison of machine learning methods for survival analysis of high-dimensional clinical data for dementia prediction. Scientific reports,10(1):1-10, 2020.
[Son21] Raphael Edward Benjamin Sonabend. A theoretical and methodological framework for machine learning in survival analysis: Enabling transparent and accessible predictive modelling on right-censored time-to-event data. PhD thesis, UCL (University College London), 2021.
[VDBSB19] Alexander Volkmann, Riccardo De Bin, Willi Sauerbrei, and Anne-Laure Boulesteix. A plea for taking all available clinical information into account when assessing the predictive value of omics data. BMC medical research methodology, 19(1):1-15, 2019.
[WLR19] Ping Wang, Yan Li, and Chandan K Reddy. Machine learning for survival analysis: Asurvey. ACM Computing Surveys (CSUR), 51(6):1-36, 2019.

#external expert:

After carefully read the manuscript, briefly this topic is interesting and valuable to the readers, the writing is fine, the research and comparison are reasonable but not perfect. Generally, I agree the comments from reviewer 2, the survival analysis methods collected in this paper are originally created to do a special analysis or practical question for a predefined aim or certain data type, thus simple comparison of all the methods in multiple datasets with different data structures is hard to deliver a precise conclusion.

Response:

Thank you for these comments. We agree, the easier comparison to deliver a more precise conclusion should be done targeting a specific category of models or a specific type of disease. However, as we illustrated in our background section, the current research gap lies in the restricted comparison within either a certain modality of the dataset or a particular disease type. Our study aims to compare the various categories of methods on a diverse collection of datasets and that is the reason that we summarize the results into an all-in-one comparison style. It is important to note that the diversity of the datasets is one of the key strengths of this study.

They may category the survival analysis methods based on the different algorithms and data structures, and then perform the comparison specifically, this may be more reasonable and comparable.

Response:

As illustrated above and highlighted in the revised manuscript, studying different categories of survival models based on different data structures is the current research gap investigated in our work. We aim to examine the trade-off between clinical versus omics data, among different categories of methods based on

multiple evaluation criteria. Based on the valuable feedback from the referee reports, we have now made some changes in our study design to make the comparison fairer (as a response to reviewer 2). Specifically, we have decided to use for each of these methods their default choice of hyperparameters as is common practice. The particular values used are detailed in our updated Table 2.

In addition, some descriptions of the concept are inaccurate, e.g. “the class label “0” (referring to a “no-event”) does not mean an event class labelled as “0”, instead, it represents that the event-outcome is “unknown””.

Response:

Thank you for this comment. We have now re-written the sentence to:

“the class label ‘0’ means an observation is censored as its exact event time is not observed.”

A minor comment: suggest to plot colorful lines in figure4 for each survival model, not just cox(GA) and RSF (less tree).

Response:

Thank you for this comment. We believe that for visual communication with more than 10 colors (methods in our case), it is clearer to highlight those particular methods we included in our writing with thicker lines. Accordingly, we have now updated Figure 4 to incorporate both color lines and specific highlights.

Manuscript
Click here to access/download;Manuscript;SurvBenchmark_20220217.docx

SurvBenchmark: comprehensive benchmarking study of survival analysis methods using both omics data and clinical data

Yunwei Zhang[1,2,] Germaine Wong[3,4,5], Graham Mann[6,7], Samuel Muller[1,8^,] Jean Y.H. Yang[1,2,9*^]
[1] School of Mathematics and Statistics, The University of Sydney, Sydney, Australia
[2] Charles Perkins Centre, The University of Sydney, Sydney, Australia
[3] Sydney School of Public Health, The University of Sydney, NSW, Sydney, Australia.
[4] Centre for Kidney Research, Kids Research Institute, The Children's Hospital at Westmead, NSW, Sydney, Australia.
[5] Centre for Transplant and Renal Research, Westmead Hospital, NSW, Sydney, Australia.
[6] John Curtin School of Medical Research, Australian National University, Canberra, Australia
[7] Melanoma Institute Australia, North Sydney, NSW, Australia
[8] Department of Mathematics and Statistics, Macquarie University, Sydney, Australia
[9] Laboratory of Data Discovery for Health Limited (D^{2}4H), Science Park, Hong Kong SAR, China
^ Equal contribution
*To whom correspondence should be addressed.

Associate Editor: XXXXXXX
Received on XXXXX; revised on XXXXX; accepted on XXXXX

Abstract

Survival analysis is a branch of statistics that deals with both, the tracking of time and of the survival status simultaneously as the dependent response. Current comparisons of survival model performance mostly center on clinical data with classic statistical survival models, with prediction accuracy often serving as the sole metric of model performance. Moreover, survival analysis approaches for censored omics data have not been thoroughly investigated. The common approach is to binarise the survival time and perform a classification analysis.

Here, we develop a benchmarking design, SurvBenchmark, that evaluates a diverse collection of survival models for both clinical and omics datasets. SurvBenchmark not only focuses on classical approaches such as the Cox model, but it also evaluates state-of-art machine learning survival models. All approaches were assessed using multiple performance metrics, these include model predictability, stability, flexibility and computational issues. Our systematic comparison design with 320 comparisons (20 methods over 16 datasets) shows that the performances of survival models vary in practice over real-world datasets and over the choice of the evaluation metric. In particular, we highlight that using multiple performance metrics is critical in providing a balanced assessment of various models. The results in our study will provide practical guidelines for translational scientists

and clinicians, as well as define possible areas of investigation in both survival technique and benchmarking strategies.

Key words: survival analysis, machine learning, survival prediction

Contact: jean.yang@sydney.edu.au

1. Background

Survival models are statistical models designed for data that have censored observations, that is time-to-event data, which are ubiquitous, including in health, tourism [1], economics [2], and engineering [3]. In this paper, we will follow the terminology of survival analysis in which the event of interest is captured through a 'status' variable, "s" typically, considered as a binary class outcome. The waiting time to this status event is defined as the 'survival' time, either measured as continuous or discrete time periods. This class of models has wide applicability well beyond clinical and omics applications that were considered in this article. Survival models target both outcomes: status and time-to-event, whereas neither regression analysis on time nor classification analysis on status explain this bivariate outcome information [4]. Classes of models dealing with these events have wide applicability well beyond the clinical and omics applications that are considered here.

Numerous survival models have been developed over the last decades. There are many studies in the literature that give a good overview on right-censored datasets without time-dependent covariates, for example [5]. However, few of these studies take a practical viewpoint, and few make sufficient real-world dataset comparisons, particularly in the biomedical field. This motivated us to develop a benchmarking design for the diverse clinical and omics survival data in health. This work intends to improve the knowledge and understanding of such models, and guide clinical decision making. We first performed an exhaustive search for various types of available survival analysis methods and the methods of performance evaluations for the different types of datasets.

Among the comparative studies that included real-world datasets in health, we found that most have a specific focus such as on a certain disease (e.g. colon cancer), or on a certain data platform (e.g. omics

or clinical). For example, [6] and [7] conduct reviews on classical survival models such as the Kaplan-Meier (KM) method and the Cox Proportional Hazards (CoxPH) model with a focus on clinical data with an induced anaesthesia state and a specific colon cancer type, respectively. [8] apply the penalised Cox model, survival support vector machine (SVM), random survival forest (RSF), and Cox boosting models on large genomic data. To date, there are no systematic review encompasses datasets obtained from multiple disease types. Therefore, this necessitates the development of a benchmarking design that will provide a better understanding of how different survival models perform in practice across various disease types.

With the emergence of different modelling approaches from various disciplines many of these recent comparison studies have limited their focus on either within classical models (KM method, CoxPH model) or within modern machine learning (ML) methods. Recently, a comprehensive survey article by [5] compares three categories of statistical survival and ML methods with a focus on theoretical mathematical details. However, this study does not provide practical implications of the various methods and no comparison of performance using real-world datasets is made. There is a need for better guidance on what data analysis strategy to use.

A recent exception is the benchmark study by [9]. This valuable contribution includes both real-world clinical and omics datasets and analyses these with classical regression and modern ML methods with particular focus on the impact of considering the multi-omics structure to the survival model predictability. However, this study includes cancer diseases only and datasets are solely obtained from 'The Cancer Genome Atlas' (TCGA). There remains a pressing need to look into more diverse and thus more heterogenous datasets coming from multiple databases to benchmark the survival model performances from more diverse aspects.

To this end, we develop a benchmarking design SurvBenchmark that considers multiple aspects with several evaluation metrics on a large collection of real-world health and biomedical datasets which guides right-censored data survival method selection and new method development.

2. Survival models and their evaluation

Survival models can deal with data that explain censored observations with a bivariate outcome variable, consisting of ‘time’ (the minimum of ‘time-to-event’ and ‘censoring time’) and ‘event’ (binary: “class 1”= “event did occur”, “class 0”= “otherwise”). There are two key features of such censored survival objects. Firstly, ~~the class label “0” (referring to a “no-event”) does not mean an event class labelled as “0”, instead, it represents that the event-outcome is “unknown”, or, using the technical term, “censored”.~~ the class label ‘0’ means an observation is censored as its exact event time is not observed. Secondly, an additional tracking time measurement is included as part of the response.

There are two main branches of survival models: classical statistical survival models, which include parametric, nonparametric and semi-parametric models; and modern ML survival models, which include ensemble-based methods and state-of-the art deep learning based approaches. Both sets of models are briefly reviewed in the following sections.

2.1 Classical survival models

The Cox Proportional Hazards (CoxPH) model [10] is the most widely used classical survival model. CoxPH works on the hazard function, which is given by

$$h(t,x) = h_0(t)e^{(\sum_{j=1}^{p}\beta_j x_j)}, \qquad (1)$$

where $x = (x_1, x_2, \ldots, x_p)$ is the covariate vector and $h_0(t)$ is the baseline hazard function. CoxPH is a semi-parametric model and the baseline hazard function is canceled out when taking the ratio of two hazard functions.

The penalised Cox model is another extension of the CoxPH model that helps to prevent overfitting. The L1 regularized CoxPH model adds a scaled sum of absolute values of the magnitude of model coefficients, that is $\lambda_1 \sum_{j=1}^{p}|\beta_j|$, as the regularization term to the partial log-likelihood. Other regularizers can be used such as L2 regularization, that is $\lambda_2 \sum_{j=1}^{p}(\beta_j)^2$, or other scaled sums of non-negative penalties of the $\beta's$, such as in the following general penalised partial log-likelihood:

$$\log(L(\beta)) - \lambda \sum_{j=1}^{p} \pi(\beta_j), \qquad (2)$$

where $L(\beta)$ is the partial likelihood as for example given in (Tibshirani, 1997; Equation 2) [11] and then optimization takes place [12] [13] [14]. Using the L1 penalty in Equation (2) gives the Lasso Cox

estimation and using the L2 penalty gives the Ridge Cox solution, respectively. If instead of a single regularization term we consider a weighted average of the L1 and L2 penalty, we obtain the Elastic Net Cox model. One remarkable characteristic of the Lasso Cox model and the Elastic Net Cox model is that they can simultaneously perform feature selection and prediction, because some of the beta parameters can be penalised all the way to 0 when maximizing (2). Notice that the various types of regularization terms can also appear in the loss function of modern ML methods which we introduce in Section 2.2.

2.2 Modern machine learning models

There have been a growing interest in the use of modern ML methods in health as a result of their exceptional performance in many other areas, such as in finance [15], environment [16] and internet of things [17]. Notable examples in health include the application of Random Survival Forest (RSF) on complex metabolomics data [18] and SurvivalSVM to the survival of prostate cancer patients [19]. Both approaches are survival analysis extensions to two widely used ML algorithms for binary classification, namely Random Forest and SVM.

SurvivalSVM was developed by Van Belle et al [20] for time-to-event data. It is a variant of the regularized partial log-likelihood function (2) above but has a different penalty term. In contrast to using $\lambda \sum_{j=1}^{p} \pi(\beta_j)$, SurvivalSVM uses penalised splines and then applies both, ranking constraints and regression constraints to the corresponding partial log-likelihood function. SVM with those constraints enables models for high-dimensional omics data to have more flexible structure, e.g. additive (non)-linear models. One distinct feature of SurvivalSVM is that it treats the prognostic problem as a ranking problem and therefore the estimation of the hazards is not directly incorporated in the model.

RSF was first proposed by [21] as an extension of Random Forest to model censored survival data. Random Forest [22] is a non-parametric bagging-based ensemble learning method that adds variation in the training datasets by bootstrapping the data. Multiple models are generated based on many resamples. The ensemble prediction result is then an average of these multiple models or the result of a majority vote. The key components in our application of RSF are that we use Harrell’s C-index to

evaluate the survival tree instead of the mean square error for regression problems or confusion matrix for classification problems, and that we use the log-rank score in each node as the stopping rule.

Another ensemble-based approach is the boosting method, which contains multiple learners and sequentially gives more weight to weak learners to enhance predictability. For example, the Cox boosting model [23] [24] [25] [26] is developed based on Cox models with boosting being applied to the estimation of the regression parameter vector β in Equation (1). There are two popular approaches to update β: the first is the model-based approach that leads to the mboost method, the second is the likelihood-based approach that leads to the CoxBoost method (benchmarked in this study).

These models so far only focus on optimising a single objective. Because survival data is time dependent, it is natural to have multiple tasks related to one or more time points of interest. This naturally leads to multi-task learning, a method that deals with the need to predict for more than a single response variable, based on joint optimization of multiple likelihood functions corresponding to each task. The multi-task logistic regression model (MTLR) by Yu et al. [27] is a survival model for multiple time points, where for each the task is to predict survival using a logistic regression model and the parameters from each model are estimated simultaneously in the maximization of the joint likelihood function.

More recently, the ML and artificial intelligence communities refer to the methods described above as classical ML methods due to the emergence of deep learning (DL), a conceptual advancement based on neural networks (NN). In survival analysis, a number of DL survival models were developed such as Cox-nnet [28], DeepSurv [29] and DeepHit [30]. The key concept here is having different loss functions that particularly target either the hazard or the survival probability for those neurons in hidden layers when building the DL architecture. High dimensional complex biological information can be better represented with the application of those hidden layers [31] and through relaxing the proportional hazard assumption.

2.3 Feature selection methods applied to survival models

The input features are fundamental to every statistical or machine learning model, and the survival model is no exception [32]. Wrapper and filter [33] are two feature selection methods that are widely used for not only regression and classification models, but also survival models.

The wrapper approach is a model-dependent method in which the performance of the model determines the selection of subsets of features. Stepwise feature selection approaches fall into this category since one feature is deemed to be included or deleted as the model's performance improves. Other more computational approach such as the genetic algorithm (GA) [34], which was originally developed to solve an optimization problem, has been extended to use as a feature selection approach [35]. The main idea is to start with an initial set of features to then replace it with one that includes features from other parts of the data to optimize the classification accuracy based on a linear discrimination analysis model.

The filtering approach, on the other hand, is a model-independent feature selection method that produces a subset of features without involving the models. This step often occurs outside and before building prediction models. Many of these strategies select features using hypothesis testing statistics from a univariate study. With the advent of omics data in the 1990s, the statistics community embraced the development of differential expression (DE) analysis, which is a filter type feature selection method for identifying promising genes/features using "parallel univariate strategies" based on linear modelling [36].

2.4 Classical performance evaluation metric for survival data

Classically, survival analysis is evaluated in three broad settings: the concordance index, the Brier score and the time-dependent AUC. Similar to evaluating classification and regression models, metrics for calibration and discrimination are developed with incorporating censoring by applying rank-based methods or error based methods together with a weighting scheme.

2.4.1 C-index and its extension in survival analysis

C-indices in survival analysis are concordance-based methods, where 'concordance' measures how close a prediction is to the truth. The original C-index for survival analysis was introduced by Frank. E. Harrell [37], as a time-independent performance measure. C-indices range from 0 to 1, where 1 means perfect performance and 0 means worst possible performance. If a model would not take into account

any information from the data, that is a random prediction is made, then the corresponding C-index would be around 0.5. For most clinical datasets, a C-index around or larger than 0.6 is considered an acceptable prediction. Harrell's C-index [38] defines concordance by looking at ranks of pairs of subjects in the data (there are n choose 2 pairs for data with n subjects). Harrell's C index further depends on the censoring distribution of the data, is motivated by Kendall's tau statistic and is closely related to Somers' D. When ranking the subjects, censored subjects are excluded; and pairs included in the formula are only those comparable, non-censored pairs. There are different versions of the C-index, where the differences come from the different ways that censored subjects are ranked.

We will use the following three concordance indices: Begg's C-index, Uno's C-index and GH C-index. First, Begg's C-index [39] uses KM estimation to incorporate both censored and uncensored subjects by assigning different weight to them. Second, Uno et al [40] develop a new way to calculate the rank with the help of inverse probability of censoring weight (IPCW). Third, the GH C-index [41] changes the concordance function into a probability function based on the Cox model estimation and then approximates its distribution which is robust to censoring.

2.4.2 Brier score

The Brier score [42] [43] uses IPCW to handle censored subjects when measuring discrepancy between the estimated values and the actual values. This score can be considered as a similar measure to the mean squared error (MSE) in regression models to some extent. Like the MSE, the Brier score takes a value greater than 0 that depends on the data and the smaller the Brier score the better. However, to have better interpretability, the integrated Brier score (IBS) is introduced which also takes values between 0 and 1 - it averages the loss over time in situations where there is no interest in a particular time point but performance is with regards to all time points as a whole.

2.4.3 Time-dependent AUC

The time-dependent AUC is inspired from binary classification model evaluations. The receiver operating characteristic (ROC) curve is a classical model assessment plot that examines the relationship between the sensitivity and the false positive rate. The area under the ROC curve is termed AUC (area

under the curve). In survival analysis, event statuses are changing over the time, which requires a dynamic measurement to discriminate the predicted versus the actual. Chambless and Diao [44] were the first to propose a time-dependent AUC for survival analysis. They define the AUC(t) as the probability that a person with disease onset by time t has a higher score than the person with no event by time t. Changes of model predictability for different time points can therefore be visualized by time-dependent AUC curves, which allows people to compare long time versus short time predictability.

3. Material and Methods

3.1 Datasets: six clinical and ten omics data sets

Clinical datasets - Six clinical datasets with different sample sizes and disease types are selected (see references in Table 1).

- Veteran data is a survival dataset from the randomised trial of two treatment regimens for lung cancer obtained from the R package “survival”. There are 6 measured features in this data.
- Pbc data (5 clinical feature, 312 patients) from the Mayo Clinic trial in primary biliary cirrhosis (Pbc) of the liver conducted between 1974 and 1984; obtained from the R “RandomForestSRC”.
- Lung data (7 features, 228 patients) contains patient survival information with advanced lung cancer from the North Central Cancer Treatment Group and is available from the R package “survival.
- ANZ data (ANZDATA): Australia & New Zealand Dialysis and Transplant Registry data containing graft survival information and electronic clinical records for kidney transplantation recipients in Australia and New Zealand from 30th June 2006 to 13th November 2017. This data contains records for both living and deceased donors and also multi-organs transplants. We processed the raw data, restricting the transplant date to be after 2008-09-18 and retained deceased donor kidney transplants only. Missing records are excluded, resulting in 3323 patients and 38 features containing patient, donor and donor-recipient human leukocyte antigen (HLA) compatibility.
- UNOS_Kidney data: Organ transplant data based on the Organ Procurement and Transplantation Network (OPTN)-United Network for Organ Sharing (UNOS) in the US (based on OPTN data as

of March, 2020). We selected a random sample of 3000 records associated with deceased donor kidney transplantation only with 99 features containing recipients, donors and donor-recipient HLA compatibility. Missing values are imputed using the R package “MICE”.

- Melanoma_clinical data, extracted from melanoma data [45] [46]: A in-house dataset collected as a part of a multi-omics study. This is the part that contains clinical information for patients. After deleting all missing values, we have 88 patients with stage three melanoma disease measured by 14 clinical features.

Omics datasets - We consider eight published data and two in-house melanoma cancer datasets. A summary of the size and censoring rate of all datasets can be found in Table 1.

- Two ovarian cancer gene expression datasets, downloaded from the R package “curatedOvarianData”. Curation and analysis pipeline of this data follow [47]. Ovarian1 is the "GSE49997_eset" data (194 patients/16047) genes, Ovarian2 is the “GSE30161_eset” data (58/19816).
- ~~Another six gene expression datasets are available online from work by Yang and colleagues [45], namely GE_1, GE_2, …, GE_6.~~ Another six gene expression datasets are available online from work by Yang and colleagues [48], We have named them GE1 to GE6 for easier rendering of labels in our figures. For GE_3, log2 transformation is applied, followed by a KNN imputation with 10 nearest points. For GE_6, median normalisation is applied. For others, no further pre-processing was performed.
- Melamona_itraq and Melanoma_nano are two in-house melanoma omics datasets [45] [46], the first is a protein expression dataset from the iTRAQplatform and the second is a Nanostring dataset from the above melanoma study and pre-processing steps are described in the respective papers. The itraq protein expression data has 41 patients with 640 proteins. The nanostring data has 45 patients with 204 genes [49], and the GEO ID is “GSE156030".

3.2 Benchmarking design/procedure

Evaluation metrics: We examine model performance metrics that can be broadly grouped into four categories and assess performance in terms of each methods' flexibility, predictability, stability and computational efficiency detailed in Supp Table 1_Supplementary Material and briefly summarized as follows:

(i) We measure *model flexibility* by looking at whether a given method can handle different data modality, different level of sparsity, and represents multiple ways including the type of data required (clinical, omics), type of input required (categorical, numerical), sparsity of the data allowed (yes, no) and prediction ability evaluation metrics allowed.

(ii) We measure *model predictability* using three different metrics: C-index, time-dependent AUC and Brier score. We apply four different modified versions of C-index: Harrell's C-index, Begg's C-index, Uno's C-index and GH C-index. For identification of different time points, we equally divided the survival time ranging from the 1st quartile to the 3rd quartile into fifteen time points for each dataset, and therefore, we obtained fifteen AUC values corresponding to each time point. As for the Brier score, we calculated the raw Brier score and the IBS. The raw Brier score is calculated by taking the sum of all Brier scores for all event times in the dataset.

(iii) We measure the *model computational efficiency* using both computational time and memory. Computational time is calculated using the "Sys.time" function in R. Memory is calculated using the "Rprof" function in R and the total memory used is summarised for each experiment.

(iv) We measure *model stability* using model reproducibility and the standard deviation (SD) of model predictability metrics. Model reproducibility is defined as the proportion of successful runs among all the runs attempted. For each model predictability metric, we calculated its SD. We then ranked the values for all the methods for each dataset from the most stable (smallest SD) to the least stable (largest SD).

Benchmarking methods: All methods evaluated are described in Table 2 (details in Supplementary Data). In this benchmark study, hyperparameter sets used in these methods are chosen to be the default set. All compared methods (Supplementary Data) and evaluation metrics (Supp Table 1_Supplementary Material) are applied and evaluated on real-world datasets listed in Section 3.1. We apply 20 times (runs) repeated 5-fold cross validation using RStudio server with 15 cores in parallel. For each run, the

whole data is split into a training dataset (80%) and a testing dataset (20%) with each method trained using the training dataset and values of evaluation metrics calculated using the testing dataset. For methods with a feature selection step, a nested feature selection step is applied on those training folds within each 5-fold cross validation procedure. Detail about the packages and parameters can be found in (Supplementary Data) and functions used to evaluate the methods are shown in (Supp Table 1_Supplementary Material).

4. Results

4.1 Comprehensive benchmarking design

To comprehensively evaluate the strength and weakness of the survival analysis approaches, we select 20 representative methods from our extensive literature review and study their performance when applied to 16 diverse datasets. The performance of each method is measured against 11 metrics representing multiple aspects, including feasibility, predictability, stability and computational efficiency. There are three key aspects of our comparison design SurvBenchmark as depicted in Figure 1: (i) Practical focus through applying the design to a broad range of datasets and by including a taxonomic methods system that evaluates multiple aspects; (ii) Extensive comparison of methods from classical to state of the art ML approaches; (iii) Comprehensive evaluation of the model performance with the utilisation of a customizable weighting framework (Figure 1b). To apply this in practice, the following steps are needed:

(1) Provide a "weight vector" of length q, where each weight represents the strength for each of the q practical aspects. For example, if an urgent analysis is conducted one may prefer a very high weight for computational time. On the other hand, prediction accuracy may be most important in a situation where computational constraints are of no concern.

(2) According to the specific data modality, select the feasible methods (m in total) and obtain their rank (recorded in a m by q matrix) for the aspects considered in the "weight vector".

(3) Multiply the rank matrix and the "weight vector" to obtain the final selected method from this list of m scores (Supp Table 3_Supplementary Material).

4.2 Practical consideration in assessing model performance

Many comparison studies define method performance solely in terms of method predictability, with only a few studies taking into account computational time. Often the feasibility of the method is not properly considered or discussed. Practically, it is paramount that a method can be applied to the data at hand, based on both the flexibility (data modality, sparsity) and computational requirement.

Given the diverse collection of data characteristics that is now available in the biomedical field, not all survival approaches are feasible to be applied to all data types. For example, some classical Cox models (Figure 2a, top left from column 1 to 10, row 1 to 4; a blue box indicates 'method not feasible'), e.g. because it cannot handle large p (features) small n (samples) datasets (such as GE-1) which is a distinct feature of any molecular (omics) study. Advanced feature selection methods together with ML survival models such as CoxBoost(DE) can only take numerical data as the input (purple box for input type, where model characteristics are coded using 0, 1 and 2 with questions defined as below. Is input type numeric only? Yes: numerical only. No: both numerical and categorical are ok. Is output type survival risk? Yes: survival risk. No: survival probability. Can the model handle n<p situation? Yes: it can. No: it cannot. The other case: output is the rank of survival risk.).

Next, we look at the computational aspect, and we notice that DL based methods are computationally inefficient as highlighted by the star icon (Figure 2a). From the many rowwise stars, we observe that RSF (5 stars) and MTLR (5 stars) are not as computationally efficient as Cox-based approaches such as Lasso_Cox (1 star) and CoxBoost (0 stars).

Lastly, a summary tabulating the feasibility associated with each of the evaluation metrics for prediction is provided in Figure 2b. The results highlight that Begg's C-index and GH C-index are applicable only for Cox methods (red indicates feasibility), that the integrated Brier score can be calculated for Cox model and RSF (red), and that the Brier score cannot be calculated for SurvivalSVM (blue).

4.3 Performance evaluation from multiple perspectives: no 'one size fits all'

To achieve a comprehensive overview of different survival approaches, we assess method performance from multiple perspectives across a large collection of datasets. Here, we color the methods according to their performances for all three broad categories: model predictability, model stability and computational efficiency (Figure 2c shows ranks of those methods where red means the best and blue the worst; similarly, Figure 2d shows Harrell's C-index values with red referring to high values and blue to small values). We find that no method performs optimally across all three categories and there are various trade-offs among the categories.

For model predictability, we use seven different measures based on C-index, Brier score and time-dependent AUC. Here, MTLR-based approaches perform evidently better than others, which is most apparent by looking at the performance results using C-index and time-dependent AUC. In order to further examine whether MTLR-based approaches have similar performance across all datasets, we show our examination on one specific criteria (the most popular Harrell's C-index; Mean_hc). In Figure 2d we demonstrate that MTLR has optimal performance for all but one of the six clinical datasets with PBC having optimal performance for one of the clinical datasets. Variants of MTLR (MTLR(GA) and MTLR(DE)) outperformed MTLR when applied to any of the ten omics datasets suggesting the performance of the approaches depend on the type of dataset.

For computational efficiency as measured by computational time and memory usage, the best performing methods are classical Cox-based models and CoxBoost. In particular, Cox, Cox_bw_AIC and Cox_bw_BIC are the top three performing methods for computational time (Figure 2c). For model stability, we have seven criteria and they are based on calculating the standard deviation (SD) of predictability metrics described above. Similar to the the computational efficiency performance, when using SD-criteria, Cox, Cox_bw_AIC and Cox_bw_BIC are also the top 3 performing methods in all but one criteria, the exception is the standard deviation of Brier score (SD_bs), where DNNSurv ranks first suggesting its ability to discriminate survival probabilities for different observations.

~~Despite having the best performance in both computational efficiency and stability for Cox-based methods, their predictability falls behind MTLR-based methods. On the other hand, MTLR-based methods clearly have the best predictability but they are not efficient and stable enough [47]. In conclusion, these observations demonstrate that no method performs optimally for all those categories.~~

In conclusion, the above observations demonstrate that no method performs optimally for all those categories. In practice, we recommend first completing a feasibility check first to draw conclusions on time constraints and to heighten awareness of the data types actually present, and then explicitly deciding on the focus of the research, for example that model predictability is the top priority. Our analysis supports the use of MTLR and its variants for both omics and clinical datasets when survival prediction is the key priority, despite the fact that these approaches are inefficient [50]. However, Cox-based models are preferable when comparing the effect of variables, such as the treatment effect for clinical datasets, because of their efficiency and interpretability.

4.4 Cox-based modern ML methods have similar prediction performance compared to classical Cox-based methods

To understand the gain in model predictability from Cox-based modern ML methods (CoxBoost, Coxboost (GA)), we compare these models with classical Cox-based methods (Lasso_Cox, EN_Cox) which are used as a gold standard method in many studies. Our results indicate that they have similar performance (Figure 3) across a large collection of datasets. For example, in the ANZ data, which is a representative clinical dataset, we observe similar model predictability measured by both Harrell's C-index and Brier score. For GE_5, a representative dataset of omics with large p small n data characteristics, the same conclusion is drawn. This suggests the performance of modern ML methods in complex health and clinical data is not as clear cut as in some other domains.

4.5 Data dependent model performance for different time

To study the model performance over time, we visualize this using the time-dependent AUC curves for all methods. Here we observe among two representative clinical datasets (PBC, UNOS_Kidney) and two omics datasets (GE_2, GE_4) in Figure 4, not all curves are parallel to each other, indicating that the behaviour of model predictability for different time points is data dependent (see Supp Figure 1_Supplementary Material for further results).

We pick two representative models (Cox(GA) & RSF) to demonstrate this data dependent model behaviour. For UNOS_Kidney data and GE_2 data, the curves are approximately horizontal, which

indicates the consistency of short-time, medium time and long-time model predictability. In contrast, for PBC data and GE_4 data, model predictability changes along those time points.

5. Conclusions

This benchmark study comprehensively evaluated the relevance and usefulness of survival models in practice, where emphasis is on performance over diverse datasets. In our review we assessed a broad variety of survival methods from classical Cox-PH models to modern ML models. The findings of our systematic assessment will provide specific guidance for translational scientists and clinicians, as well as define areas of potential study in both survival methodology and benchmarking strategies.

In recent years, there is a clear shift in how survival data is analysed, from modelling directly the hazard function to building models directly on survival functions. Conceptually, modelling hazard functions is a good way to identify key risk factors related to various patients' risk levels. On the other hand, if our key criterion is to predict accurately survival, modelling survival probability directly improves predictability. Methods including MTLR, DNNSurv and SurvivalSVM which directly model the survival function showed better performance in terms of model predictability, this is consistent with what [27] have commented on when discussing the performance of their proposed MTLR method.

It is striking that MTLR shows remarkably high model predictability in our benchmark study. We now highlight technical advantages, disadvantages as well as its applications. Numerous reasons could contribute to the better model prediction performance of the MTLR-based approaches. These include the three main reasons as discussed by [27]: direct modeling of the survival function, simultaneous building of multiple logistic regression models, and dynamic modeling. Interestingly, the majority of extended MTLR models since 2011 are based on neural networks as researchers extend the concept to account for nonlinearity in datasets [51]. To date, only a limited number of studies have applied MTLR in health using clinical data in HIV patients [52] or on large omics datasets to predict patient survival in breast and kidney cancers [53]. Given its outstanding model predictability observed for most of the datasets in our study, we believe there is opportunity to use MTLR more widely for survival risk modelling in Health contexts.

Model predictability is one of the key metrics to assess survival studies with Harrell's C-index being currently the most popular. As this kind of ranking based concordance measurement is suitable to evaluate predicted outcomes with censored data, various concordance indices are developed using different methods to handle censoring such as Uno's C-index using IPCW. Besides concordance indices, other predictability metrics such as the time-dependent AUC, which applies a similar idea as the AUC in binary classification but divides the whole time interval into multiple time points, are also adopted in some survival studies [54]. Given that model predictability could be measured by multiple types of indices, we suggest that hybrid evaluation metrics should be applied in practice to provide relatively comprehensive assessments for the fitted model.

While many survival approaches are applicable to both clinical and omics data, there are a number of recently developed approaches that are specifically tailored for high dimensional omics data, such as CoxBoost. The rationale behind developing data-specific methods is to better capture the distinct data characteristics in either the clinical or omics studies. Clinical data usually include mixed modality variables, large sample sizes but have large n (observations) and small p (features). In contrast, omics data naturally comes with a large collection of molecular features and with small n but their data type is homogenous. When it comes to various real-world datasets, performances are also affected by many other aspects besides data type (clinical, omics) such as data modality and therefore, it is challenging to directly examine whether those tailored methods indeed improve the performance. Further examination of the aspects that affect model predictability can be found in Supp Table 2_Supplementary Material.

~~Unlike in other approaches, there are some reproducibility concerns for the method DNNSurv in our empirical work: It failed to run for some of the cross-validation runs. Specifically,~~ Deep learning based methods failed for some datasets on some cross-validation runs. Taking the method DNNSurv as an example, among all 100 runs, DNNSurv had a 100% completion rate for 5 out of the 12 applicable datasets (Supp Figure 2_Supplementary Material) only. For the remaining 7 datasets, completion rate was around 80% and as low as 63% for the Melanoma_itraq data. This instability is likely due tuning parameter sensitivity when sample size is small [55]. All failed iterations are not recorded when generating the results.

Declarations

Data and code availability

For the ANZDATA, data request can be made through the ANDATA registry, and access to the data source will require HREC approvals. For the UNOS_kidney data, it can be requested from https://optn.transplant.hrsa.gov/data/. Codes for running those methods and evaluation measurements for an example dataset is available at https://github.com/SydneyBioX/SurvBenchmark.

Funding

The following sources of funding for each author, and for the manuscript preparation, are gratefully acknowledged: Australian Research Council Discovery Project grant (DP170100654) to JYHY and SM, Australian Research Council Discovery Project grant (DP210100521) to SM, AIR@innoHK programme of the Innovation and Technology Commission of Hong Kong to JYHY. Research Training Program Tuition Fee Offset and Stipend Scholarship and the Dean's International Postgraduate Research Scholarship (DIPRS) to YZ. The funding source had no role in the study design; in the collection, analysis, and interpretation of data, in the writing of the manuscript, and in the decision to submit the manuscript for publication.

Competing interests

The authors declare that they have no competing interests.

Authors' contributions

JYHY and SM conceived, designed and funded the study with guidance from GW and GM. GM and GW provided access to in-house data, and jointly develop the problem formulation of the study with JYHY and SM. YZ developed the benchmarking design, implemented all the models in R and the evaluation design with guidance from JYHY and SM. YZ, JYHY and SM wrote the manuscript and all authors read and approved the final version of the manuscript.

Acknowledgements

The authors thank all their colleagues, particularly at The University of Sydney, Sydney Precision Bioinformatics Alliance and Charles Perkins Centre for their support and intellectual engagement.

References

1. Thrane C. Analyzing tourists' length of stay at destinations with survival models: A constructive critique based on a case study. Tourism Management. 2012. p. 126–32.

2. Ancarani A, Di Mauro C, Fratocchi L, Orzes G, Sartor M. Prior to reshoring: A duration analysis of foreign manufacturing ventures. International Journal of Production Economics. 2015. p. 141–55.

3. Esmalian A, Dong S, Mostafavi A. Susceptibility curves for humans: Empirical survival models for determining household-level disturbances from hazards-induced infrastructure service disruptions. Sustainable Cities and Society. 2021. p. 102694.

4. Schober,P. and Vetter,T.R. Survival Analysis and Interpretation of Time-to-Event Data. Anesth. Analg. 2018a; 127, 792–798;

5. Wang P, Li Y, Reddy CK. Machine learning for survival analysis. *ACM Comput Surv*. Association for Computing Machinery (ACM); 51:1–362019;

6. Schober,P. and Vetter,T.R. Survival Analysis and Interpretation of Time-to-Event Data: The Tortoise and the Hare. Anesth. Analg. 2018b; 127, 792–798;

7. Ahmed FE, Vos PW, Holbert D. Modeling survival in colon cancer: a methodological review. *Mol Cancer*. Springer Nature; 6:152007;

8. Lee S, Lim H. Review of statistical methods for survival analysis using genomic data. *Genomics Inform*. Korea Genome Organization; 17:e412019;

9. Herrmann M, Probst P, Hornung R, Jurinovic V, Boulesteix A-L. Large-scale benchmark study of survival prediction methods using multi-omics data. *Brief Bioinform*. Oxford University Press (OUP); 2021; doi: 10.1093/bib/bbaa167.

10. Cox DR. Regression models and life-tables. *J R Stat Soc*. Wiley; 34:187–2021972;

11. Tibshirani R. The lasso method for variable selection in the Cox model. *Stat Med*. Wiley; 16:385–951997;

12. Van Houwelingen HC. The elements of statistical learning, data mining, inference, and prediction. Trevor Hastie, Robert Tibshirani and Jerome Friedman, Springer, New York, 2001. No. Of pages: Xvi+533. ISBN 0-387-95284-5. *Stat Med*. Wiley; 23:528–92004;

13. Advances in Statistical Bioinformatics: Models and Integrative Inference for. High-Throughput Data Cambridge University Press;

14. Huang H-H, Liang Y. Hybrid L1/2 + 2 method for gene selection in the Cox proportional hazards model. *Comput Methods Programs Biomed*. Elsevier BV; 164:65–732018;

15. Gogas P, Papadimitriou T. Machine learning in economics and finance. *Comput Econ*. Springer Science and Business Media LLC; 2021; doi: 10.1007/s10614-021-10094-w.

16. Chen W, Xie X, Wang J, Pradhan B, Hong H, Bui DT, et al.. A comparative study of logistic model tree, random forest, and classification and regression tree models for spatial prediction of landslide susceptibility. *Catena*. Elsevier BV; 151:147–602017;

17. Lakshmanaprabu SK, Shankar K, Ilayaraja M, Nasir AW, Vijayakumar V, Chilamkurti N. Random forest for big data classification in the internet of things using optimal features. *Int j mach learn cybern*. Springer Science and Business Media LLC; 10:2609–182019;

18. Dietrich S, Floegel A, Troll M, Kühn T, Rathmann W, Peters A, et al.. Random Survival Forest in practice: a method for modelling complex metabolomics data in time to event analysis. *Int J Epidemiol*. 45:1406–202016;

19. Van Belle V, Pelckmans K, Van Huffel S, Suykens JAK. Improved performance on high-dimensional survival data by application of Survival-SVM. *Bioinformatics*. Oxford University Press (OUP); 27:87–942011;

20. Belle V. Survival SVM: a practical scalable algorithm. ESANN; p. 89–94.

21. Ishwaran H, Kogalur UB, Blackstone EH, Lauer MS. Random survival forests. *Ann Appl Stat*. Institute of Mathematical Statistics; 2:841–602008;

22. Breiman, L. Random Forests. *Machine Learning*. 2001; doi: 10.1023/A:1010933404324;

23. Bin RD, De Bin R. Boosting in Cox regression: a comparison between the likelihood-based and the model-based approaches with focus on the R-packages CoxBoost and mboost. *Comput Statist*. 31:513–5312016;

24. Binder H, Allignol A, Schumacher M, Beyersmann J. Boosting for high-dimensional time-to-event data with competing risks. *Bioinformatics*. Oxford University Press (OUP); 25:890–62009;

25. Binder H, Benner A, Bullinger L, Schumacher M. Tailoring sparse multivariable regression techniques for prognostic single-nucleotide polymorphism signatures. *Stat Med*. Wiley; 32:1778–912013;

26. Binder H, Schumacher M. Incorporating pathway information into boosting estimation of high-dimensional risk prediction models. *BMC Bioinformatics*. Springer Nature; 10:182009;

27. Yu C-N, Greiner R, Lin H-C, Baracos V. Learning patient-specific cancer survival distributions as a sequence of dependent regressors. Red Hook, NY, USA: Curran Associates Inc; 2011;

28. Ching T, Zhu X, Garmire LX. Cox-nnet: An artificial neural network method for prognosis prediction of high-throughput omics data. *PLoS Comput Biol*. 14:e10060762018;

29. Katzman JL, Shaham U, Cloninger A, Bates J, Jiang T, Kluger Y. DeepSurv: personalized treatment recommender system using a Cox proportional hazards deep neural network. *BMC Med Res Methodol*. Springer Science and Business Media LLC; 2018; doi: 10.1186/s12874-018-0482-1.

30. Ryu JY, Lee MY, Lee JH, Lee BH, Oh K-S. DeepHIT: a deep learning framework for prediction of hERG-induced cardiotoxicity. *Bioinformatics*. Oxford University Press (OUP); 36:3049–552020;

31. Ching T, Himmelstein DS, Beaulieu-Jones BK, Kalinin AA, Do BT, Way GP, et al.. Opportunities and obstacles for deep learning in biology and medicine. *J R Soc Interface*. The Royal Society; 15:201703872018;

32. Heinze G, Wallisch C, Dunkler D. Variable selection - A review and recommendations for the practicing statistician. *Biom J*. Wiley; 60:431–492018;

33. Bagherzadeh-Khiabani F, Ramezankhani A, Azizi F, Hadaegh F, Steyerberg EW, Khalili D. A tutorial on variable selection for clinical prediction models: feature selection methods in data mining could improve the results. *J Clin Epidemiol*. 71:76–852016;

34. Holland JH. Adaptation in Natural and Artificial Systems: An Introductory Analysis with Applications to Biology. Control, and Artificial Intelligence MIT Press;1992;

35. Saeys Y, Inza I, Larrañaga P. A review of feature selection techniques in bioinformatics. *Bioinformatics*. Oxford University Press (OUP); 23:2507–172007;

36. Bommert A, Sun X, Bischl B, Rahnenführer J, Lang M. Benchmark for filter methods for feature selection in high-dimensional classification data. *Comput Stat Data Anal*. Elsevier BV; 143:1068392020;

37. Harrell FE. Evaluating the yield of medical tests. *JAMA*. American Medical Association (AMA); 247:2543–61982;

38. Newson R. Confidence intervals for rank statistics: Somers' D and extensions. *Stata J*. SAGE Publications; 6:309–342006;

39. Begg BC, Craemer LD, Venkatraman ES, Rosai J. Comparing tumor staging and grading systems: a case study and a review of the issues, using thymoma as a model. *Statistics in Medicine*. 19:1997–20142000;

40. Uno H, Cai T, Pencina MJ, D'Agostino RB, Wei LJ. On the C-statistics for evaluating overall adequacy of risk prediction procedures with censored survival data. *Stat Med*. Wiley; 30:1105–172011;

41. Gönen M, Heller G. Concordance probability and discriminatory power in proportional hazards regression. *Biometrika*. Oxford University Press (OUP); 92:965–702005;

42. Gerds TA, Schumacher M. Consistent estimation of the expected Brier score in general survival models with right-censored event times. *Biom J*. Wiley; 48:1029–402006;

43. Schmid M, Hielscher T, Augustin T, Gefeller O. A robust alternative to the schemper-henderson estimator of prediction error. *Biometrics*. Wiley; 67:524–352011;

44. Chambless LE, Diao G. Estimation of time-dependent area under the ROC curve for long-term risk prediction. *Stat Med*. Wiley; 25:3474–862006;

45. Mactier S, Kaufman KL, Wang P, Crossett B, Pupo GM, Kohnke PL, et al.. Protein signatures correspond to survival outcomes of AJCC stage III melanoma patients. *Pigment Cell Melanoma Res*. Wiley; 27:1106–162014;

46. Mann GJ, Pupo GM, Campain AE, Carter CD, Schramm S-J, Pianova S, et al.. BRAF mutation, NRAS mutation, and the absence of an immune-related expressed gene profile predict poor outcome in patients with stage III melanoma. *J Invest Dermatol*. Elsevier BV; 133:509–172013;

47. Ganzfried BF, Riester M, Haibe-Kains B, Risch T, Tyekucheva S, Jazic I, et al.. curatedOvarianData: clinically annotated data for the ovarian cancer transcriptome. *Database (Oxford)*. Oxford University Press (OUP); 2013:bat0132013;

48. Yang L, Pelckmans K. Machine learning approaches to survival analysis: Case studies in microarray for breast cancer. *Int J Mach Learn Comput*. EJournal Publishing; 4:483–902014;

49. Wang KYX, Pupo GM, Tembe V, Patrick E, Strbenac D, Schramm S-J, et al.. Cross-Platform Omics Prediction procedure: a game changer for implementing precision medicine in patients with stage-III melanoma. bioRxiv. bioRxiv;2020.12.09.415927;

50. He K, Sun J. Convolutional neural networks at constrained time cost. *2015 IEEE Conference on Computer Vision and Pattern Recognition (CVPR)*. IEEE;

51. Fotso S. Deep neural networks for survival analysis based on a Multi-task framework. arXiv [stat.ML]. 1801.05512;

52. Bisaso KR, Karungi SA, Kiragga A, Mukonzo JK, Castelnuovo B. A comparative study of logistic regression based machine learning techniques for prediction of early virological suppression in antiretroviral initiating HIV patients. *BMC Med Inform Decis Mak*. Springer Science and Business Media LLC; 18:772018;

53. Wang L. Multi-task survival analysis. 2017 IEEE International Conference on Data Mining (ICDM), IEEE, 2017.p. 485–494;

54. Li G, Chen J-Z, Chen S, Lin S-Z, Pan W, Meng Z-W, et al.. Development and validation of novel nomograms for predicting the survival of patients after surgical resection of pancreatic ductal adenocarcinoma. *Cancer Med*. Wiley; 9:3353–702020;

55. Shaikhina T, Khovanova NA. Handling limited datasets with neural networks in medical applications: A small-data approach. *Artif Intell Med*. 75:51–632017;

Figures

Figure 1: SurvBenchmark--Schematic view of our benchmark design.

(a) An overview of survival methods used in this study. We broadly classify current models into two categories; classical statistical models (top group) and modern machine learning models (bottom group) which is inspired by the study from Wang et al [2]. Each of these categories can be further subdivided as presented in the hierarchical chart. All models in blue and red colored boxes are implemented in this current benchmark study. (b) A graphical representation of the SurvBenchmark design. The methods and evaluation metrics are summarised in a matrix with a flexible user defined weights vector.

Figure 2: Summary heatmaps.

(a) Summary for method flexibility and computational efficiency. Row: methods; Column: datasets; Dendrogram: similarity among datasets; Legend: (1) Left panel indicators 0, 1 and 2, where 0 represents “no” , 1 represents “yes” and 2 represents “the other case” for the corresponding questions listed here. Is input type numeric only? Yes: numerical only. No: both numerical and categorical are ok. Is output type survival risk? Yes: survival risk. No: survival probability. Can the model handle n<p situation? Yes: it can. No: it cannot. The other case: output is the rank of survival risk. (2) feasibility where red (1) means feasible and blue (0) means not feasible. (3) main indicators including datasets characteristics by different colours and stars represent the model is both memory and time

consuming. (b) Prediction ability evaluation metric flexibility. Row: methods; Column: prediction ability evaluation metrics; Dendrogram: similarity among evaluation metrics; Legend: feasibility where red (1) means feasible and blue (0) means not feasible. (c) Rank heatmap for method overall performance. Row: methods; Column: performance metrics; Legend: (1) Rank: red to blue from 1 to 20 where 1 means the top rank. (2) Performance metric categories: 5 different categories representing all metrics used to evaluate method performances. (d) Harrell's C-index heatmap. Row: datasets; Column: methods; Legend: Harrell's C-index.

Figure 3: Prediction ability for cox-based methods.

Top left: Harrell's C-index on ANZ data. Top right: Brier score on ANZ data. Bottom left: Harrell's C-index on GE_5. Bottom right: Brier score on GE_5.

Figure 4: Time-dependent AUC curves.

(a) PBC data (b) UNOS_US data (c) GE_4 data (d): GE_2 data. Two selected models: Cox(GA), RSF

Table1. Datasets summary

**Datasets summary**					
**Dataset (name usedin this paper)**	**Number of observations**	**No. of variables**	**Type of data**	**Censoring rate (rounded to 4 decimal places)**	**Reference**
Melanoma_itraq	41	642	Omics	0.4146	Wang,K.Y.X. et al. Cross-Platform Omics Prediction procedure: a game changer for implementing precision medicine in patients with stage-III melanoma.bioRxiv 2020.12.09.415927; doi: https://doi.org/10.1101/2020.12.09.415927
Melanoma_nano	45	206	Omics	0.4222	Wang,K.Y.X. et al. Cross-Platform Omics Prediction procedure: a game changer for implementing precision medicine in patients with stage-III melanoma.bioRxiv 2020.12.09.415927; doi: https://doi.org/10.1101/2020.12.09.415927
Ovarian_2	58	19818	Omics	0.3793	Ganzfried,B.F. et al. (2013) curatedOvarianData: clinically annotated data for the ovarian cancer transcriptome. Database, 2013.
GE_5	78	4753	Omics	0.5641	van 't Veer,L.J. et al. (2002) Gene expression profiling predicts clinical outcome of breast cancer.

					Nature, 415, 530–536.
GE_3	86	6288	Omics	0.7209	Bullinger,L. et al. (2004) Use of Gene-Expression Profiling to Identify Prognostic Subclasses in Adult Acute Myeloid Leukemia. New England Journal of Medicine, 350, 1605–1616.
Melanoma_clinical	88	16	Clinical	0.3939	Wang,K.Y.X. et al. Cross-Platform Omics Prediction procedure: a game changer for implementing precision medicine in patients with stage-III melanoma.bioRxiv 2020.12.09.415927; doi: https://doi.org/10.1101/2020.12.09.415927.
GE_1	115	551	Omics	0.6670	Sorlie,T. et al. (2003) Repeated observation of breast tumor subtypes in independent gene expression data sets. Proc. Natl. Acad. Sci. U. S. A., 100, 8418–8423.
GE-_4	116	4753	Omics	0.5641	van de Vijver,M.J. et al. (2002) A gene-expression signature as a predictor of survival in breast cancer. N. Engl. J. Med., 347, 1999–2009.
Veteran	137	8	Clinical	0.0657	Kalbfleisch,J.D. and Prentice,R.L. (2002) The Statistical Analysis of Failure Time Data. Wiley Series in Probability and Statistics.
Ovarian_1	194	16050	Omics	0.7062	Ganzfried,B.F. et al. (2013) curatedOvarianData: clinically annotated data for the ovarian cancer transcriptome. Database, 2013.
Lung	228	9	Clinical	0.2763	Loprinzi,C.L. et al. (1994) Prospective evaluation of prognostic variables from patient-completed questionnaires. North Central Cancer Treatment Group. J. Clin. Oncol., 12, 601–607.
GE_6	240	7401	Omics	0.4250	Van Houwelingen,H.C. (2004) The Elements of Statistical Learning, Data Mining, Inference, and Prediction. Trevor Hastie, Robert Tibshirani and Jerome Friedman, Springer, New York, 2001. No. of pages: xvi 533. ISBN 0-387-95284-5. Statistics in Medicine, 23, 528–529.
GE_2	295	4921	Omics	0.7322	Beer,D.G. et al. (2002) Gene-expression profiles predict survival of patients with lung adenocarcinoma. Nat. Med., 8, 816–824.
PBC	312	7	Clinical	0.5994	Fleming,T.R. and Harrington,D.P. (2005) Counting Processes and Survival Analysis. Wiley Series in Probability and Statistics.
UNOS_Kidney	3000	101	Clinical	0.7350	OPTN data (https://optn.transplant.hrsa.gov/)
ANZ	3323	40	Clinical	0.8739	ANZDATA (https://www.anzdata.org.au/)

Data table showing the names of datasets used in this paper in the first column. Datasets are ordered by the number of observations (second column, from smallest to largest). Censoring rate is rounded to 4 decimal places.

Table2. Summary of methods used in this study

**Method name**	**Method name in this paper**	**R function name**	**R package name**	**Parameters (default)**
Cox	Cox	coxph	survival	NA
Cox with backward elimination using AIC	Cox_bw_AIC	cph, fastbw	rms	rule="aic", sls=.05,k.aic=2
Cox with backward elimination using p value	Cox_bw_p	cph, fastbw	rms	rule="p", sls=.05
Cox with backward elimination using BIC	Cox_bw_BIC	cph, fastbw	rms	rule="aic",sls=.05,k.aic = log(as.numeric(table(train$status)[2]))
Lasso cox (for clinical datasets)	Lasso_Cox	penalized	penalized	Lambda1=1, lambda2=0
Ridge cox (for clinical datasets)	Ridge_Cox	penalized	penalized	Lambda1=0, lambda2=1
Elastic net cox (for clinical datasets)	EN_Cox	penalized	penalized	Lambda1=1, lambda2=1
Lasso cox (for omics datasets)	Lasso_Cox	glmnet	glmnet	alpha=1, nfolds = 5,type.measure = "C"
Ridge cox (for omics datasets)	Ridge_Cox	glmnet	glmnet	alpha=0, nfolds = 5,type.measure = "C"
Elastic net cox (for omics datasets)	EN_Cox	glmnet	glmnet	alpha=0.5, nfolds = 5,type.measure = "C"

Random survival forest	RSF	rfsrc	RandomSurvivalForest	Default:ntree = 1000,mtry = 10
Multi task logistic regression method	MTLR	mtlr	MTLR	C1=1
DNNSurv (Deep learning survival model)	DNNSurv	multiple functions as in Github codes	DNNSurv	Default: no parameter arguments to be changed by users
Boosting cox model	CoxBoost	coxboost	CoxBoost	stepnumber=10, penalty number=100
Cox model with genetic algorithm as feature selection method	Cox (GA)	GenAlg	GenAlgo	n.features=10 (for omics) , n.features=4 (for clinical) , generation_num=20
Multi task logistic regression model with genetic algorithm as feature selection method	MTLR(GA)	GenAlg	GenAlgo	n.features=10 (for omics) , n.features=4 (for clinical) ,generation_num=20
Boosting cox model with genetic algorithm as feature selection method	CoxBoost (GA)	GenAlg	GenAlgo	n.features=10 (for omics) , n.features=4 (for clinical) , generation_num=20
Multi task logistic regression model with ranking based method as feature selection meth	MTLR(DE)	lmFit,eBayes	limma	n.features=10 (for omics) , n.features=4 (for clinical)
Boosting cox model with ranking based method as feature selection method	CoxBoost (DE)	lmFit,eBayes	limma	n.features=10 (for omics) , n.features=4 (for clinical)
Survival support vector machine	SurvivalSVM	survivalsvm	survivalsvm	Default: sgf.sv = 5, sigf = 7, maxiter = 20, margin = 0.05, bound = 10, eig.tol = 1e-06, conv.tol = 1e-07, posd.tol = 1e-08
DeepSurv (Deep learning survival model)	DeepSurv	deepsurv	survivalmodels	Default: frac = 0.3, activation = "relu", num_nodes = c(4L, 8L, 4L, 2L), dropout = 0.1, early_stopping = TRUE, epochs = 100L, batch_size = 32L
DeepHit (Deep learning survival model)	DeepHit	deephit	survivalmodels	Default: frac = 0.3, activation = "relu", num_nodes = c(4L, 8L, 4L, 2L), dropout = 0.1, early_stopping = TRUE, epochs = 100L, batch_size = 32L

Data table showing the methods used in this benchmark study. R packages and functions with parameters are listed.

Figure1

Click here to access/download;Figure;figure1all.pdf

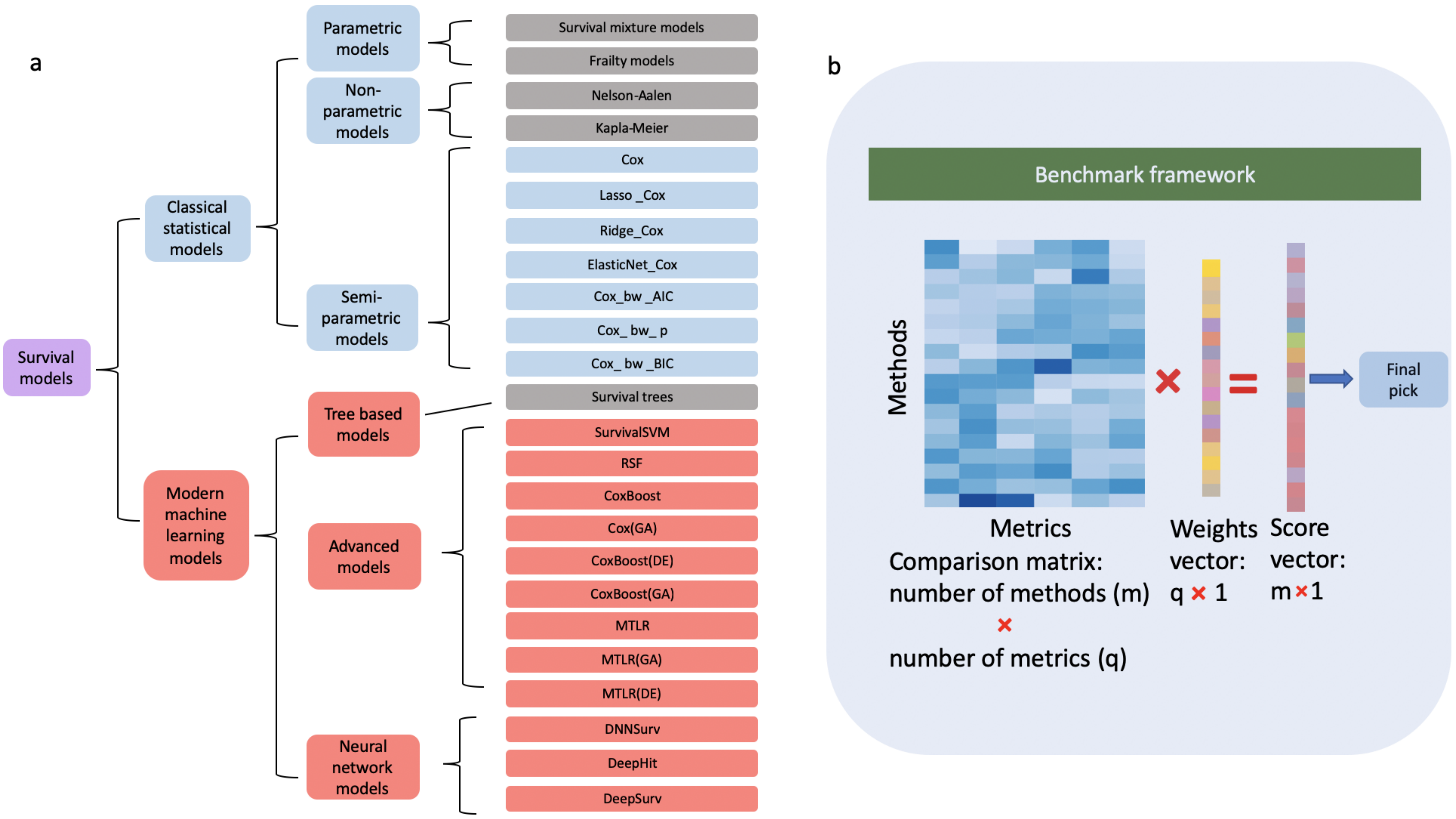

Click here to access/download;Figure;figure2.pdf

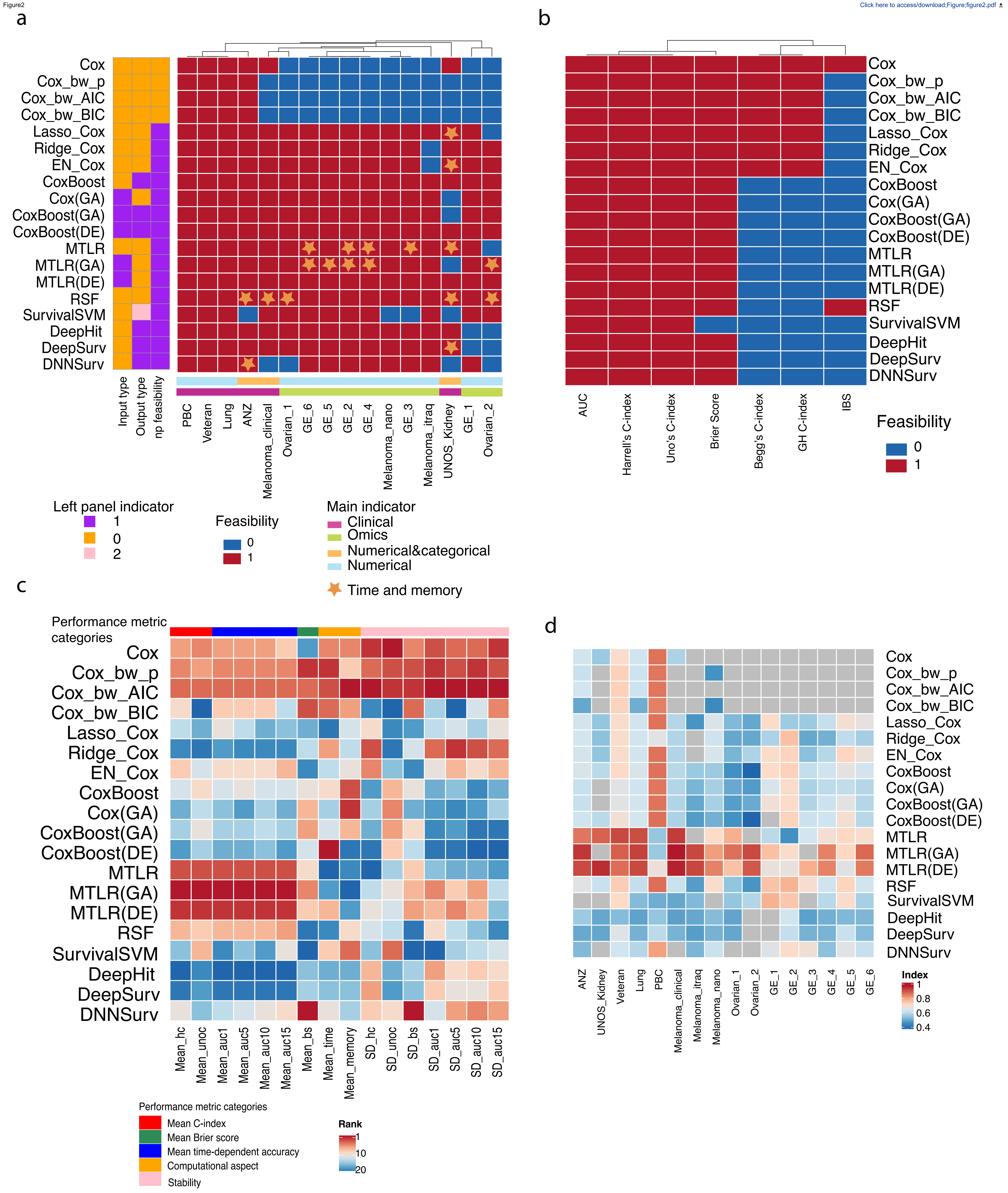

Figure3

Click here to access/download;Figure;figure3.pdf

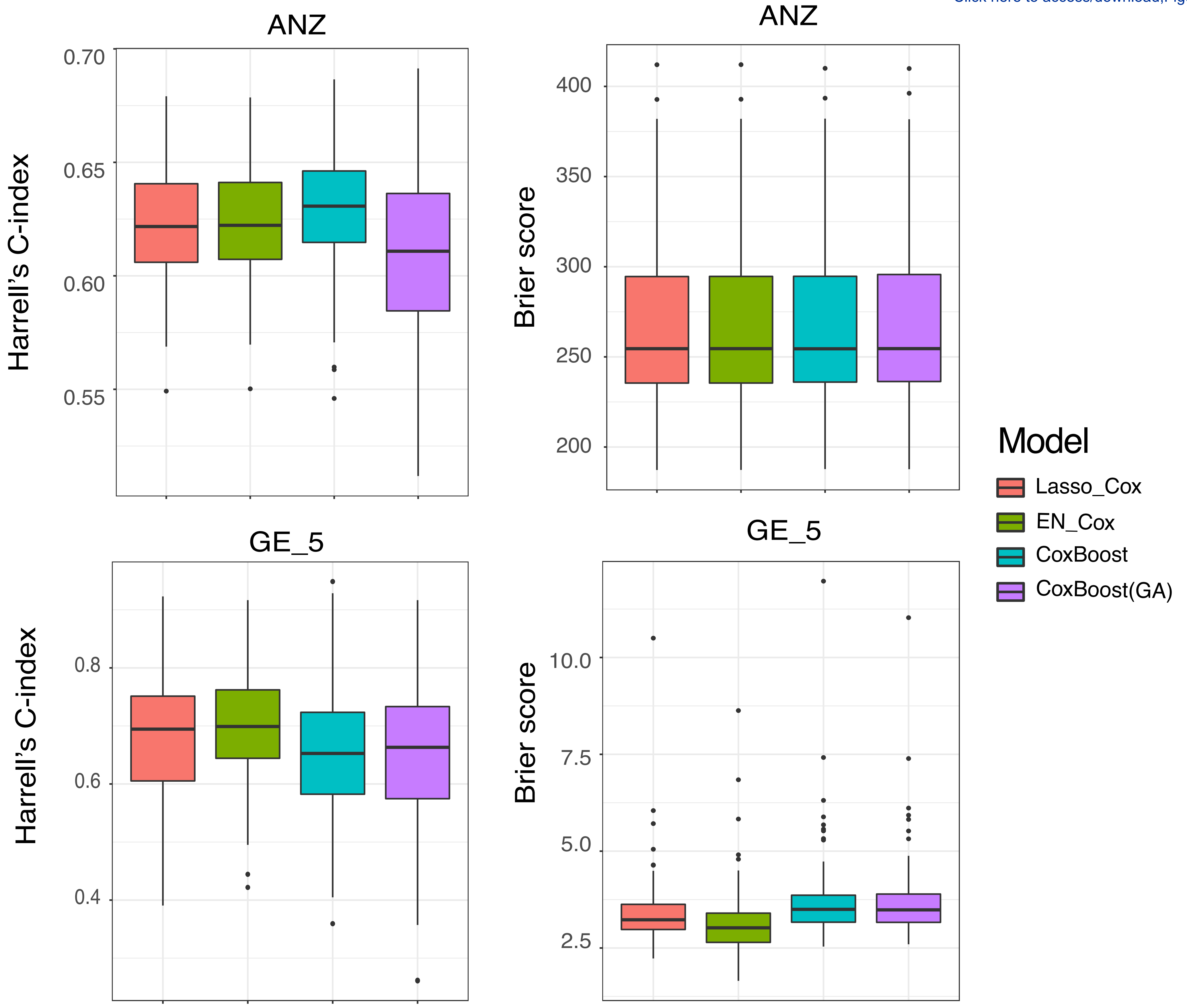

Figure4

Click here to access/download;Figure;figure4.pdf

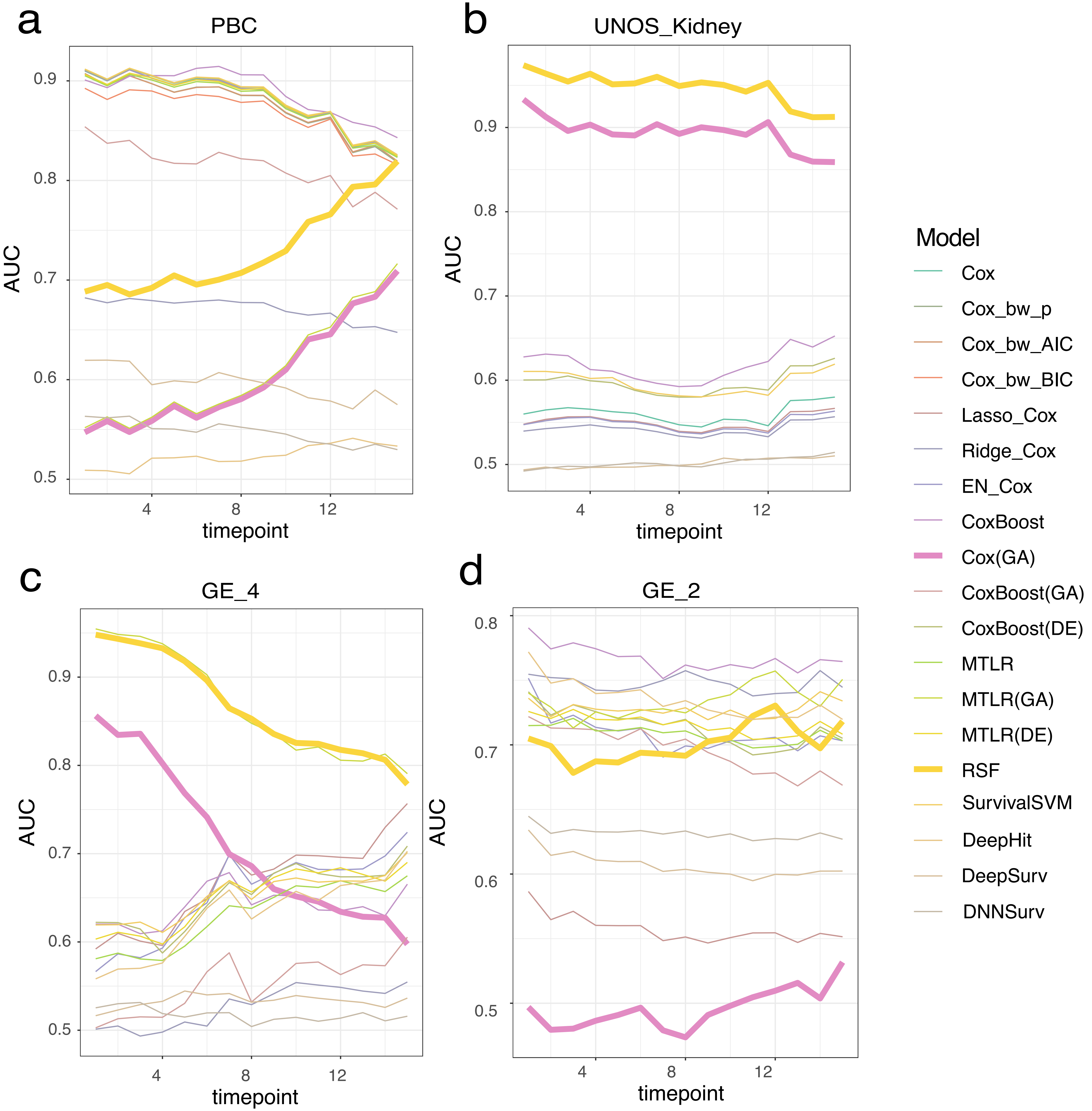

Click here to access/download
Supplementary Material
Supplementary Material.pdf

Click here to access/download
Supplementary Material
Supplymentary Data.xlsx
