# Supplementary material for: SurvBenchmark: comprehensive benchmarking study of survival analysis methods using both omics data and clinical data
Source: Gigascience. 2022 Jul 30;11:giac071. doi: 10.1093/gigascience/giac071 (PMC9338425; doi:10.1093/gigascience/giac071)
Supplement: giac071_GIGA-D-22-00036_Revision_1 [file giac071_giga-d-22-00036_revision_1.pdf]

GigaScience

SurvBenchmark: comprehensive benchmarking study of survival analysis methods using both omics data and clinical data

--Manuscript Draft--

**Manuscript Number:**	GIGA-D-22-00036R1
**Full Title:**	SurvBenchmark: comprehensive benchmarking study of survival analysis methods using both omics data and clinical data
**Article Type:**	Research
**Funding Information:**	Dean’s International Postgraduate Research Scholarship (DIPRS) — Ms. Yunwei Zhang<br>Australian Research Council Discovery Project grant (DP170100654) — Dr. Samuel Muller, Dr. Jean Y.H. Yang<br>Australian Research Council Discovery Project grant (DP210100521) — Dr. Samuel Muller<br>AIR@innoHK programme of the Innovation and Technology Commission of Hong Kong — Dr. Jean Y.H. Yang
**Abstract:**	Survival analysis is a branch of statistics that deals with both, the tracking of time and of the survival status simultaneously as the dependent response. Current comparisons of survival model performance mostly center on clinical data with classic statistical survival models, with prediction accuracy often serving as the sole metric of model performance. Moreover, survival analysis approaches for censored omics data have not been thoroughly investigated. The common approach is to binarise the survival time and perform a classification analysis.<br>Here, we develop a benchmarking design, SurvBenchmark, that evaluates a diverse collection of survival models for both clinical and omics datasets. SurvBenchmark not only focuses on classical approaches such as the Cox model, but it also evaluates state-of-art machine learning survival models. All approaches were assessed using multiple performance metrics, these include model predictability, stability, flexibility and computational issues. Our systematic comparison design with 320 comparisons (20 methods over 16 datasets) shows that the performances of survival models vary in practice over real-world datasets and over the choice of the evaluation metric. In particular, we highlight that using multiple performance metrics is critical in providing a balanced assessment of various models. The results in our study will provide practical guidelines for translational scientists and clinicians, as well as define possible areas of investigation in both survival technique and benchmarking strategies.
**Corresponding Author:**	Jean Yee Hwa Yang, PhD<br>The University of Sydney<br>Sydney, NSW AUSTRALIA
**Corresponding Author Secondary Information:**	
**Corresponding Author's Institution:**	The University of Sydney
**Corresponding Author's Secondary Institution:**	
**First Author:**	Yunwei Zhang
**First Author Secondary Information:**	
**Order of Authors:**	Yunwei Zhang<br>Germaine Wong<br>Graham Mann<br>Samuel Muller<br>Jean Y.H. Yang

Powered by Editorial Manager® and ProduXion Manager® from Aries Systems Corporation

Order of Authors Secondary Information:	
Response to Reviewers:	Reviewer reports:<br>Reviewer #2: Many thanks for the very careful revision of the manuscript. Most of my concerns have been thoroughly addressed. I have only a few remarks left.<br><br>Regarding 1. Fair comparison and parameter selection<br>The altered study design appears much better suited to this end. Thank you very much for the effort, in particular the additional results regarding the two tuning approaches. Although I think a single simple tuning regime would be feasible here, using the default settings is reasonable and very well justified. I agree that this is much closer to what is likely to take place in practice. However, it should be more clearly emphasized that better<br>performance may be achievable if tuning is performed.<br>Response:<br>Thank you for this comment. We agree that it is important to inform readers that suitable tuning may achieve better performance for a particular method.<br><br>We now add to line 414 the following:<br>“In this study we did not survey the extensive tuning procedures for those survival approaches. The main reason being that, in practice, often the default hyperparameter sets are used. Therefore, we also decided to use the default sets in our study here. However, we note that applying targeted tuning methods for a particular dataset may lead to different performances for the considered approaches. “<br><br>Regarding 2. Description<br>Thanks, all concerns properly addressed. No more comments.<br><br>Regarding 3. Reliability<br>I am aware that Figure 2c provides information to this end. I think additional boxplots which aggregate the methods' performance (e.g. for unoc and bs) over all runs and datasets would provide valuable additional information. For example, from Figure 2c one can tell that MTLR variants obtain overall higher ranks based on mean prediction performance than the deep learning methods. However, it says nothing about how large the differences in mean performance are.<br>Response:<br>Thank you for this comment. We agree that boxplots will provide complementary information especially on both the variation and the mean. In our Figure 2c, stability ranking (SD_unoc, SD_bs) measures the variation, i.e. the differences in mean performances. We have chosen to illustrate the information in the main manuscript with the heatmap because we have 16 (datasets) *20 (accuracy metrics)=320 boxplots in total. Each of them has 19 boxes representing the 19 survival approaches. That being said, we have now provided boxplots for unoc and bs in Supplementary Material as Supplementary Figure 3 and Supplementary Figure 4.<br><br>Kaplan-Meier-Estimate (KM)<br>I'm not quite sure I understood the authors' answer correctly. The KM does not use variable information to produce an estimate of the survival function, and I think that is why it would be interesting to include it. This would shed light on how valuable the variables are in the different data sets.<br>Response:<br>Thank you for clarifying this question.Our study does not focus on comparing feature importance differences in different methods, nor does our study compare variable contributions across the datasets. Contrasting variable importance rankings among different methods is beyond the scope of this benchmark study. We would expect such empirical research to demonstrate that finding would be data-dependent.<br><br>Our understanding of the KM method is that Kaplan-Meier curves describe the whole population (data) or describe a subpopulation thereof. Therefore, the KM method does not provide predicted risks or survival probabilities for each individual (observation) beyond learning from to what subpopulation an individual belongs to. KM curves are commonly used to compare two or more subpopulations in the data [Ref 1]. We are aware of one benchmark study [Ref 2] that uses the KM method together with multivariate survival methods. However, when we looked into how the prediction based on KM curves was calculated, we concluded that each observation was assigned the

	same predicted risk in the implementation, which would contribute little to the benchmark study as a baseline method.<br><br>Ref 1: Etikan, I., S. Abubakar, and R. Alkassim. "The Kaplan-Meier estimate in survival analysis." Biom Biostatistics Int J 5.2 (2017): 00128.<br>Ref 2: Herrmann, Moritz, et al. "Large-scale benchmark study of survival prediction methods using multi-omics data." Briefings in bioinformatics 22.3 (2021): bbaa167.<br><br>Regarding 4. Scope and clarity<br>Thanks, all concerns properly addressed. No more comments.<br><br>Minor points:<br>- Since the authors decided to change 'framework' to 'design', note that in Figure 1b it still says 'framework'<br><br>Response:<br>Thanks for pointing this out. We have now changed Figure 1b title to “Benchmark design”.<br><br>- l.51 & l.54/55 appear to be redundant<br>Response:<br>Thank you for pointing this out. We have now deleted the redundant part.<br><br>- Figure 2 a and b:<br>- Please elaborate more on how similarity (reflected in the dendrograms) is defined?<br>Response:<br>Similarity in the degrograms is calculated by the Euclidean distance among those metrics with respect to methods represented by a binary matrix, where 0 means that a metric is not feasible for a method and 1 means a metric is feasible for a method.<br><br>We now add the following in our Figure 2 a and b legend:<br>“Similarity is defined using the Euclidean distance with feasibility indicator 0 and 1. “<br><br>- Why is the IBS more similar to Bregg's and GH C-Index than to the Brier Score?<br>Response:<br>Packages’ availability to calculate the metrics in practice). Therefore, Figure 2b does not show that Figure 2b shows whether a metric is applicable (feasible) for a particular method (based on the R IBS as a prediction accuracy metric performs similarly with Begg’s and GH C-index. Instead, it says that those three metrics (IBS, Begg’s and GH C-index) are not applicable to those methods.<br><br>- Why is the IBS not feasible for so many methods, in particular Lasso_Cox, Rdige_Cox, and CoxBoost?<br>Response:<br>To clarify, our original usage of the phrase “feasible” is in hindsight better phrased as “readily applicable”. In the revised manuscript, we now refer to how readily the methods can be evaluated by the various metrics, i.e. whether the corresponding R functions can be readily applied to our data to calculate the various evaluation metrics (Supplementary Table 1 provides details of the functions/packages used for each evaluation measurements). For example, the IBS calculation (the “crps” function in the R package “pec”; Ref 3) requires a specific type of input that is related to the training model. Currently, the acceptable models must have a “predictSurvProb” method and this is only generated for the Cox model (with “coxph” class) and Random Survival Forest model (with “rfsrc” class). We now re-phrase “feasibility” in our manuscript to “readily applicability” and modify Figure 2b legend to:<br><br>“(b) Prediction ability evaluation metric readily applicable. Row: methods; Column: evaluation metrics; legend: readily applicability where red (1) means readily applicable and blue (0) means not readily applicable.”<br><br>Ref 3: Mogensen UB, Ishwaran H, Gerds TA (2012). “Evaluating Random Forests for Survival Analysis Using Prediction Error Curves.” Journal of Statistical Software, 50(11), 1–23. https://www.jstatsoft.org/v50/i11.).
**Additional Information:**	

Question	Response
Are you submitting this manuscript to a special series or article collection?	No
**Experimental design and statistics**<br><br>Full details of the experimental design and statistical methods used should be given in the Methods section, as detailed in our Minimum Standards Reporting Checklist. Information essential to interpreting the data presented should be made available in the figure legends.<br><br>Have you included all the information requested in your manuscript?	Yes
**Resources**<br><br>A description of all resources used, including antibodies, cell lines, animals and software tools, with enough information to allow them to be uniquely identified, should be included in the Methods section. Authors are strongly encouraged to cite Research Resource Identifiers (RRIDs) for antibodies, model organisms and tools, where possible.<br><br>Have you included the information requested as detailed in our Minimum Standards Reporting Checklist?	Yes
**Availability of data and materials**<br><br>All datasets and code on which the conclusions of the paper rely must be either included in your submission or deposited in publicly available repositories (where available and ethically appropriate), referencing such data using a unique identifier in the references and in the "Availability of Data and Materials" section of your manuscript.	Yes

Have you have met the above requirement as detailed in our Minimum Standards Reporting Checklist?	

Manuscript

Click here to access/download;Manuscript;SurvBenchmark_20220513-

SurvBenchmark: comprehensive benchmarking study of survival analysis methods using both omics data and clinical data

Yunwei Zhang[1,2,] Germaine Wong[3,4,5], Graham Mann[6,7], Samuel Muller[1,8^,] Jean Y.H. Yang[1,2,9*^]

[1] School of Mathematics and Statistics, The University of Sydney, Sydney, Australia
[2] Charles Perkins Centre, The University of Sydney, Sydney, Australia
[3] Sydney School of Public Health, The University of Sydney, NSW, Sydney, Australia.
[4] Centre for Kidney Research, Kids Research Institute, The Children's Hospital at Westmead, NSW, Sydney, Australia.
[5] Centre for Transplant and Renal Research, Westmead Hospital, NSW, Sydney, Australia.
[6] John Curtin School of Medical Research, Australian National University, Canberra, Australia
[7] Melanoma Institute Australia, North Sydney, NSW, Australia
[8] Department of Mathematics and Statistics, Macquarie University, Sydney, Australia
[9] Laboratory of Data Discovery for Health Limited (D[2]4H), Science Park, Hong Kong SAR, China

^ Equal contribution

*To whom correspondence should be addressed, Jean Y.H. Yang: jean.yang@sydney.edu.au

ORCIDs:

Jean YH Yang [0000-0002-5271-2603]; Yunwei Zhang [0000-0003-2173-3185]; Germaine Wong [0000-0001-8422-7269]; Graham Mann [0000-0003-1301-405X]; Samuel Muller [0000-0002-3087-8127];

Abstract

Survival analysis is a branch of statistics that deals with both, the tracking of time and of the survival status simultaneously as the dependent response. Current comparisons of survival model performance mostly center on clinical data with classic statistical survival models, with prediction accuracy often serving as the sole metric of model performance. Moreover, survival analysis approaches for censored omics data have not been thoroughly investigated. The common approach is to binarise the survival time and perform a classification analysis.

Here, we develop a benchmarking design, SurvBenchmark, that evaluates a diverse collection of survival models for both clinical and omics datasets. SurvBenchmark not only focuses on classical approaches such as the Cox model, but it also evaluates state-of-art machine learning survival models. All approaches were assessed using multiple performance metrics, these include model predictability, stability, flexibility and computational issues. Our systematic comparison design with 320 comparisons (20 methods over 16 datasets) shows that the performances of survival models vary in practice over real-world datasets and over the choice of the evaluation metric. In particular, we highlight that using multiple performance metrics is critical in providing a balanced assessment of various models. The results in our study will provide practical guidelines for translational scientists

and clinicians, as well as define possible areas of investigation in both survival technique and benchmarking strategies.

Key words: survival analysis, machine learning, survival prediction

1. Background

Survival models are statistical models designed for data that have censored observations, that is time-to-event data, which are ubiquitous, including in health, tourism [1], economics [2], and engineering [3]. In this paper, we will follow the terminology of survival analysis in which the event of interest is captured through a 'status' variable, "s" typically, considered as a binary class outcome. The waiting time to this status event is defined as the 'survival' time, either measured as continuous or discrete time periods. Survival models target both outcomes: status and time-to-event, whereas neither regression analysis on time nor classification analysis on status explain this bivariate outcome information [4]. Classes of models dealing with these events have wide applicability well beyond the clinical and omics applications that are considered here.

Numerous survival models have been developed over the last decades. There are many studies in the literature that give a good overview on right-censored datasets without time-dependent covariates, for example [5]. However, few of these studies take a practical viewpoint, and few make sufficient real-world dataset comparisons, particularly in the biomedical field. This motivated us to develop a benchmarking design for the diverse clinical and omics survival data in health. This work intends to improve the knowledge and understanding of such models, and guide clinical decision making. We first performed an exhaustive search for various types of available survival analysis methods and the methods of performance evaluations for the different types of datasets.

Among the comparative studies that included real-world datasets in health, we found that most have a specific focus such as on a certain disease (e.g. colon cancer), or on a certain data platform (e.g. omics or clinical). For example, [6] and [7] conduct reviews on classical survival models such as the Kaplan-Meier (KM) method and the Cox Proportional Hazards (CoxPH) model with a focus on clinical data

with an induced anaesthesia state and a specific colon cancer type, respectively. [8] apply the penalised Cox model, survival support vector machine (SVM), random survival forest (RSF), and Cox boosting models on large genomic data. To date, there are no systematic review encompasses datasets obtained from multiple disease types. Therefore, this necessitates the development of a benchmarking design that will provide a better understanding of how different survival models perform in practice across various disease types.

With the emergence of different modelling approaches from various disciplines many of these recent comparison studies have limited their focus on either within classical models (KM method, CoxPH model) or within modern machine learning (ML) methods. Recently, a comprehensive survey article by [5] compares three categories of statistical survival and ML methods with a focus on theoretical mathematical details. However, this study does not provide practical implications of the various methods and no comparison of performance using real-world datasets is made. There is a need for better guidance on what data analysis strategy to use.

A recent exception is the benchmark study by [9]. This valuable contribution includes both real-world clinical and omics datasets and analyses these with classical regression and modern ML methods with particular focus on the impact of considering the multi-omics structure to the survival model predictability. However, this study includes cancer diseases only and datasets are solely obtained from 'The Cancer Genome Atlas' (TCGA). There remains a pressing need to look into more diverse and thus more heterogenous datasets coming from multiple databases to benchmark the survival model performances from more diverse aspects.

To this end, we develop a benchmarking design SurvBenchmark that considers multiple aspects with several evaluation metrics on a large collection of real-world health and biomedical datasets which guides right-censored data survival method selection and new method development.

2. Survival models and their evaluation

Survival models can deal with data that explain censored observations with a bivariate outcome variable, consisting of ‘time’ (the minimum of ‘time-to-event’ and ‘censoring time’) and ‘event’ (binary: “class 1”= “event did occur”, “class 0”= “otherwise”). There are two key features of such

censored survival objects. Firstly, the class label '0' means an observation is censored as its exact event time is not observed. Secondly, an additional tracking time measurement is included as part of the response.

There are two main branches of survival models: classical statistical survival models, which include parametric, nonparametric and semi-parametric models; and modern ML survival models, which include ensemble-based methods and state-of-the art deep learning based approaches. Both sets of models are briefly reviewed in the following sections.

2.1 Classical survival models

The Cox Proportional Hazards (CoxPH) model [10] is the most widely used classical survival model. CoxPH works on the hazard function, which is given by

$$h(t,x) \ = h_0(t)e^{(\sum_{j=1}^{p}\beta_j x_j)}, \qquad (1)$$

where $x = (x_1, x_2, \dots, x_p)$ is the covariate vector and $h_0(t)$ is the baseline hazard function. CoxPH is a semi-parametric model and the baseline hazard function is canceled out when taking the ratio of two hazard functions.

The penalised Cox model is another extension of the CoxPH model that helps to prevent overfitting. The L1 regularized CoxPH model adds a scaled sum of absolute values of the magnitude of model coefficients, that is $\lambda_1 \sum_{j=1}^{p}|\beta_j|$, as the regularization term to the partial log-likelihood. Other regularizers can be used such as L2 regularization, that is $\lambda_2 \sum_{j=1}^{p}(\beta_j)^2$, or other scaled sums of non-negative penalties of the $\beta's$, such as in the following general penalised partial log-likelihood:

$$\log(L(\beta)) - \lambda \sum_{j=1}^{p} \pi(\beta_j), \qquad (2)$$

where $L(\beta)$ is the partial likelihood as for example given in (Tibshirani, 1997; Equation 2) [11] and then optimization takes place [12] [13] [14]. Using the L1 penalty in Equation (2) gives the Lasso Cox estimation and using the L2 penalty gives the Ridge Cox solution, respectively. If instead of a single regularization term we consider a weighted average of the L1 and L2 penalty, we obtain the Elastic Net Cox model. One remarkable characteristic of the Lasso Cox model and the Elastic Net Cox model is that they can simultaneously perform feature selection and prediction, because some of the beta

parameters can be penalised all the way to 0 when maximizing (2). Notice that the various types of regularization terms can also appear in the loss function of modern ML methods which we introduce in Section 2.2.

2.2 Modern machine learning models

There have been a growing interest in the use of modern ML methods in health as a result of their exceptional performance in many other areas, such as in finance [15], environment [16] and internet of things [17]. Notable examples in health include the application of Random Survival Forest (RSF) on complex metabolomics data [18] and SurvivalSVM to the survival of prostate cancer patients [19]. Both approaches are survival analysis extensions to two widely used ML algorithms for binary classification, namely Random Forest and SVM.

SurvivalSVM was developed by Van Belle et al [20] for time-to-event data. It is a variant of the regularized partial log-likelihood function (2) above but has a different penalty term. In contrast to using $\lambda \sum_{j=1}^{p} \pi(\beta_j)$, SurvivalSVM uses penalised splines and then applies both, ranking constraints and regression constraints to the corresponding partial log-likelihood function. SVM with those constraints enables models for high-dimensional omics data to have more flexible structure, e.g. additive (non)-linear models. One distinct feature of SurvivalSVM is that it treats the prognostic problem as a ranking problem and therefore the estimation of the hazards is not directly incorporated in the model.

RSF was first proposed by [21] as an extension of Random Forest to model censored survival data. Random Forest [22] is a non-parametric bagging-based ensemble learning method that adds variation in the training datasets by bootstrapping the data. Multiple models are generated based on many resamples. The ensemble prediction result is then an average of these multiple models or the result of a majority vote. The key components in our application of RSF are that we use Harrell's C-index to evaluate the survival tree instead of the mean square error for regression problems or confusion matrix for classification problems, and that we use the log-rank score in each node as the stopping rule.

Another ensemble-based approach is the boosting method, which contains multiple learners and sequentially gives more weight to weak learners to enhance predictability. For example, the Cox boosting model [23] [24] [25] [26] is developed based on Cox models with boosting being applied to

the estimation of the regression parameter vector β in Equation (1). There are two popular approaches to update β: the first is the model-based approach that leads to the mboost method, the second is the likelihood-based approach that leads to the CoxBoost method (benchmarked in this study).

These models so far only focus on optimising a single objective. Because survival data is time dependent, it is natural to have multiple tasks related to one or more time points of interest. This naturally leads to multi-task learning, a method that deals with the need to predict for more than a single response variable, based on joint optimization of multiple likelihood functions corresponding to each task. The multi-task logistic regression model (MTLR) by Yu et al. [27] is a survival model for multiple time points, where for each the task is to predict survival using a logistic regression model and the parameters from each model are estimated simultaneously in the maximization of the joint likelihood function.

More recently, the ML and artificial intelligence communities refer to the methods described above as classical ML methods due to the emergence of deep learning (DL), a conceptual advancement based on neural networks (NN). In survival analysis, a number of DL survival models were developed such as Cox-nnet [28], DeepSurv [29] and DeepHit [30]. The key concept here is having different loss functions that particularly target either the hazard or the survival probability for those neurons in hidden layers when building the DL architecture. High dimensional complex biological information can be better represented with the application of those hidden layers [31] and through relaxing the proportional hazard assumption.

2.3 Feature selection methods applied to survival models

The input features are fundamental to every statistical or machine learning model, and the survival model is no exception [32]. Wrapper and filter [33] are two feature selection methods that are widely used for not only regression and classification models, but also survival models.

The wrapper approach is a model-dependent method in which the performance of the model determines the selection of subsets of features. Stepwise feature selection approaches fall into this category since one feature is deemed to be included or deleted as the model's performance improves. Other more

computational approach such as the genetic algorithm (GA) [34], which was originally developed to solve an optimization problem, has been extended to use as a feature selection approach [35]. The main idea is to start with an initial set of features to then replace it with one that includes features from other parts of the data to optimize the classification accuracy based on a linear discrimination analysis model. The filtering approach, on the other hand, is a model-independent feature selection method that produces a subset of features without involving the models. This step often occurs outside and before building prediction models. Many of these strategies select features using hypothesis testing statistics from a univariate study. With the advent of omics data in the 1990s, the statistics community embraced the development of differential expression (DE) analysis, which is a filter type feature selection method for identifying promising genes/features using "parallel univariate strategies" based on linear modelling [36].

2.4 Classical performance evaluation metric for survival data

Classically, survival analysis is evaluated in three broad settings: the concordance index, the Brier score and the time-dependent AUC. Similar to evaluating classification and regression models, metrics for calibration and discrimination are developed with incorporating censoring by applying rank-based methods or error based methods together with a weighting scheme.

2.4.1 C-index and its extension in survival analysis

C-indices in survival analysis are concordance-based methods, where 'concordance' measures how close a prediction is to the truth. The original C-index for survival analysis was introduced by Frank. E. Harrell [37], as a time-independent performance measure. C-indices range from 0 to 1, where 1 means perfect performance and 0 means worst possible performance. If a model would not take into account any information from the data, that is a random prediction is made, then the corresponding C-index would be around 0.5. For most clinical datasets, a C-index around or larger than 0.6 is considered an acceptable prediction. Harrell's C-index [38] defines concordance by looking at ranks of pairs of subjects in the data (there are n choose 2 pairs for data with n subjects). Harrell's C index further depends on the censoring distribution of the data, is motivated by Kendall's tau statistic and is closely related to Somers' D. When ranking the subjects, censored subjects are excluded; and pairs included in

the formula are only those comparable, non-censored pairs. There are different versions of the C-index, where the differences come from the different ways that censored subjects are ranked.

We will use the following three concordance indices: Begg's C-index, Uno's C-index and GH C-index. First, Begg's C-index [39] uses KM estimation to incorporate both censored and uncensored subjects by assigning different weight to them. Second, Uno et al [40] develop a new way to calculate the rank with the help of inverse probability of censoring weight (IPCW). Third, the GH C-index [41] changes the concordance function into a probability function based on the Cox model estimation and then approximates its distribution which is robust to censoring.

2.4.2 Brier score

The Brier score [42] [43] uses IPCW to handle censored subjects when measuring discrepancy between the estimated values and the actual values. This score can be considered as a similar measure to the mean squared error (MSE) in regression models to some extent. Like the MSE, the Brier score takes a value greater than 0 that depends on the data and the smaller the Brier score the better. However, to have better interpretability, the integrated Brier score (IBS) is introduced which also takes values between 0 and 1 - it averages the loss over time in situations where there is no interest in a particular time point but performance is with regards to all time points as a whole.

2.4.3 Time-dependent AUC

The time-dependent AUC is inspired from binary classification model evaluations. The receiver operating characteristic (ROC) curve is a classical model assessment plot that examines the relationship between the sensitivity and the false positive rate. The area under the ROC curve is termed AUC (area under the curve). In survival analysis, event statuses are changing over the time, which requires a dynamic measurement to discriminate the predicted versus the actual. Chambless and Diao [44] were the first to propose a time-dependent AUC for survival analysis. They define the AUC(t) as the probability that a person with disease onset by time t has a higher score than the person with no event by time t. Changes of model predictability for different time points can therefore be visualized by time-dependent AUC curves, which allows people to compare long time versus short time predictability.

3. Material and Methods

3.1 Datasets: six clinical and ten omics data sets

Clinical datasets - Six clinical datasets with different sample sizes and disease types are selected (see references in Table 1).

- Veteran data is a survival dataset from the randomised trial of two treatment regimens for lung cancer obtained from the R package "survival". There are 6 measured features in this data.
- Pbc data (5 clinical feature, 312 patients) from the Mayo Clinic trial in primary biliary cirrhosis (Pbc) of the liver conducted between 1974 and 1984; obtained from the R "RandomForestSRC".
- Lung data (7 features, 228 patients) contains patient survival information with advanced lung cancer from the North Central Cancer Treatment Group and is available from the R package "survival.
- ANZ data (ANZDATA): Australia & New Zealand Dialysis and Transplant Registry data containing graft survival information and electronic clinical records for kidney transplantation recipients in Australia and New Zealand from 30th June 2006 to 13th November 2017. This data contains records for both living and deceased donors and also multi-organs transplants. We processed the raw data, restricting the transplant date to be after 2008-09-18 and retained deceased donor kidney transplants only. Missing records are excluded, resulting in 3323 patients and 38 features containing patient, donor and donor-recipient human leukocyte antigen (HLA) compatibility.
- UNOS_Kidney data: Organ transplant data based on the Organ Procurement and Transplantation Network (OPTN)-United Network for Organ Sharing (UNOS) in the US (based on OPTN data as of March, 2020). We selected a random sample of 3000 records associated with deceased donor kidney transplantation only with 99 features containing recipients, donors and donor-recipient HLA compatibility. Missing values are imputed using the R package "MICE".
- Melanoma_clinical data, extracted from melanoma data [45] [46]: A in-house dataset collected as a part of a multi-omics study. This is the part that contains clinical information for patients. After deleting all missing values, we have 88 patients with stage three melanoma disease measured by 14 clinical features.

Omics datasets - We consider eight published data and two in-house melanoma cancer datasets. A summary of the size and censoring rate of all datasets can be found in Table 1.

- Two ovarian cancer gene expression datasets, downloaded from the R package “curatedOvarianData”. Curation and analysis pipeline of this data follow [47]. Ovarian1 is the "GSE49997_eset" data (194 patients/16047) genes, Ovarian2 is the “GSE30161_eset” data (58/19816).
- Another six gene expression datasets are available online from work by Yang and colleagues [48], We have named them GE1 to GE6 for easier rendering of labels in our figures. For GE_3, log2 transformation is applied, followed by a KNN imputation with 10 nearest points. For GE_6, median normalisation is applied. For others, no further pre-processing was performed.
- Melamona_itraq and Melanoma_nano are two in-house melanoma omics datasets [45] [46], the first is a protein expression dataset from the iTRAQplatform and the second is a Nanostring dataset from the above melanoma study and pre-processing steps are described in the respective papers. The itraq protein expression data has 41 patients with 640 proteins. The nanostring data has 45 patients with 204 genes [49], and the GEO ID is “GSE156030".

3.2 Benchmarking design/procedure

Evaluation metrics: We examine model performance metrics that can be broadly grouped into four categories and assess performance in terms of each methods’ flexibility, predictability, stability and computational efficiency detailed in Supp Table 1_Supplementary Material and briefly summarized as follows:

(i) We measure *model flexibility* by looking at whether a given method can handle different data modality, different level of sparsity, and represents multiple ways including the type of data required (clinical, omics), type of input required (categorical, numerical), sparsity of the data allowed (yes, no) and prediction ability evaluation metrics allowed.

(ii) We measure *model predictability* using three different metrics: C-index, time-dependent AUC and Brier score. We apply four different modified versions of C-index: Harrell's C-index, Begg's C-index, Uno's C-index and GH C-index. For identification of different time points, we equally divided the survival time ranging from the 1st quartile to the 3rd quartile into fifteen time points for each dataset, and therefore, we obtained fifteen AUC values corresponding to each time point. As for the Brier score, we calculated the raw Brier score and the IBS. The raw Brier score is calculated by taking the sum of all Brier scores for all event times in the dataset.

(iii) We measure the *model computational efficiency* using both computational time and memory. Computational time is calculated using the "Sys.time" function in R. Memory is calculated using the "Rprof" function in R and the total memory used is summarised for each experiment.

(iv) We measure *model stability* using model reproducibility and the standard deviation (SD) of model predictability metrics. Model reproducibility is defined as the proportion of successful runs among all the runs attempted. For each model predictability metric, we calculated its SD. We then ranked the values for all the methods for each dataset from the most stable (smallest SD) to the least stable (largest SD).

<u>Benchmarking methods:</u> All methods evaluated are described in Table 2 (details in Supplementary Data). In this benchmark study, hyperparameter sets used in these methods are chosen to be the default set. All compared methods (Supplementary Data) and evaluation metrics (Supp Table 1_Supplementary Material) are applied and evaluated on real-world datasets listed in Section 3.1. We apply 20 times (runs) repeated 5-fold cross validation using RStudio server (RRID:SCR_000432) with 15 cores in parallel. For each run, the whole data is split into a training dataset (80%) and a testing dataset (20%) with each method trained using the training dataset and values of evaluation metrics calculated using the testing dataset. For methods with a feature selection step, a nested feature selection step is applied on those training folds within each 5-fold cross validation procedure. Detail about the packages and parameters can be found in (Supplementary Data) and functions used to evaluate the methods are shown in (Supp Table 1_Supplementary Material).

4. Results

4.1 Comprehensive benchmarking design

To comprehensively evaluate the strength and weakness of the survival analysis approaches, we select 20 representative methods from our extensive literature review and study their performance when applied to 16 diverse datasets. The performance of each method is measured against 11 metrics representing multiple aspects, including feasibility, predictability, stability and computational efficiency. There are three key aspects of our comparison design SurvBenchmark as depicted in Figure 1: (i) Practical focus through applying the design to a broad range of datasets and by including a taxonomic methods system that evaluates multiple aspects; (ii) Extensive comparison of methods from classical to state of the art ML approaches; (iii) Comprehensive evaluation of the model performance with the utilisation of a customizable weighting framework (Figure 1b). To apply this in practice, the following steps are needed:

(1) Provide a “weight vector” of length q, where each weight represents the strength for each of the q practical aspects. For example, if an urgent analysis is conducted one may prefer a very high weight for computational time. On the other hand, prediction accuracy may be most important in a situation where computational constraints are of no concern.

(2) According to the specific data modality, select the feasible methods (m in total) and obtain their rank (recorded in a m by q matrix) for the aspects considered in the “weight vector”.

(3) Multiply the rank matrix and the “weight vector” to obtain the final selected method from this list of m scores (Supp Table 3_Supplementary Material).

4.2 Practical consideration in assessing model performance

Many comparison studies define method performance solely in terms of method predictability, with only a few studies taking into account computational time. Often the feasibility of the method is not properly considered or discussed. Practically, it is paramount that a method can be applied to the data at hand, based on both the flexibility (data modality, sparsity) and computational requirement.

Given the diverse collection of data characteristics that is now available in the biomedical field, not all survival approaches are feasible to be applied to all data types. For example, some classical Cox models (Figure 2a, top left from column 1 to 10, row 1 to 4; a blue box indicates ‘method not feasible’), e.g. because it cannot handle large p (features) small n (samples) datasets (such as GE-1) which is a distinct feature of any molecular (omics) study. Advanced feature selection methods together with ML survival models such as CoxBoost(DE) can only take numerical data as the input (purple box for input type, where model characteristics are coded using 0, 1 and 2 with questions defined as below. Is input type numeric only? Yes: numerical only. No: both numerical and categorical are ok. Is output type survival risk? Yes: survival risk. No: survival probability. Can the model handle n<p situation? Yes: it can. No: it cannot. The other case: output is the rank of survival risk.).

Next, we look at the computational aspect, and we notice that DL based methods are computationally inefficient as highlighted by the star icon (Figure 2a). From the many rowwise stars, we observe that RSF (5 stars) and MTLR (5 stars) are not as computationally efficient as Cox-based approaches such as Lasso_Cox (1 star) and CoxBoost (0 stars).

Lastly, a summary tabulating the readily applicability associated with each of the evaluation metrics for prediction is provided in Figure 2b. The results highlight that Begg’s C-index and GH C-index are applicable only for Cox methods (red indicates readily applicability), that the integrated Brier score can be calculated for Cox model and RSF (red), and that the Brier score cannot be calculated for SurvivalSVM (blue).

4.3 Performance evaluation from multiple perspectives: no ‘one size fits all’

To achieve a comprehensive overview of different survival approaches, we assess method performance from multiple perspectives across a large collection of datasets. Here, we color the methods according to their performances for all three broad categories: model predictability, model stability and computational efficiency (Figure 2c shows ranks of those methods where red means the best and blue the worst; similarly, Figure 2d shows Harrell's C-index values with red referring to high values and blue to small values). We find that no method performs optimally across all three categories and there are various trade-offs among the categories.

For model predictability, we use seven different measures based on C-index, Brier score and time-dependent AUC. Here, MTLR-based approaches perform evidently better than others, which is most apparent by looking at the performance results using C-index and time-dependent AUC. In order to further examine whether MTLR-based approaches have similar performance across all datasets, we show our examination on one specific criteria (the most popular Harrell's C-index; Mean_hc). In Figure 2d we demonstrate that MTLR has optimal performance for all but one of the six clinical datasets with PBC having optimal performance for one of the clinical datasets. Variants of MTLR (MTLR(GA) and MTLR(DE)) outperformed MTLR when applied to any of the ten omics datasets suggesting the performance of the approaches depend on the type of dataset.

For computational efficiency as measured by computational time and memory usage, the best performing methods are classical Cox-based models and CoxBoost. In particular, Cox, Cox_bw_AIC and Cox_bw_BIC are the top three performing methods for computational time (Figure 2c). For model stability, we have seven criteria and they are based on calculating the standard deviation (SD) of predictability metrics described above. Similar to the the computational efficiency performance, when using SD-criteria, Cox, Cox_bw_AIC and Cox_bw_BIC are also the top 3 performing methods in all but one criteria, the exception is the standard deviation of Brier score (SD_bs), where DNNSurv ranks first suggesting its ability to discriminate survival probabilities for different observations.

In conclusion, the above observations demonstrate that no method performs optimally for all those categories. In practice, we recommend first completing a feasibility check first to draw conclusions on time constraints and to heighten awareness of the data types actually present, and then explicitly deciding on the focus of the research, for example that model predictability is the top priority. Our analysis supports the use of MTLR and its variants for both omics and clinical datasets when survival prediction is the key priority, despite the fact that these approaches are inefficient [50]. However, Cox-based models are preferable when comparing the effect of variables, such as the treatment effect for clinical datasets, because of their efficiency and interpretability.

4.4 Cox-based modern ML methods have similar prediction performance compared to classical Cox-based methods

To understand the gain in model predictability from Cox-based modern ML methods (CoxBoost, Coxboost (GA)), we compare these models with classical Cox-based methods (Lasso_Cox, EN_Cox) which are used as a gold standard method in many studies. Our results indicate that they have similar performance (Figure 3) across a large collection of datasets. For example, in the ANZ data, which is a representative clinical dataset, we observe similar model predictability measured by both Harrell's C-index and Brier score. For GE_5, a representative dataset of omics with large p small n data characteristics, the same conclusion is drawn. This suggests the performance of modern ML methods in complex health and clinical data is not as clear cut as in some other domains.

4.5 Data dependent model performance for different time

To study the model performance over time, we visualize this using the time-dependent AUC curves for all methods. Here we observe among two representative clinical datasets (PBC, UNOS_Kidney) and two omics datasets (GE_2, GE_4) in Figure 4, not all curves are parallel to each other, indicating that the behaviour of model predictability for different time points is data dependent (see Supp Figure 1_Supplementary Material for further results).

We pick two representative models (Cox(GA) & RSF) to demonstrate this data dependent model behaviour. For UNOS_Kidney data and GE_2 data, the curves are approximately horizontal, which indicates the consistency of short-time, medium time and long-time model predictability. In contrast, for PBC data and GE_4 data, model predictability changes along those time points.

5. Conclusions

This benchmark study comprehensively evaluated the relevance and usefulness of survival models in practice, where emphasis is on performance over diverse datasets. In our review we assessed a broad variety of survival methods from classical Cox-PH models to modern ML models. In this study we did not survey the extensive tuning procedures for those survival approaches. The main reason being that, in practice, often the default hyperparameter sets are used. Therefore, we also decided to use the default sets in our study here. However, we note that applying targeted tuning methods for a particular dataset may lead to different performances for the considered approaches. The findings of our systematic

assessment will provide specific guidance for translational scientists and clinicians, as well as define areas of potential study in both survival methodology and benchmarking strategies.

In recent years, there is a clear shift in how survival data is analysed, from modelling directly the hazard function to building models directly on survival functions. Conceptually, modelling hazard functions is a good way to identify key risk factors related to various patients' risk levels. On the other hand, if our key criterion is to predict accurately survival, modelling survival probability directly improves predictability. Methods including MTLR, DNNSurv and SurvivalSVM which directly model the survival function showed better performance in terms of model predictability, this is consistent with what [27] have commented on when discussing the performance of their proposed MTLR method.

It is striking that MTLR shows remarkably high model predictability in our benchmark study. We now highlight technical advantages, disadvantages as well as its applications. Numerous reasons could contribute to the better model prediction performance of the MTLR-based approaches. These include the three main reasons as discussed by [27]: direct modeling of the survival function, simultaneous building of multiple logistic regression models, and dynamic modeling. Interestingly, the majority of extended MTLR models since 2011 are based on neural networks as researchers extend the concept to account for nonlinearity in datasets [51]. To date, only a limited number of studies have applied MTLR in health using clinical data in HIV patients [52] or on large omics datasets to predict patient survival in breast and kidney cancers [53]. Given its outstanding model predictability observed for most of the datasets in our study, we believe there is opportunity to use MTLR more widely for survival risk modelling in Health contexts.

Model predictability is one of the key metrics to assess survival studies with Harrell's C-index being currently the most popular. As this kind of ranking based concordance measurement is suitable to evaluate predicted outcomes with censored data, various concordance indices are developed using different methods to handle censoring such as Uno's C-index using IPCW. Besides concordance indices, other predictability metrics such as the time-dependent AUC, which applies a similar idea as the AUC in binary classification but divides the whole time interval into multiple time points, are also adopted in some survival studies [54]. Given that model predictability could be measured by multiple

types of indices, we suggest that hybrid evaluation metrics should be applied in practice to provide relatively comprehensive assessments for the fitted model.

While many survival approaches are applicable to both clinical and omics data, there are a number of recently developed approaches that are specifically tailored for high dimensional omics data, such as CoxBoost. The rationale behind developing data-specific methods is to better capture the distinct data characteristics in either the clinical or omics studies. Clinical data usually include mixed modality variables, large sample sizes but have large n (observations) and small p (features). In contrast, omics data naturally comes with a large collection of molecular features and with small n but their data type is homogenous. When it comes to various real-world datasets, performances are also affected by many other aspects besides data type (clinical, omics) such as data modality and therefore, it is challenging to directly examine whether those tailored methods indeed improve the performance. Further examination of the aspects that affect model predictability can be found in Supp Table 2_Supplementary Material.

Deep learning based methods failed for some datasets on some cross-validation runs. Taking the method DNNSurv as an example, among all 100 runs, DNNSurv had a 100% completion rate for 5 out of the 12 applicable datasets (Supp Figure 2_Supplementary Material) only. For the remaining 7 datasets, completion rate was around 80% and as low as 63% for the Melanoma_itraq data. This instability is likely due tuning parameter sensitivity when sample size is small [55]. All failed iterations are not recorded when generating the results.

Data Availability

For the ANZDATA, data request can be made through the ANDATA registry, and access to the data source will require HREC approvals. For the UNOS_kidney data, it can be requested from [56] .

Codes for running those methods and evaluation measurements for an example dataset is available at [57]. All supporting data and materials are available in the *GigaScience* GigaDB database [58].

Availability of supporting source code and requirements

Project name: SurvBenchmark

Project home page: https://github.com/SydneyBioX/SurvBenchmark_package

Operating system(s): Platform independent

Programming language: R

Other requirements: R studio

License: Apache 2.0

RRID:SCR_022503

Funding

The following sources of funding for each author, and for the manuscript preparation, are gratefully acknowledged: Australian Research Council Discovery Project grant (DP170100654) to JYHY and SM, Australian Research Council Discovery Project grant (DP210100521) to SM, AIR@innoHK programme of the Innovation and Technology Commission of Hong Kong to JYHY. Research Training Program Tuition Fee Offset and Stipend Scholarship and the Dean's International Postgraduate Research Scholarship (DIPRS) to YZ. The funding source had no role in the study design; in the collection, analysis, and interpretation of data, in the writing of the manuscript, and in the decision to submit the manuscript for publication.

Declarations

Competing interests

The authors declare that they have no competing interests.

Authors' contributions

JYHY and SM conceived, designed and funded the study with guidance from GW and GM. GM and GW provided access to in-house data, and jointly develop the problem formulation of the study with JYHY and SM. YZ developed the benchmarking design, implemented all the models in R and the

evaluation design with guidance from JYHY and SM. YZ, JYHY and SM wrote the manuscript and all authors read and approved the final version of the manuscript.

Acknowledgements

The authors thank all their colleagues, particularly at The University of Sydney, Sydney Precision Bioinformatics Alliance and Charles Perkins Centre for their support and intellectual engagement.

References

1. Thrane C. Analyzing tourists' length of stay at destinations with survival models: A constructive critique based on a case study. Tourism Management. 2012. p. 126–32.

2. Ancarani A, Di Mauro C, Fratocchi L, Orzes G, Sartor M. Prior to reshoring: A duration analysis of foreign manufacturing ventures. International Journal of Production Economics. 2015. p. 141–55.

3. Esmalian A, Dong S, Mostafavi A. Susceptibility curves for humans: Empirical survival models for determining household-level disturbances from hazards-induced infrastructure service disruptions. Sustainable Cities and Society. 2021. p. 102694.

4. Schober,P. and Vetter,T.R. Survival Analysis and Interpretation of Time-to-Event Data. Anesth. Analg. 2018a; 127, 792–798;

5. Wang P, Li Y, Reddy CK. Machine learning for survival analysis. *ACM Comput Surv*. Association for Computing Machinery (ACM) 2019; 51:1–36;

6. Schober,P. and Vetter,T.R. Survival Analysis and Interpretation of Time-to-Event Data: The Tortoise and the Hare. Anesth. Analg. 2018b; 127, 792–798;

7. Ahmed FE, Vos PW, Holbert D. Modeling survival in colon cancer: a methodological review. *Mol Cancer*. Springer Nature2007; 6:15;

8. Lee S, Lim H. Review of statistical methods for survival analysis using genomic data. *Genomics Inform*. Korea Genome Organization2019; 17:e41;

9. Herrmann M, Probst P, Hornung R, Jurinovic V, Boulesteix A-L. Large-scale benchmark study of survival prediction methods using multi-omics data. *Brief Bioinform*. Oxford University Press (OUP); 2021; doi: 10.1093/bib/bbaa167.

10. Cox DR. Regression models and life- tables. Journal of the Royal Statistical Society: Series B (Methodological). 1972 Jan;34(2):187-202.

11. Tibshirani R. The lasso method for variable selection in the Cox model. *Stat Med*. Wiley1997; 16:385–95;

12. Van Houwelingen HC. The elements of statistical learning, data mining, inference, and prediction. Trevor Hastie, Robert Tibshirani and Jerome Friedman, Springer, New York, 2001. No. Of pages: Xvi+533. ISBN 0-387-95284-5. *Stat Med*. Wiley2004; 23:528–9;

13. Do KA, Qin ZS, Vannucci M, editors. Advances in statistical bioinformatics: models and integrative inference for high-throughput data. Cambridge University Press; 2013. https://doi.org/10.1017/CBO9781139226448

14. Huang H-H, Liang Y. Hybrid L1/2 + 2 method for gene selection in the Cox proportional hazards model. *Comput Methods Programs Biomed*. Elsevier BV2018; 164:65–73;

15. Gogas P, Papadimitriou T. Machine learning in economics and finance. *Comput Econ*. Springer Science and Business Media LLC; 2021; doi: 10.1007/s10614-021-10094-w.

16. Chen W, Xie X, Wang J, Pradhan B, Hong H, Bui DT, et al.. A comparative study of logistic model tree, random forest, and classification and regression tree models for spatial prediction of landslide susceptibility. *Catena*. Elsevier BV2017; 151:147–60;

17. Lakshmanaprabu SK, Shankar K, Ilayaraja M, Nasir AW, Vijayakumar V, Chilamkurti N. Random forest for big data classification in the internet of things using optimal features. *Int j mach learn cybern*. Springer Science and Business Media LLC2019; 10:2609–18;

18. Dietrich S, Floegel A, Troll M, Kühn T, Rathmann W, Peters A, et al.. Random Survival Forest in practice: a method for modelling complex metabolomics data in time to event analysis. *Int J Epidemiol* 2016. 45:1406–20;

19. Van Belle V, Pelckmans K, Van Huffel S, Suykens JAK. Improved performance on high-dimensional survival data by application of Survival-SVM. *Bioinformatics*. Oxford University Press (OUP) 2011; 27:87–94;

20. Van Belle, Vanya, Kristiaan Pelckmans, Johan AK Suykens, and Sabine Van Huffel. "Survival SVM: a practical scalable algorithm." In ESANN, vol. 89, p. 94. 2008.

21. Ishwaran H, Kogalur UB, Blackstone EH, Lauer MS. Random survival forests. *Ann Appl Stat*. Institute of Mathematical Statistics 2008; 2:841–60;

22. Breiman, L. Random Forests. *Machine Learning*. 2001; doi: 10.1023/A:1010933404324;

23. Bin RD, De Bin R. Boosting in Cox regression: a comparison between the likelihood-based and the model-based approaches with focus on the R-packages CoxBoost and mboost. *Comput Statist* 2016. 31:513–531;

24. Binder H, Allignol A, Schumacher M, Beyersmann J. Boosting for high-dimensional time-to-event data with competing risks. *Bioinformatics*. Oxford University Press (OUP) 2009; 25:890–6;

25. Binder H, Benner A, Bullinger L, Schumacher M. Tailoring sparse multivariable regression techniques for prognostic single-nucleotide polymorphism signatures. *Stat Med*. Wiley 2013; 32:1778–91;

26. Binder H, Schumacher M. Incorporating pathway information into boosting estimation of high-dimensional risk prediction models. *BMC Bioinformatics*. Springer Nature 2009; 10:18;

27. Yu CN, Greiner R, Lin HC, Baracos V. Learning patient-specific cancer survival distributions as a sequence of dependent regressors. Advances in neural information processing systems. 2011;24.

28. Ching T, Zhu X, Garmire LX. Cox-nnet: An artificial neural network method for prognosis prediction of high-throughput omics data. *PLoS Comput Biol* 2018. 14:e1006076;

29. Katzman JL, Shaham U, Cloninger A, Bates J, Jiang T, Kluger Y. DeepSurv: personalized treatment recommender system using a Cox proportional hazards deep neural network. *BMC Med Res Methodol*. Springer Science and Business Media LLC; 2018; doi: 10.1186/s12874-018-0482-1.

30. Ryu JY, Lee MY, Lee JH, Lee BH, Oh K-S. DeepHIT: a deep learning framework for prediction of hERG-induced cardiotoxicity. *Bioinformatics*. Oxford University Press (OUP) 2020; 36:3049–55;

31. Ching T, Himmelstein DS, Beaulieu-Jones BK, Kalinin AA, Do BT, Way GP, et al.. Opportunities and obstacles for deep learning in biology and medicine. *J R Soc Interface*. The Royal Society 2018; 15:20170387;

32. Heinze G, Wallisch C, Dunkler D. Variable selection - A review and recommendations for the practicing statistician. *Biom J*. Wiley 2018; 60:431–49;

33. Bagherzadeh-Khiabani F, Ramezankhani A, Azizi F, Hadaegh F, Steyerberg EW, Khalili D. A tutorial on variable selection for clinical prediction models: feature selection methods in data mining could improve the results. *J Clin Epidemiol* 2016. 71:76–85;

34. Holland JH. Adaptation in Natural and Artificial Systems: An Introductory Analysis with Applications to Biology. Control, and Artificial Intelligence MIT Press;1992;

35. Saeys Y, Inza I, Larrañaga P. A review of feature selection techniques in bioinformatics. *Bioinformatics*. Oxford University Press (OUP) 2007; 23:2507–17;

36. Bommert A, Sun X, Bischl B, Rahnenführer J, Lang M. Benchmark for filter methods for feature selection in high-dimensional classification data. *Comput Stat Data Anal*. Elsevier BV 2020; 143:106839;

37. Harrell FE. Evaluating the yield of medical tests. *JAMA*. American Medical Association (AMA) 1982; 247:2543–6;

38. Newson R. Confidence intervals for rank statistics: Somers' D and extensions. *Stata J*. 2006; 6:309–34;

39. Begg BC, Craemer LD, Venkatraman ES, Rosai J. Comparing tumor staging and grading systems: a case study and a review of the issues, using thymoma as a model. *Statistics in Medicine* 2000. 19:1997–2014;

40. Uno H, Cai T, Pencina MJ, D'Agostino RB, Wei LJ. On the C-statistics for evaluating overall adequacy of risk prediction procedures with censored survival data. *Stat Med*. Wiley 2011; 30:1105–17;

41. Gönen M, Heller G. Concordance probability and discriminatory power in proportional hazards regression. *Biometrika*. Oxford University Press (OUP) 2005; 92:965–70;

42. Gerds TA, Schumacher M. Consistent estimation of the expected Brier score in general survival models with right-censored event times. *Biom J*. Wiley 2006; 48:1029–40;

43. Schmid M, Hielscher T, Augustin T, Gefeller O. A robust alternative to the schemper-henderson estimator of prediction error. *Biometrics*. Wiley 2011; 67:524–35;

44. Chambless LE, Diao G. Estimation of time-dependent area under the ROC curve for long-term risk prediction. *Stat Med*. Wiley 2006; 25:3474–86;

45. Mactier S, Kaufman KL, Wang P, Crossett B, Pupo GM, Kohnke PL, et al.. Protein signatures correspond to survival outcomes of AJCC stage III melanoma patients. *Pigment Cell Melanoma Res*. Wiley 2014; 27:1106–16;

46. Mann GJ, Pupo GM, Campain AE, Carter CD, Schramm S-J, Pianova S, et al.. BRAF mutation, NRAS mutation, and the absence of an immune-related expressed gene profile predict poor outcome in patients with stage III melanoma. *J Invest Dermatol*. Elsevier BV 2013; 133:509–17;

47. Ganzfried BF, Riester M, Haibe-Kains B, Risch T, Tyekucheva S, Jazic I, et al.. curatedOvarianData: clinically annotated data for the ovarian cancer transcriptome. *Database (Oxford)*. Oxford University Press (OUP) 2013; 2013:bat013;

48. Yang L, Pelckmans K. Machine learning approaches to survival analysis: Case studies in microarray for breast cancer. *Int J Mach Learn Comput*. EJournal Publishing 2014; 4:483–90;

49. Wang KYX, Pupo GM, Tembe V, Patrick E, Strbenac D, Schramm S-J, et al.. Cross-Platform Omics Prediction procedure: a game changer for implementing precision medicine in patients with stage-III melanoma. bioRxiv. bioRxiv;2020.12.09.415927;

50. He K, Sun J. Convolutional neural networks at constrained time cost. *2015 IEEE Conference on Computer Vision and Pattern Recognition (CVPR)*. IEEE;

51. Fotso S. Deep neural networks for survival analysis based on a Multi-task framework. arXiv 2018. 1801.05512;

52. Bisaso KR, Karungi SA, Kiragga A, Mukonzo JK, Castelnuovo B. A comparative study of logistic regression based machine learning techniques for prediction of early virological suppression in antiretroviral initiating HIV patients. *BMC Med Inform Decis Mak*. Springer Science and Business Media LLC 2018; 18:77;

53. Wang L. Multi-task survival analysis. 2017 IEEE International Conference on Data Mining (ICDM), IEEE, 2017.p. 485–494;

54. Li G, Chen J-Z, Chen S, Lin S-Z, Pan W, Meng Z-W, et al.. Development and validation of novel nomograms for predicting the survival of patients after surgical resection of pancreatic ductal adenocarcinoma. *Cancer Med*. Wiley 2020; 9:3353–70;

55. Shaikhina T, Khovanova NA. Handling limited datasets with neural networks in medical applications: A small-data approach. *Artif Intell Med* 2017. 75:51–63;

56. Organ Procurement and Transplantation Network. https://optn.transplant.hrsa.gov/data/

57. SurvBenchmark. GitHub. https://github.com/SydneyBioX/SurvBenchmark

58. Zhang Y; Wong G; Mann G; Muller S; Yang JYH (2022): Supporting data for "SurvBenchmark: comprehensive benchmarking study of survival analysis methods using both omics data and clinical data" GigaScience Database. http://dx.doi.org/10.5524/102228.

Figures

Figure 1: SurvBenchmark--Schematic view of our benchmark design.

(a) An overview of survival methods used in this study. We broadly classify current models into two categories; classical statistical models (top group) and modern machine learning models (bottom group) which is inspired by the study from Wang et al [2]. Each of these categories can be further subdivided as presented in the hierarchical chart. All models in blue and red colored boxes are implemented in this current benchmark study. (b) A graphical representation of the SurvBenchmark

design. The methods and evaluation metrics are summarised in a matrix with a flexible user defined weights vector.

Figure 2: Summary heatmaps.

(a) Summary for method flexibility and computational efficiency. Row: methods; Column: datasets; Dendrogram: similarity among datasets; Legend: (1) Left panel indicators 0, 1 and 2, where 0 represents “no” , 1 represents “yes” and 2 represents “the other case” for the corresponding questions listed here. Is input type numeric only? Yes: numerical only. No: both numerical and categorical are ok. Is output type survival risk? Yes: survival risk. No: survival probability. Can the model handle n<p situation? Yes: it can. No: it cannot. The other case: output is the rank of survival risk. (2) feasibility where red (1) means feasible and blue (0) means not feasible. (3) main indicators including datasets characteristics by different colours and stars represent the model is both memory and time consuming. Similarity is defined using the Euclidean distance with feasibility indicator 0 and 1. (b) Prediction ability evaluation metric flexibility. Row: methods; Column: prediction ability evaluation metrics; Dendrogram: similarity among evaluation metrics; Legend: readily applicability where red (1) means readily applicable and blue (0) means not readily applicable. Similarity is defined using the Euclidean distance with feasibility indicator 0 and 1. (c) Rank heatmap for method overall performance. Row: methods; Column: performance metrics; Legend: (1) Rank: red to blue from 1 to 20 where 1 means the top rank. (2) Performance metric categories: 5 different categories representing all metrics used to evaluate method performances. (d) Harrell’s C-index heatmap. Row: datasets; Column: methods; Legend: Harrell’s C-index.

Figure 3: Prediction ability for cox-based methods.

Top left: Harrell’s C-index on ANZ data. Top right: Brier score on ANZ data. Bottom left: Harrell’s C-index on GE_5. Bottom right: Brier score on GE_5.

Figure 4: Time-dependent AUC curves.

(a) PBC data (b) UNOS_US data (c) GE_4 data (d): GE_2 data. Two selected models: Cox(GA), RSF

Table1. Datasets summary

**Datasets summary**					
**Dataset (name usedin this paper)**	**Number of observations**	**No. of variables**	**Type of data**	**Censoring rate (rounded to 4 decimal places)**	**Reference**
Melanoma_itraq	41	642	Omics	0.4146	Wang,K.Y.X. et al. Cross-Platform Omics Prediction procedure: a game changer for implementing precision medicine in patients with stage-III melanoma.bioRxiv 2020.12.09.415927; doi: https://doi.org/10.1101/2020.12.09.415927
Melanoma_nano	45	206	Omics	0.4222	Wang,K.Y.X. et al. Cross-Platform Omics Prediction procedure: a game changer for implementing precision medicine in patients with stage-III melanoma.bioRxiv 2020.12.09.415927; doi: https://doi.org/10.1101/2020.12.09.415927
Ovarian_2	58	19818	Omics	0.3793	Ganzfried,B.F. et al. (2013) curatedOvarianData: clinically annotateddata for the ovarian cancer transcriptome. Database, 2013.
GE_5	78	4753	Omics	0.5641	van ’t Veer,L.J. et al. (2002) Gene expression profiling predictsclinical outcome of breast cancer. Nature, 415, 530–536.
GE_3	86	6288	Omics	0.7209	Bullinger,L. et al. (2004) Use of Gene-Expression Profiling to IdentifyPrognostic Subclasses in Adult Acute Myeloid Leukemia. New England Journal of Medicine, 350, 1605–1616.
Melanoma_clinical	88	16	Clinical	0.3939	Wang,K.Y.X. et al. Cross-Platform Omics Prediction procedure: a game changer for implementing precision medicine in patients with stage-III melanoma.bioRxiv 2020.12.09.415927; doi: https://doi.org/10.1101/2020.12.09.415927.
GE_1	115	551	Omics	0.6670	Sorlie,T. et al. (2003) Repeated observation of breast tumor subtypesin independent gene expression data sets. Proc. Natl. Acad. Sci. U. S. A., 100, 8418–8423.
GE-_4	116	4753	Omics	0.5641	van de Vijver,M.J. et al. (2002) A gene-expression signature as a predictor of survival in breast cancer. N. Engl. J. Med., 347, 1999–2009.
Veteran	137	8	Clinical	0.0657	Kalbfleisch,J.D. and Prentice,R.L. (2002) The Statistical Analysis ofFailure Time Data. Wiley Series in Probability and Statistics.
Ovarian_1	194	16050	Omics	0.7062	Ganzfried,B.F. et al. (2013) curatedOvarianData: clinically annotateddata for the ovarian cancer transcriptome. Database, 2013.
Lung	228	9	Clinical	0.2763	Loprinzi,C.L. et al. (1994) Prospective evaluation of prognostic variables from patient-completed questionnaires. North Central Cancer Treatment Group. J. Clin. Oncol., 12, 601–607.

GE_6	240	7401	Omics	0.4250	Van Houwelingen,H.C. (2004) The Elements of Statistical Learning, Data Mining, Inference, and Prediction. Trevor Hastie, Robert Tibshirani and Jerome Friedman, Springer, New York, 2001. No. of pages: xvi 533. ISBN 0-387-95284-5. Statistics in Medicine, 23, 528–529.
GE_2	295	4921	Omics	0.7322	Beer,D.G. et al. (2002) Gene-expression profiles predict survival of patients with lung adenocarcinoma. Nat. Med., 8, 816–824.
PBC	312	7	Clinical	0.5994	Fleming,T.R. and Harrington,D.P. (2005) Counting Processes and Survival Analysis. Wiley Series in Probability and Statistics.
UNOS_Kidney	3000	101	Clinical	0.7350	OPTN data (https://optn.transplant.hrsa.gov/)
ANZ	3323	40	Clinical	0.8739	ANZDATA (https://www.anzdata.org.au/)

Data table showing the names of datasets used in this paper in the first column. Datasets are ordered by the number of observations (second column, from smallest to largest). Censoring rate is rounded to 4 decimal places.

Table2. Summary of methods used in this study

**Method name**	**Method name in this paper**	**R function name**	**R package name**	**Parameters (default)**
Cox	Cox	coxph	survival	NA
Cox with backward elimination using AIC	Cox_bw_AIC	cph, fastbw	rms	rule="aic", sls=.05,k.aic=2
Cox with backward elimination using p value	Cox_bw_p	cph, fastbw	rms	rule="p", sls=.05
Cox with backward elimination using BIC	Cox_bw_BIC	cph, fastbw	rms	rule="aic",sls=.05,k.aic = log(as.numeric(table(train$status)[2]))

Lasso cox (for clinical datasets)	Lasso_Cox	penalized	penalized	Lambda1=1, lambda2=0
Ridge cox (for clinical datasets)	Ridge_Cox	penalized	penalized	Lambda1=0, lambda2=1
Elastic net cox (for clinical datasets)	EN_Cox	penalized	penalized	Lambda1=1, lambda2=1
Lasso cox (for omics datasets)	Lasso_Cox	glmnet	glmnet	alpha=1, nfolds = 5,type.measure = "C"
Ridge cox (for omics datasets)	Ridge_Cox	glmnet	glmnet	alpha=0, nfolds = 5,type.measure = "C"
Elastic net cox (for omics datasets)	EN_Cox	glmnet	glmnet	alpha=0.5, nfolds = 5,type.measure = "C"
Random survival forest	RSF	rfsrc	RandomSurvivalForest	Default:ntree = 1000,mtry = 10
Multi task logistic regression method	MTLR	mtlr	MTLR	C1=1
DNNSurv (Deep learning survival model)	DNNSurv	multiple functions as in Github codes	DNNSurv	Default: no parameter arguments to be changed by users
Boosting cox model	CoxBoost	coxboost	CoxBoost	stepnumber=10, penalty number=100
Cox model with genetic algorithm as feature selection method	Cox (GA)	GenAlg	GenAlgo	n.features=10 (for omics) , n.features=4 (for clinical) , generation_num=20
Multi task logistic regression model with genetic algorithm as feature selection method	MTLR(GA)	GenAlg	GenAlgo	n.features=10 (for omics) , n.features=4 (for clinical) ,generation_num=20
Boosting cox model with genetic algorithm as feature selection method	CoxBoost (GA)	GenAlg	GenAlgo	n.features=10 (for omics) , n.features=4 (for clinical) , generation_num=20

Multi task logistic regression model with ranking based method as feature selection meth	MTLR(DE)	lmFit,eBaye s	limma	n.features=10 (for omics) , n.features=4 (for clinical)
Boosting cox model with ranking based method as feature selection method	CoxBoost (DE)	lmFit,eBaye s	limma	n.features=10 (for omics) , n.features=4 (for clinical)
Survival support vector machine	SurvivalSVM	survivalsvm	survivalsvm	Default: sgf.sv = 5, sigf = 7, maxiter = 20, margin = 0.05, bound = 10, eig.tol = 1e-06, conv.tol = 1e-07, posd.tol = 1e-08
DeepSurv (Deep learning survival model)	DeepSurv	deepsurv	survivalmo dels	Default: frac = 0.3, activation = "relu", num_nodes = c(4L, 8L, 4L, 2L), dropout = 0.1, early_stopping = TRUE, epochs = 100L, batch_size = 32L
DeepHit (Deep learning survival model)	DeepHit	deephit	survivalmod els	Default: frac = 0.3, activation = "relu", num_nodes = c(4L, 8L, 4L, 2L), dropout = 0.1, early_stopping = TRUE, epochs = 100L, batch_size = 32L

Data table showing the methods used in this benchmark study. R packages and functions with parameters are listed.

Figure1

Click here to access/download;Figure;figure1all.pdf

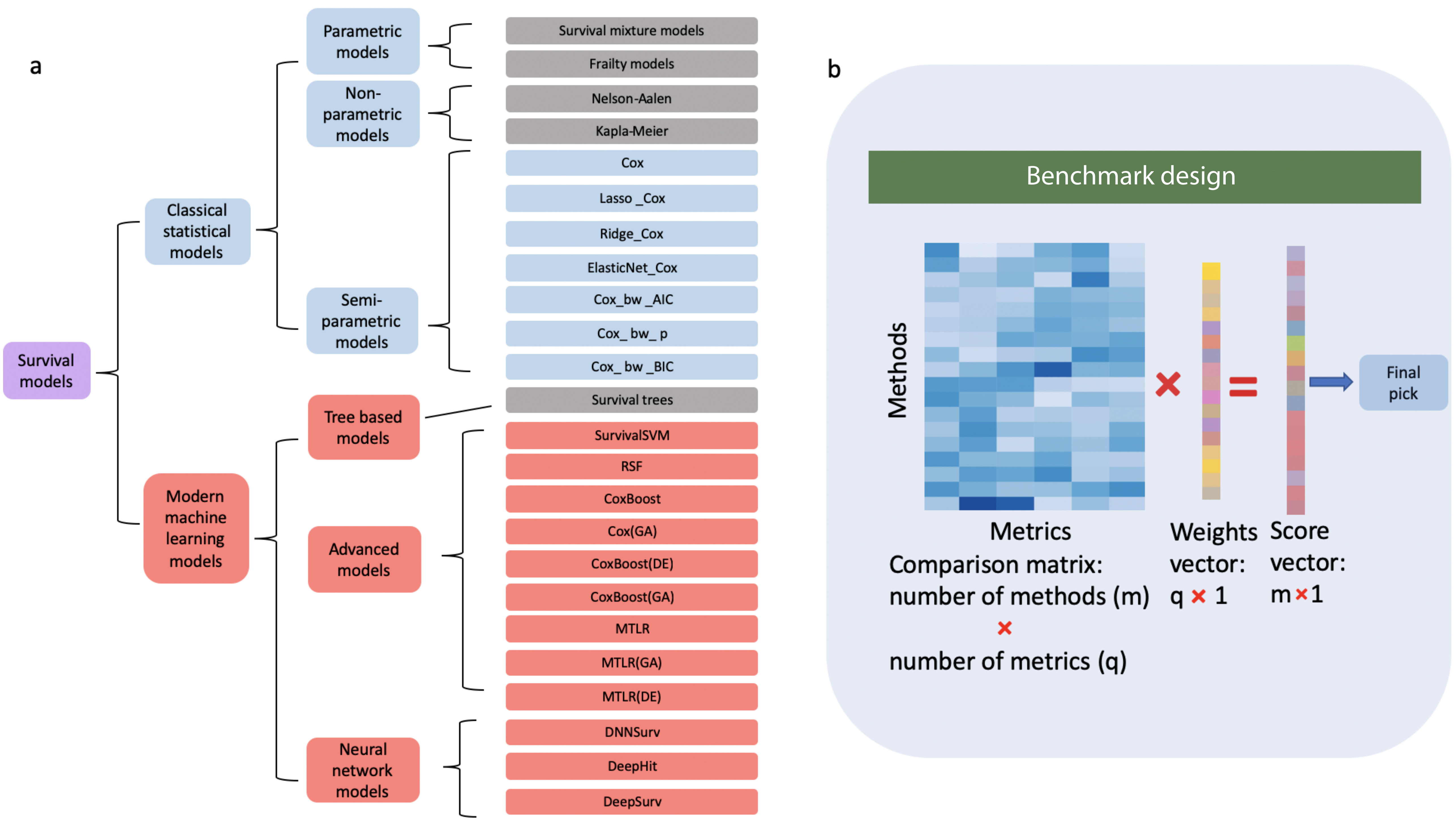

Click here to access/download;Figure;figure2.pdf

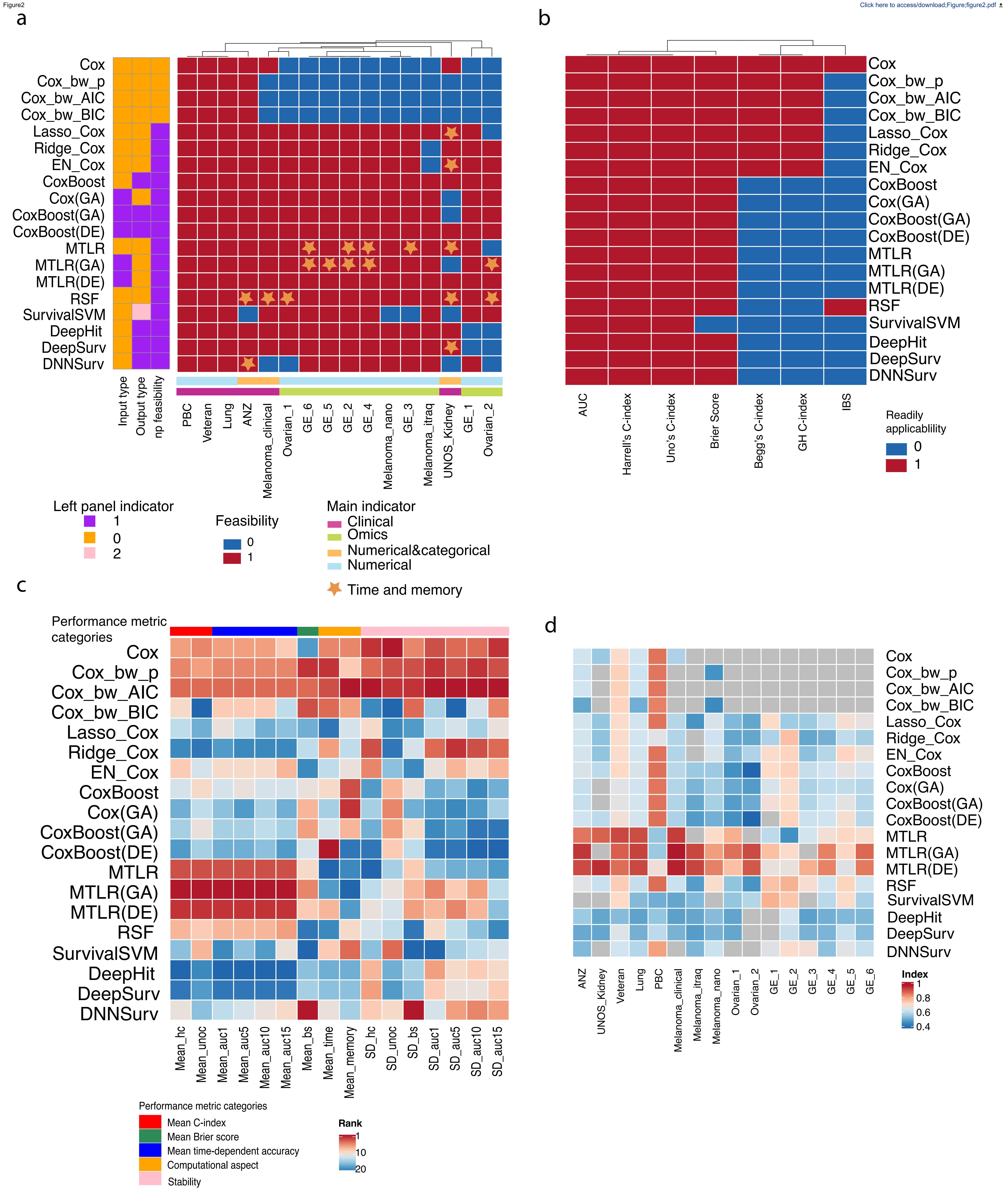

Click here to access/download;Figure;figure3.pdf

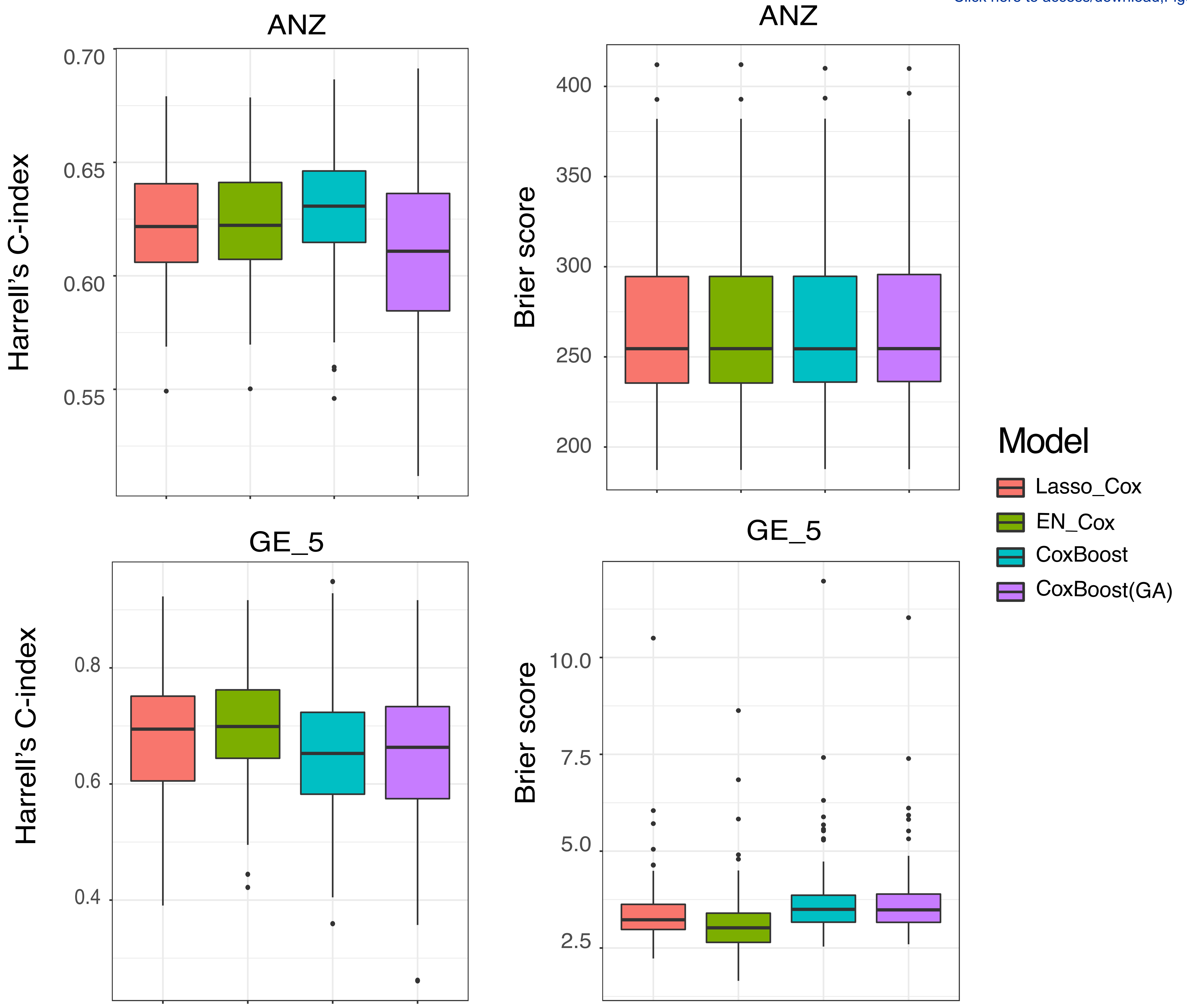

Click here to access/download;Figure;figure4.pdf

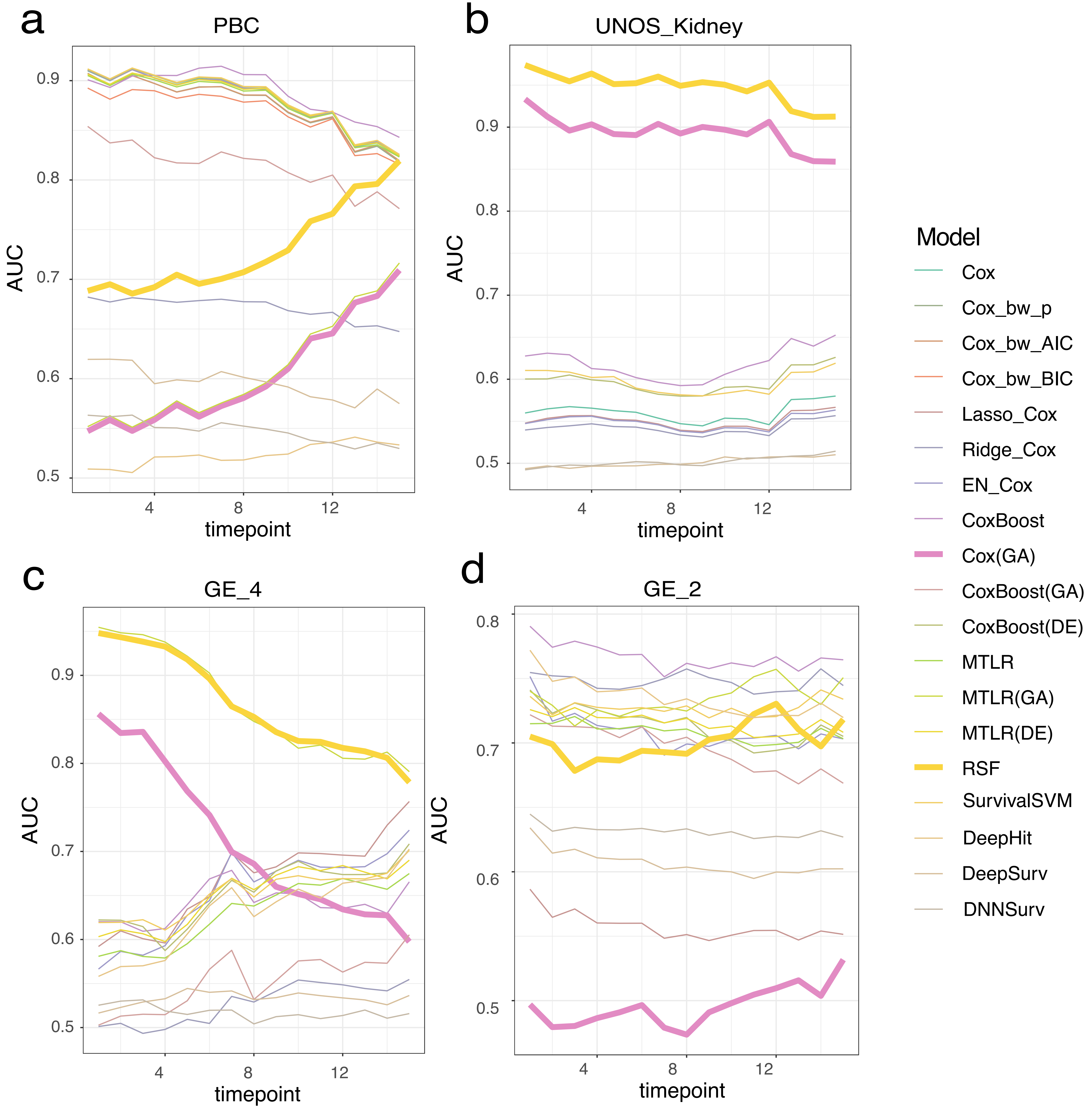

Click here to access/download
Supplementary Material
Supplementary Material_20220513.pdf

Supplementary Data

Click here to access/download
Supplementary Material
Supplymentary Data.xlsx

Cover letter and response Letter
Click here to access/download;Personal Cover;YW-Survbenchmark-cover.pdf

THE UNIVERSITY OF SYDNEY

Hongling Zhou
Associate Editor
GigaScience

16 May 2022

Dear Dr. Zhou

Thank you for inviting us to submit a revised manuscript "SurvBenchmark: comprehensive benchmarking study of survival analysis methods using both omics data and clinical data" by Zhang et al in GigaScience. We thank you for your consideration of our manuscript numbered GIGA-D-22-00036 and thank the reviewers for their constructive feedback. Based on these, we have addressed all remarks and improved the manuscript. Some important points raised by the referees led to more precise terminology in the revised manuscript. The two main modifications in the revised manuscript are that: :

- we have added discussion points addressing that the performance may depend on the method of tuning;
- we have added additional boxplots that more comprehensively show model performances.

Yours sincerely,

Jean Yee Hwa Yang (on behalf of all authors)

Reviewer reports:
Reviewer #2: Many thanks for the very careful revision of the manuscript. Most of my concerns have been thoroughly addressed. I have only a few remarks left.

Regarding 1. Fair comparison and parameter selection
The altered study design appears much better suited to this end. Thank you very much for the effort, in particular the additional results regarding the two tuning approaches. Although I think a single simple tuning regime would be feasible here, using the default settings is reasonable and very well justified. I agree that this is much closer to what is likely to take place in practice. However, it should be more clearly emphasized that better performance may be achievable if tuning is performed.

Response:
Thank you for this comment. We agree that it is important to inform readers that suitable tuning may achieve better performance for a particular method.

We now add to line 414 the following:
"In this study we did not survey the extensive tuning procedures for those survival approaches. The main reason being that, in practice, often the default hyperparameter sets are used. Therefore, we also decided to use the default sets in our study here. However, we note that applying targeted tuning methods for a particular dataset may lead to different performances for the considered approaches. "

Regarding 2. Description
Thanks, all concerns properly addressed. No more comments.

Regarding 3. Reliability
I am aware that Figure 2c provides information to this end. I think additional boxplots which aggregate the methods' performance (e.g. for unoc and bs) over all runs and datasets would provide valuable additional information. For example, from Figure 2c one can tell that MTLR variants obtain overall higher ranks based on mean prediction performance than the deep learning methods. However, it says nothing about how large the differences in mean performance are.

Response:
Thank you for this comment. We agree that boxplots will provide complementary information especially on both the variation and the mean. In our Figure 2c, stability ranking (SD_unoc, SD_bs) measures the variation, i.e. the differences in mean performances. We have chosen to illustrate the information in the main manuscript with the heatmap because we have 16 (datasets) *20 (accuracy metrics)=320 boxplots in total. Each of them has 19 boxes representing the 19 survival approaches. That being said, we have now provided boxplots for unoc and bs in Supplementary Material as Supplementary Figure 3 and Supplementary Figure 4.

Kaplan-Meier-Estimate (KM)
I'm not quite sure I understood the authors' answer correctly. The KM does not use variable information to produce an estimate of the survival function, and I think that is why it would be interesting to include it. This would shed light on how valuable the variables are in the different data sets.

Response:
Thank you for clarifying this question.Our study does not focus on comparing feature importance differences in different methods, nor does our study compare variable contributions across the datasets. Contrasting variable importance rankings among different methods is beyond the scope

of this benchmark study. We would expect such empirical research to demonstrate that finding would be data-dependent.

Our understanding of the KM method is that Kaplan-Meier curves describe the whole population (data) or describe a subpopulation thereof. Therefore, the KM method does not provide predicted risks or survival probabilities for each individual (observation) beyond learning from to what subpopulation an individual belongs to. KM curves are commonly used to compare two or more subpopulations in the data [Ref 1]. We are aware of one benchmark study [Ref 2] that uses the KM method together with multivariate survival methods. However, when we looked into how the prediction based on KM curves was calculated, we concluded that each observation was assigned the same predicted risk in the implementation, which would contribute little to the benchmark study as a baseline method.

Ref 1: Etikan, I., S. Abubakar, and R. Alkassim. "The Kaplan-Meier estimate in survival analysis." Biom Biostatistics Int J 5.2 (2017): 00128.
Ref 2: Herrmann, Moritz, et al. "Large-scale benchmark study of survival prediction methods using multi-omics data." Briefings in bioinformatics 22.3 (2021): bbaa167.

Regarding 4. Scope and clarity
Thanks, all concerns properly addressed. No more comments.

Minor points:
- Since the authors decided to change 'framework' to 'design', note that in Figure 1b it still says 'framework'

Response:
Thanks for pointing this out. We have now changed Figure 1b title to “Benchmark design”.

- l.51 & l.54/55 appear to be redundant
Response:
Thank you for pointing this out. We have now deleted the redundant part.

- Figure 2 a and b:
  - Please elaborate more on how similarity (reflected in the dendrograms) is defined?
Response:
Similarity in the degrograms is calculated by the Euclidean distance among those metrics with respect to methods represented by a binary matrix, where 0 means that a metric is not feasible for a method and 1 means a metric is feasible for a method.

We now add the following in our Figure 2 a and b legend:
“Similarity is defined using the Euclidean distance with feasibility indicator 0 and 1. “

  - Why is the IBS more similar to Bregg's and GH C-Index than to the Brier Score?
Response:
Packages’ availability to calculate the metrics in practice). Therefore, Figure 2b does not show that Figure 2b shows whether a metric is applicable (feasible) for a particular method (based on the R IBS as a prediction accuracy metric performs similarly with Begg’s and GH C-index. Instead, it says that those three metrics (IBS, Begg’s and GH C-index) are not applicable to those methods.

- Why is the IBS not feasible for so many methods, in particular Lasso_Cox, Rdige_Cox, and CoxBoost?

Response:
To clarify, our original usage of the phrase “feasible” is in hindsight better phrased as “readily applicable”. In the revised manuscript, we now refer to how readily the methods can be evaluated by the various metrics, i.e. whether the corresponding R functions can be readily applied to our data to calculate the various evaluation metrics (Supplementary Table 1 provides details of the functions/packages used for each evaluation measurements). For example, the IBS calculation (the “crps” function in the R package “pec”; Ref 3) requires a specific type of input that is related to the training model. Currently, the acceptable models must have a “predictSurvProb” method and this is only generated for the Cox model (with “coxph” class) and Random Survival Forest model (with “rfsrc” class). We now re-phrase “feasibility” in our manuscript to “readily applicability” and modify Figure 2b legend to:

“(b) Prediction ability evaluation metric readily applicable. Row: methods; Column: evaluation metrics; legend: readily applicability where red (1) means readily applicable and blue (0) means not readily applicable.”

Ref 3: Mogensen UB, Ishwaran H, Gerds TA (2012). “Evaluating Random Forests for Survival Analysis Using Prediction Error Curves.” *Journal of Statistical Software*, **50**(11), 1–23. https://www.jstatsoft.org/v50/i11.).
