# Supplementary material for: SurvBenchmark: comprehensive benchmarking study of survival analysis methods using both omics data and clinical data
Source: Gigascience. 2022 Jul 30;11:giac071. doi: 10.1093/gigascience/giac071 (PMC9338425; doi:10.1093/gigascience/giac071)
Supplement: giac071_Supplemental_Files [file giac071_supplemental_files.zip › Supplementary Material_20220513.pdf]

Supplementary materials

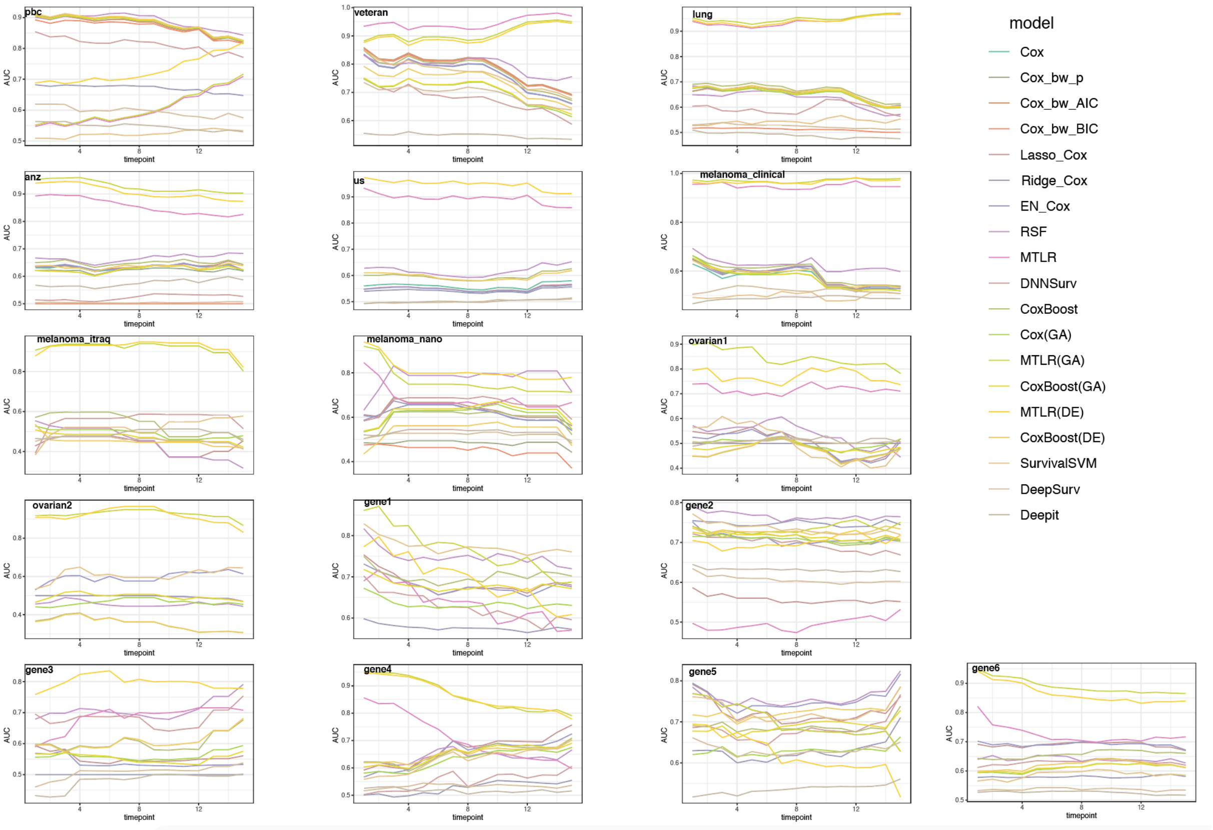

Supp Figure 1: Data dependent performance for short and long time prediction. We show the time-dependent model predictability (time-dependent AUC) for all models for each dataset across 15 time points. Different trends for those lines for different datasets indicate that model predictability for short-time versus long-time depends on the dataset.

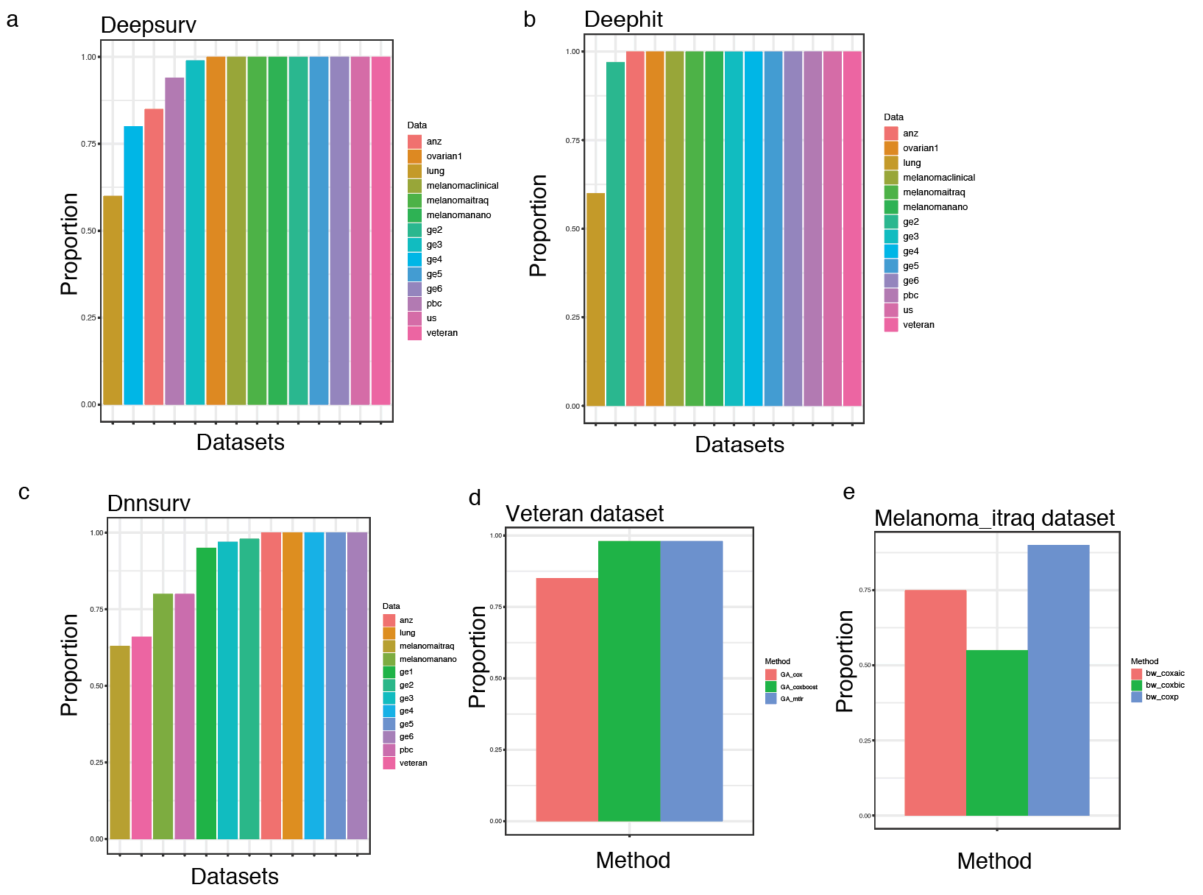

Supp Figure 2: Method reproducibility.

(a) Successful run proportions using DeepSurv. DeepSurv has a 100% completion rate for 9 out of the 14 applicable datasets. For the remaining 5 datasets, the lowest completion rate is observed for the Lung dataset and the completion rates for others are above 75%. (b) Successful run proportions using DeepHit. DeepHit has a 100% completion rate for 12 out of the 14 applicable datasets. Only GE-2 and lung datasets are not fully successful. (c) Successful run proportions using DNNSurv. DNNSurv has a 100% completion rate for 5 (ANZ, Lung, GE_4, GE_5, GE_6)out of the 12 applicable datasets. For the remaining 7 datasets, the completion rate is around 80% and completion rate was as low as 63% for the Melanoma_itraq data. (d) Clinical data Veteran. The genetic algorithm method for feature selection with Cox model, Coxboost and MTLR have successful rates 0.85, 0.98, and 0.98. (e) Omics data Melanoma_itraq. The Cox models with backward elimination methods (using AIC, BIC, P value) have successful rates 0.55, 0.75, 0.9 when using BIC, AIC, and P value, respectively.

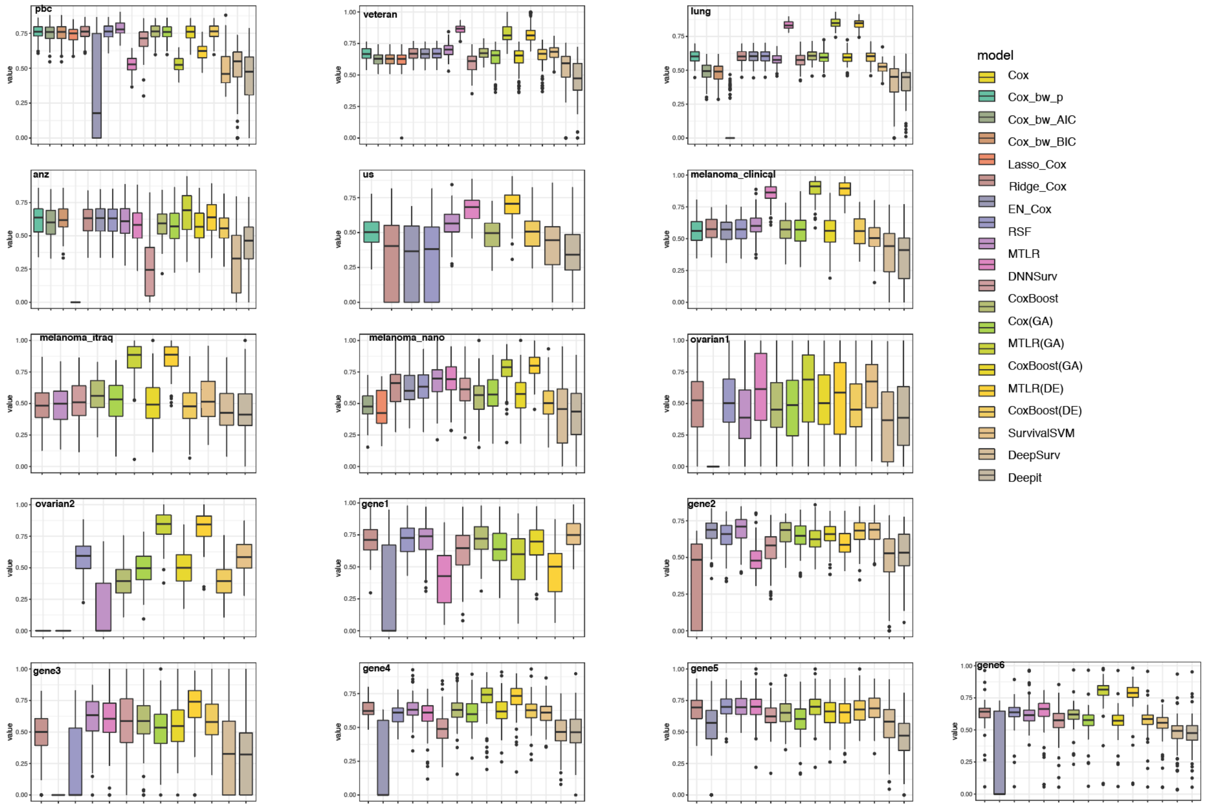

Supp Figure 3: Uno’s C-index boxplots. We show the Uno’s C-index for all models for each dataset. These boxplots provide details for readers to have a look.

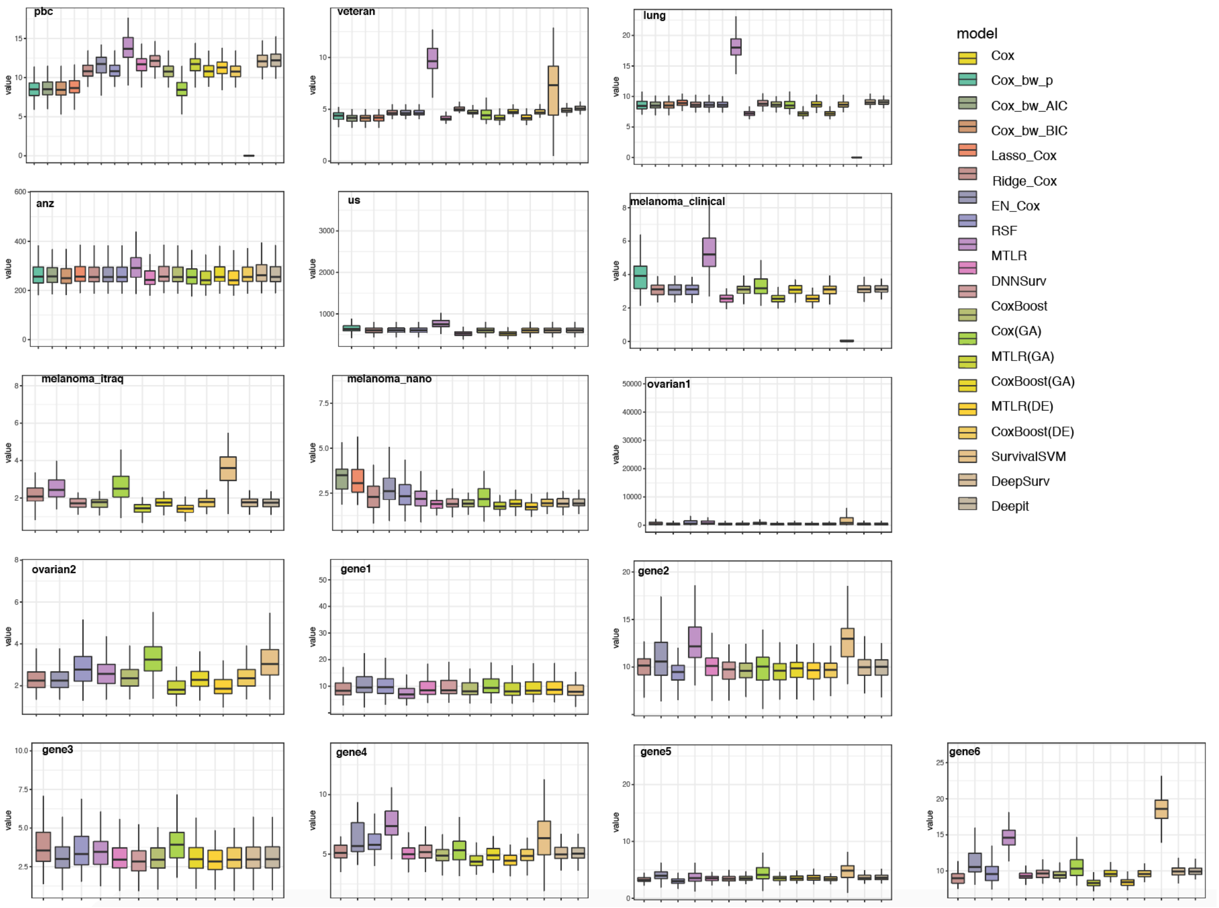

Supp Figure 4: Brier Score boxplots. We show the Brier Score for all models for each dataset. These boxplots provide details for readers to have a look.

**Supp Table 1.** Different evaluation criteria for assessing the performance models		
**Class**	**Name**	**Description**
**Model flexibility**	Type of data required	Describes the source of the data obtained, either clinical data or omics data.
	Data input	Describes the different types of data modality such as categorical, numerical or mixture.
	Data sparsity	Binary yes or no measure whether the model can handle a high level of sparsity in the data.
	Prediction ability evaluation metrics allowed	A certain evaluation metric can only be readily applied to a specific type of survival model. This is a 2-dimmodel cross predictability evaluation metric summary and whether a model can be assessed by a metric is recorded with yes or no values.
**Model predictability**	Harrell's C-index	Harrell's method to calculate the C-index. This is applied to all methods using the R function "rcorr.cens" in package "Hmisc". This value ranges from 0 to 1 with 0.5 representing random guesses and the higher the value the better the concordance, i.e. model predictability.
	Begg's C-index	Begg's method to calculate the C-index. This is applied to applicable methods using the R function "BeggC" in package "survAUC".
	Uno's C-index	Uno's method to calculate the C-index. This is applied to all methods using the R function "UnoC" in package "survAUC".
	GH C-index	Gonen and Heller's method to calculate the C-index. This is applied to applicable methods using the R function "GHCI" in package "survAUC".
	Time-dependent AUC for time t	This is the Chambless and Diao estimator of cumulative/dynamic AUC for right-censored time-to-event data for time t. This is applied to all methods using the R function "AUC.cd" in package "survAUC". The interpretation of this AUC value for a specific time t is the same as AUC in classification models.
	Brier score	This calculates the Brier score for all methods using the R function "pec" in package "pec". The smaller the value, the better the prediction.
	Integrated Brier score	This gives the scaled version of the Brier score. This value is between 0 and 1 (the smaller the value, the better the prediction) and for the constant prediction probability 0.5, the value is 0.25. This is applied using the R function"crps" in package "pec".
**Computational efficiency**	Computational time	Represents how long it takes the method to run for each dataset. This is calculated using the "Sys.time" function in R.
	Total Memory	Represents how much memory is required to run the method for each dataset. This is calculated using the "Rprof" function in R which returns the memory consumed for each sub function and then we calculate the total memory which is the sum of all of those.
**Model stability**	Reproducibility	Measures the proportions of successful runs among all those 100 runs attempted. Some methods are not fully successful for all datasets, such as DNNSurv and the corresponding successful proportions are plotted. (Supplementary Figure 2)
	SD of model predictability metrics	Measures the standard deviation (SD) of each model predictability metric. Values are ranked from 1 (smallest SD) to 20 (largest SD) for all those 20 methods within each dataset.

Supp Table 2. Linear regression model for data characteristics and Deephit model predictability (Harrell's C-index)		
**Data characteristics examined**	**Coefficients**	**P values**
n	0.0005	0.0001
p	-0.00001	0.0000
n/p	-0.002	0.001
nc	-0.161	0.052
nn	0.107	0.036
nc/nn	0.318	0.122
Intercept	0.366	0.053
Model R2: 0.726		

Supp Table 2: Examination of potential aspects that affect the model predictability using Harrell's C-index. Linear regression model for examining how variables: "n: number of observations", "p: number of features", "censoring rate", "n/p: ratio", "nn: number of numerical variables", "nc: number of categorical variables", "nc/nn: ratio" affect Harrell's C-index using the method Deephit on all datasets.

Supp Table 3. Ranking matrix

	Mean_hc	Mean_unoc	Mean_auc1	Mean_auc5	Mean_auc10	Mean_auc15	Mean_time	Mean_memory	Mean_bs	SD_hc	SD_unoc	SD_bs	SD_auc1	SD_auc5	SD_auc10	SD_auc15
Cox	6	5	6	6	6	8	5	5	16	2	1	5	3	4	4	2
Cox_bw_AIC	5	6	5	5	5	5	2	8	2	4	3	3	2	3	2	3
Cox_bw_p	4	4	4	4	4	4	3	1	4	1	2	2	1	1	1	1
Cox_bw_BIC	9	19	8	8	8	12	4	6	3	17	19	4	13	19	11	5
Coxboost	11	9	11	10	11	11	9	3	9	16	7	10	15	14	17	16
Deephit	19	17	19	19	19	19	15	15	14	7	15	13	6	9	8	9
Deepsurv	18	16	18	18	18	18	14	14	13	6	16	14	8	10	10	8
DNNsurv	10	14	12	11	10	9	10	13	1	8	9	1	11	6	5	6
Cox (GA)	15	12	14	16	13	15	13	2	7	13	5	15	16	17	16	13
Coxboost (GA)	13	10	13	13	12	13	11	7	6	15	6	9	17	16	18	18
MTLR (GA)	1	1	1	1	1	1	16	19	5	12	10	7	5	8	6	12
Coxboost (DE)	16	13	15	15	14	16	1	18	11	18	8	12	18	18	19	19
MTLR (DE)	2	2	2	2	2	2	7	16	8	10	11	6	7	5	7	14
MTLR	3	3	3	3	3	3	19	17	10	19	13	8	14	15	15	15
Lasso Cox	12	15	10	12	15	14	18	11	15	9	17	16	12	11	13	10
Ridge Cox	17	18	16	17	17	17	6	12	12	3	18	11	4	2	3	4
EN Cox	8	11	9	9	9	7	12	10	17	5	14	17	10	7	9	7
RSF	7	8	7	7	7	6	17	9	18	11	12	18	9	12	14	17
SurvivalSVM	14	7	17	14	16	10	8	4	19	14	4	19	19	13	12	11
