# Supplementary material for: SurvBenchmark: comprehensive benchmarking study of survival analysis methods using both omics data and clinical data
Source: Gigascience. 2022 Jul 30;11:giac071. doi: 10.1093/gigascience/giac071 (PMC9338425; doi:10.1093/gigascience/giac071)
Supplement: giac071_Response_to_Reviewer_Comments_Original_Submission [file giac071_response_to_reviewer_comments_original_submission.pdf]

Author's Response To Reviewer Comments

Close

Reviewer reports:
Reviewer #2: Many thanks for the very careful revision of the manuscript. Most of my concerns have been thoroughly addressed. I have only a few remarks left.

Regarding 1. Fair comparison and parameter selection
The altered study design appears much better suited to this end. Thank you very much for the effort, in particular the additional results regarding the two tuning approaches. Although I think a single simple tuning regime would be feasible here, using the default settings is reasonable and very well justified. I agree that this is much closer to what is likely to take place in practice. However, it should be more clearly emphasized that better performance may be achievable if tuning is performed.
Response:
Thank you for this comment. We agree that it is important to inform readers that suitable tuning may achieve better performance for a particular method.

We now add to line 414 the following:
"In this study we did not survey the extensive tuning procedures for those survival approaches. The main reason being that, in practice, often the default hyperparameter sets are used. Therefore, we also decided to use the default sets in our study here. However, we note that applying targeted tuning methods for a particular dataset may lead to different performances for the considered approaches. "

Regarding 2. Description
Thanks, all concerns properly addressed. No more comments.

Regarding 3. Reliability
I am aware that Figure 2c provides information to this end. I think additional boxplots which aggregate the methods' performance (e.g. for unoc and bs) over all runs and datasets would provide valuable additional information. For example, from Figure 2c one can tell that MTLR variants obtain overall higher ranks based on mean prediction performance than the deep learning methods. However, it says nothing about how large the differences in mean performance are.
Response:
Thank you for this comment. We agree that boxplots will provide complementary information especially on both the variation and the mean. In our Figure 2c, stability ranking (SD_unoc, SD_bs) measures the variation, i.e. the differences in mean performances. We have chosen to illustrate the information in the main manuscript with the heatmap because we have 16 (datasets) *20 (accuracy metrics)=320 boxplots in total. Each of them has 19 boxes representing the 19 survival approaches. That being said, we have now provided boxplots for unoc and bs in Supplementary Material as Supplementary Figure 3 and Supplementary Figure 4.

Kaplan-Meier-Estimate (KM)
I'm not quite sure I understood the authors' answer correctly. The KM does not use variable information to produce an estimate of the survival function, and I think that is why it would be interesting to include it. This would shed light on how valuable the variables are in the different data sets.
Response:
Thank you for clarifying this question.Our study does not focus on comparing feature importance differences in different methods, nor does our study compare variable contributions across the datasets. Contrasting variable importance rankings among different methods is beyond the scope of this benchmark study. We would expect such empirical research to demonstrate that finding would be data-dependent.

Our understanding of the KM method is that Kaplan-Meier curves describe the whole population (data) or describe a subpopulation thereof. Therefore, the KM method does not provide predicted risks or survival probabilities for each individual (observation) beyond learning from to what subpopulation an

individual belongs to. KM curves are commonly used to compare two or more subpopulations in the data [Ref 1]. We are aware of one benchmark study [Ref 2] that uses the KM method together with multivariate survival methods. However, when we looked into how the prediction based on KM curves was calculated, we concluded that each observation was assigned the same predicted risk in the implementation, which would contribute little to the benchmark study as a baseline method.

Ref 1: Etikan, I., S. Abubakar, and R. Alkassim. "The Kaplan-Meier estimate in survival analysis." Biom Biostatistics Int J 5.2 (2017): 00128.
Ref 2: Herrmann, Moritz, et al. "Large-scale benchmark study of survival prediction methods using multi-omics data." Briefings in bioinformatics 22.3 (2021): bbaa167.

Regarding 4. Scope and clarity
Thanks, all concerns properly addressed. No more comments.

Minor points:
- Since the authors decided to change 'framework' to 'design', note that in Figure 1b it still says 'framework'

Response:
Thanks for pointing this out. We have now changed Figure 1b title to “Benchmark design”.

- l.51 & l.54/55 appear to be redundant
Response:
Thank you for pointing this out. We have now deleted the redundant part.

- Figure 2 a and b:
- Please elaborate more on how similarity (reflected in the dendrograms) is defined?
Response:
Similarity in the degrograms is calculated by the Euclidean distance among those metrics with respect to methods represented by a binary matrix, where 0 means that a metric is not feasible for a method and 1 means a metric is feasible for a method.

We now add the following in our Figure 2 a and b legend:
“Similarity is defined using the Euclidean distance with feasibility indicator 0 and 1. “

- Why is the IBS more similar to Bregg's and GH C-Index than to the Brier Score?
Response:
Packages’ availability to calculate the metrics in practice). Therefore, Figure 2b does not show that Figure 2b shows whether a metric is applicable (feasible) for a particular method (based on the R IBS as a prediction accuracy metric performs similarly with Begg’s and GH C-index. Instead, it says that those three metrics (IBS, Begg’s and GH C-index) are not applicable to those methods.

- Why is the IBS not feasible for so many methods, in particular Lasso_Cox, Rdige_Cox, and CoxBoost?
Response:
To clarify, our original usage of the phrase “feasible” is in hindsight better phrased as “readily applicable”. In the revised manuscript, we now refer to how readily the methods can be evaluated by the various metrics, i.e. whether the corresponding R functions can be readily applied to our data to calculate the various evaluation metrics (Supplementary Table 1 provides details of the functions/packages used for each evaluation measurements). For example, the IBS calculation (the “crps” function in the R package “pec”; Ref 3) requires a specific type of input that is related to the training model. Currently, the acceptable models must have a “predictSurvProb” method and this is only generated for the Cox model (with “coxph” class) and Random Survival Forest model (with “rfsrc” class). We now re-phrase “feasibility” in our manuscript to “readily applicability” and modify Figure 2b legend to:

“(b) Prediction ability evaluation metric readily applicable. Row: methods; Column: evaluation metrics; legend: readily applicability where red (1) means readily applicable and blue (0) means not readily applicable.”

Ref 3: Mogensen UB, Ishwaran H, Gerds TA (2012). “Evaluating Random Forests for Survival Analysis Using Prediction Error Curves.” Journal of Statistical Software, 50(11), 1–23. https://www.jstatsoft.org/v50/i11.).

Close
