# Supplementary material for: SurvBenchmark: comprehensive benchmarking study of survival analysis methods using both omics data and clinical data
Source: Gigascience. 2022 Jul 30;11:giac071. doi: 10.1093/gigascience/giac071 (PMC9338425; doi:10.1093/gigascience/giac071)
Supplement: giac071_Reviewer_2_Report_Original_Submission — Moritz Herrmann -- 4/13/2022 Reviewed [file giac071_reviewer_2_report_original_submission.pdf]

Reviewer Report

Title: SurvBenchmark: comprehensive benchmarking study of survival analysis methods using both omics data and clinical data

Version: Original Submission **Date:** 4/13/2022

Reviewer name: Moritz Herrmann

Reviewer Comments to Author:

Many thanks for the very careful revision of the manuscript. Most of my concerns have been thoroughly addressed. I have only a few remarks left.
Regarding 1. Fair comparison and parameter selection
The altered study design appears much better suited to this end. Thank you very much for the effort, in particular the additional results regarding the two tuning approaches. Although I think a single simple tuning regime would be feasible here, using the default settings is reasonable and very well justified. I agree that this is much closer to what is likely to take place in practice. However, it should be more clearly emphasized that better performance may be achievable if tuning is performed.
Regarding 2. Description
Thanks, all concerns properly addressed. No more comments.
Regarding 3. Reliability
I am aware that Figure 2c provides information to this end. I think additional boxplots which aggregate the methods' performance (e.g. for unoc and bs) over all runs and datasets would provide valuable additional information. For example, from Figure 2c one can tell that MTLR variants obtain overall higher ranks based on mean prediction performance than the deep learning methods. However, it says nothing about how large the differences in mean performance are.
Kaplan-Meier-Estimate (KM)
I'm not quite sure I understood the authors' answer correctly. The KM does not use variable information to produce an estimate of the survival function, and I think that is why it would be interesting to to include it. This would shed light on how valuable the variables are in the different data sets.
Regarding 4. Scope and clarity
Thanks, all concerns properly addressed. No more comments.
Minor points:
- Since the authors decided to change 'framework' to 'design', note that in Figure 1b it still says 'framework'
- l.51 & l.54/55 appear to be redundant
- Figure 2 a and b:
- Please elaborate more on how similarity (reflected in the dendrograms) is defined?
- Why is the IBS more similar to Bregg's and GH C-Index than to the Brier Score?
- Why is the IBS not feasible for so many methods, in particular Lasso_Cox, Rdige_Cox, and CoxBoost?

Methods

Are the methods appropriate to the aims of the study, are they well described, and are necessary controls included? Choose an item.

Conclusions

Are the conclusions adequately supported by the data shown? Choose an item.

Reporting Standards

Does the manuscript adhere to the journal's guidelines on minimum standards of reporting? Choose an item.

Choose an item.

Statistics

Are you able to assess all statistics in the manuscript, including the appropriateness of statistical tests used? Choose an item.

Quality of Written English

Please indicate the quality of language in the manuscript: Choose an item.

Declaration of Competing Interests

Please complete a declaration of competing interests, considering the following questions:

- Have you in the past five years received reimbursements, fees, funding, or salary from an organisation that may in any way gain or lose financially from the publication of this manuscript, either now or in the future?
- Do you hold any stocks or shares in an organisation that may in any way gain or lose financially from the publication of this manuscript, either now or in the future?
- Do you hold or are you currently applying for any patents relating to the content of the manuscript?
- Have you received reimbursements, fees, funding, or salary from an organization that holds or has applied for patents relating to the content of the manuscript?
- Do you have any other financial competing interests?
- Do you have any non-financial competing interests in relation to this paper?

If you can answer no to all of the above, write 'I declare that I have no competing interests' below. If your reply is yes to any, please give details below.

I declare that I have no competing interests

I agree to the open peer review policy of the journal. I understand that my name will be included on my report to the authors and, if the manuscript is accepted for publication, my named report including any attachments I upload will be posted on the website along with the authors' responses. I agree for my report to be made available under an Open Access Creative Commons CC-BY license (http://creativecommons.org/licenses/by/4.0/). I understand that any comments which I do not wish to be included in my named report can be included as confidential comments to the editors, which will not be published.

Choose an item.

To further support our reviewers, we have joined with Publons, where you can gain additional credit to further highlight your hard work (see: https://publons.com/journal/530/gigascience). On publication of this paper, your review will be automatically added to Publons, you can then choose whether or not to claim your Publons credit. I understand this statement.

Yes Choose an item.
